# Supplementary material for: L-Phenylalanine is a metabolic checkpoint of human Th2 cells
Source: Cell Rep Med. 2025 Nov 26;6(12):102466. doi: 10.1016/j.xcrm.2025.102466 (PMC12765850; doi:10.1016/j.xcrm.2025.102466)
Supplement: Document S1. Figures S1–S19 [file mmc1.pdf]

Cell Reports Medicine, Volume *6*

Supplemental information

L-Phenylalanine is a metabolic checkpoint of human Th2 cells

Abhijeet J. Kulkarni, Juan Rodriguez-Coira, Nino Stocker, Urszula Radzikowska, Antonio J. García-Cívico, María Isabel Delgado Dolset, Nuria Contreras, Inés Jardón Parages, Vanesa Saiz Sanchez, Pilar Serrano, Elena Izquierdo, Cristina Gomez-Casado, Javier Sanchez-Solares, Carmela Pablo-Torres, David Obeso, Carmen Moreno-Aguilar, Maria Luisa Espinazo, Andrzej Eljaszewicz, Jana Koch, Katja Baerenfaller, Anja Heider, Ge Tan, Damir Zhakparov, Maria M. Escribese, Berta Ruiz-Leon, Cezmi A. Akdis, Rafael J. Argüello, Domingo Barber, Alma Villaseñor, and Milena Sokolowska

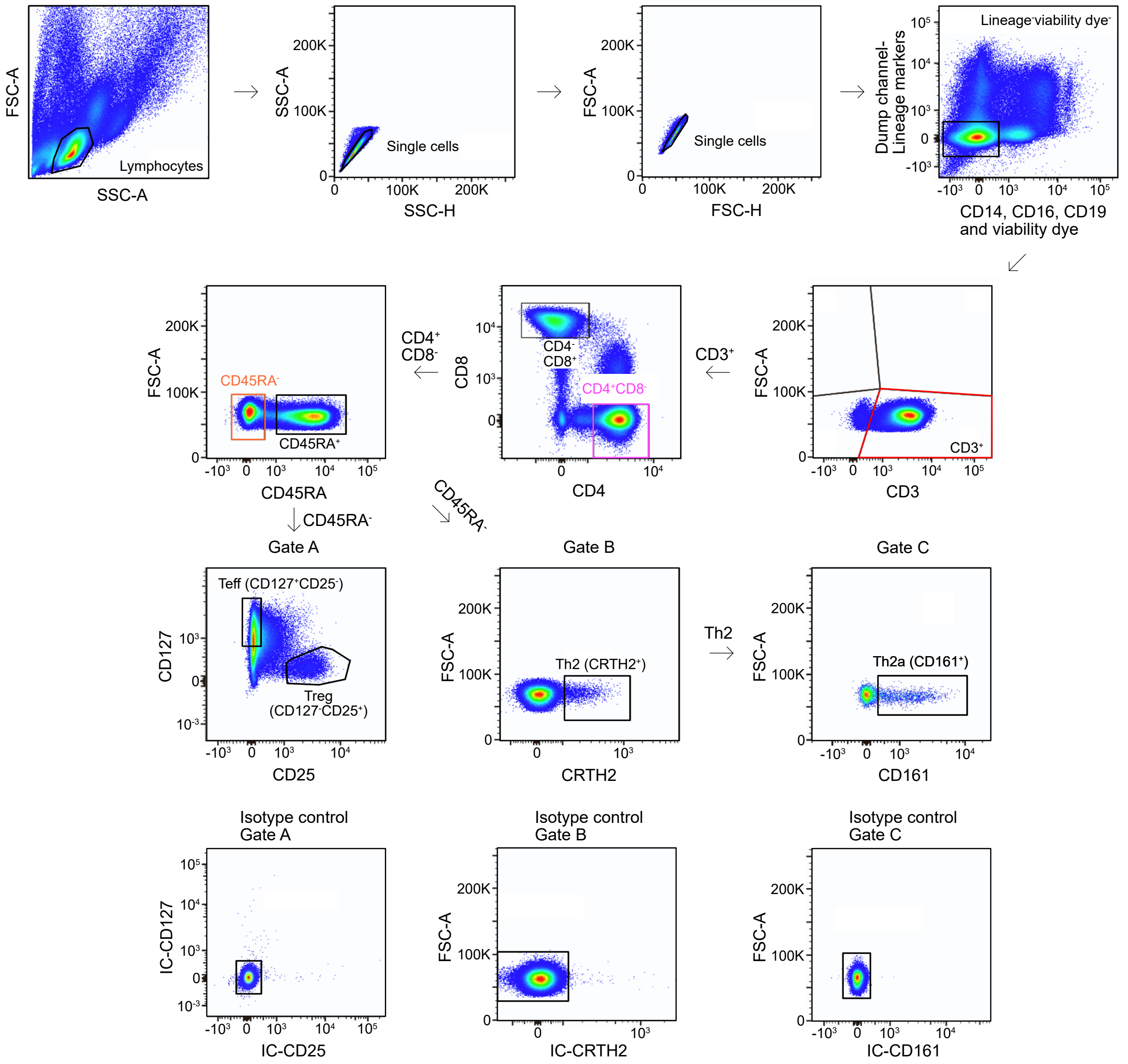

Figure S1. Gating strategy for T cells used for sorting circulating human memory CD4$^+$Teff and Treg cells and classical analysis. Related to figures 1 and 6. Briefly, T cells were gated by eliminating doublets and negatively selecting dead and other cells positive for lineage specific markers. CD3$^+$, CD4$^+$, CD8$^-$ cells were positively gated. Subsequently, memory Teff (CD45RA$^-$, CD127$^+$, CD25$^-$) and memory Treg (CD45RA$^-$, CD127$^-$, CD25$^+$) cells were sorted using gate A. Th2 and Th2a (“pathogenic”) cells were determined by the presence of CRTH2 (gate B) and double expression of CRTH2 and CD161 (gate C), respectively. Respective isotype controls are shown.

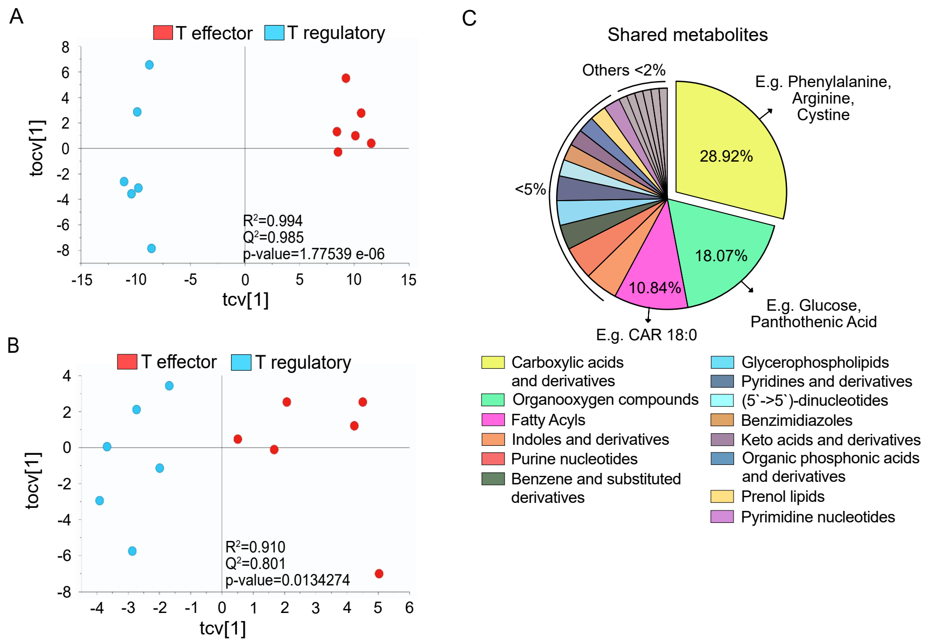

Figure S2. Unsupervised analysis of metabolomics of circulating memory CD4$^+$Teff and Treg cells. Related to figure 1. (**A**) Orthogonal Partial-Least Discriminant Analysis (OPLS-DA) models revealing separation of memory CD4$^+$Teff and Treg cells, based on all (shared and unique) metabolites. Data was logarithmic-transformed and pareto-scaled (log x Par). (**B**) OPLS-DA models of memory CD4$^+$Teff and Treg cells revealing separation based on shared metabolites only (n=133). Data was log x Par. (**C**) Pie chart representing biochemical composition of shared metabolites in memory CD4$^+$Teff and Treg cells, ordered by abundance.

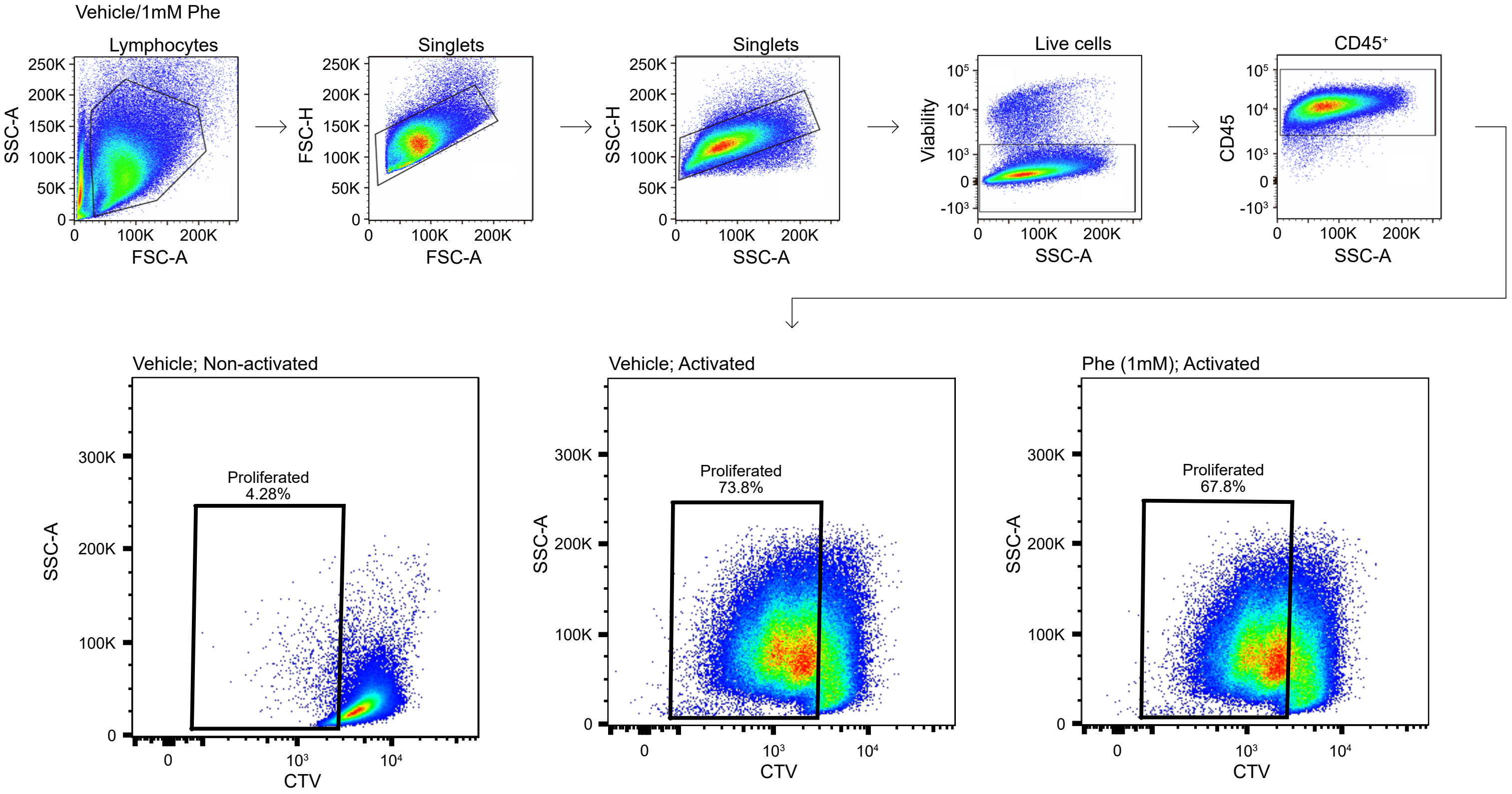

Figure S3. Gating strategy for assessment of proliferation of activated memory CD4$^+$T cells. Related to figure 3. Memory CD4$^+$T cells were stained with CTV and incubated in full medium (containing 90.9µM of Phe) with/without 1mM of additional Phe, with or without anti-CD2, anti-CD3 and anti-CD28 antibody coated beads for 72h. Subsequently, cells were stained for viability and CD45 expression with intermediate wash steps and data was acquired by flow cytometry. Briefly, memory CD4$^+$T cells were gated by eliminating doublets and negatively selecting for live cells. Subsequently, CD45$^+$ cells were positively gated. Finally, the percentage of proliferated cells was determined and CTV negative populations.

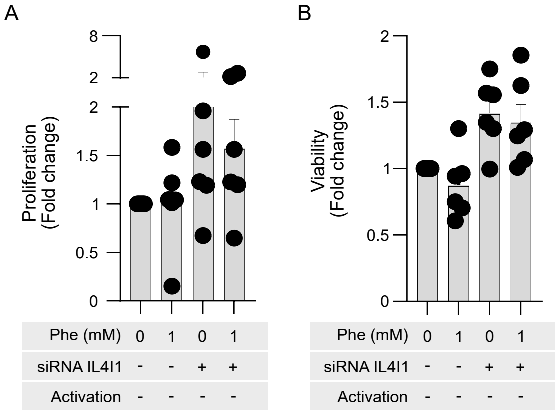

Figure S4. Proliferation of non-activated memory CD4⁺T cells following IL4I1 knockdown. Related to figure 3.
(**A**) Quantification of proliferation of control siRNA (Crtl) and siRNA IL4I1-treated human memory CD4⁺T cells from 2 independent experiments in 5 different donors, incubated in full medium (containing 90.9µM of Phe) with/without 1mM of additional Phe without CD2, CD3 and CD28 activation antibodies treatment for 24h prior to flow cytometry.
(**B**) Quantification of viability of siRNA Crtl and siRNA IL4I1-treated human memory CD4⁺T cells by flow cytometry in the same experiments as in (A). Bar graphs show a fold change in proliferation as compared to the Vehicle-treated and activated cells. Wilcoxon test was used for analysis. All data are presented as mean±SEM. Ctrl, control

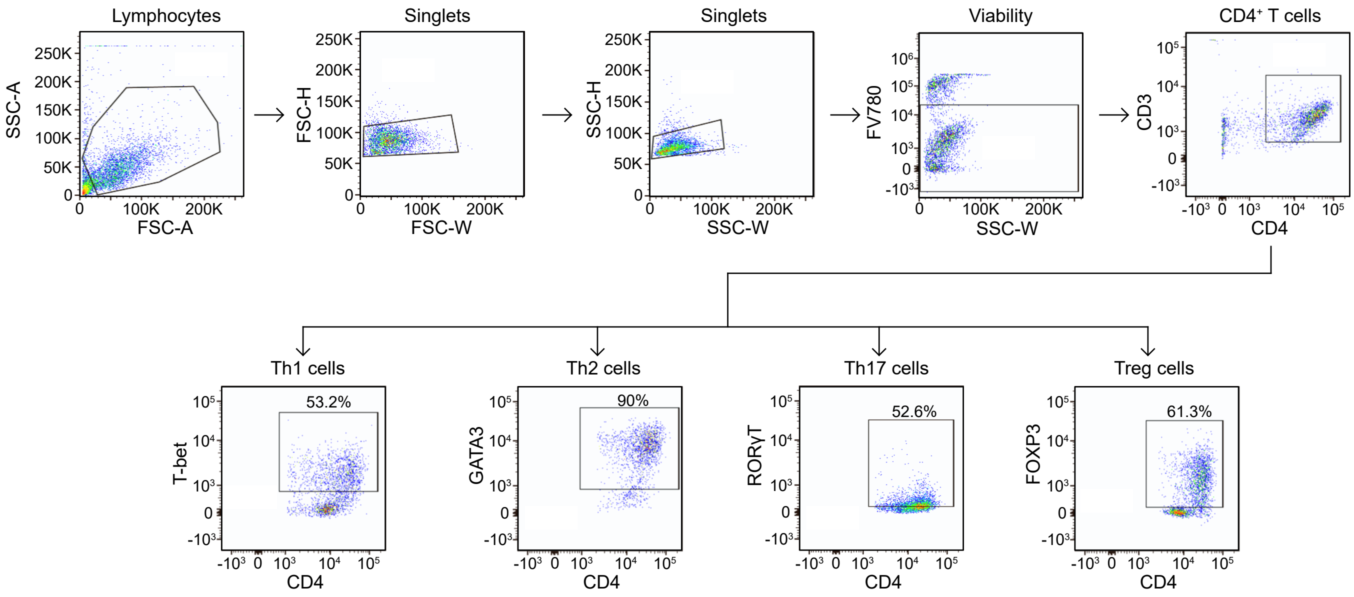

Figure S5. *In vitro* short differentiated Helper T cell subsets. Related to figure 3.
In vitro short differentiated Th1, Th2, Th17 and Treg cells were tested on day 5 of differentiation for their expression of status specific transcription factors (Th1-T-bet; Th2-GATA3; Th17- RORγT; Treg-FOXP3). Following positive selection of lymphocytes and singlets, live cells were negatively selected. $CD3^{+}CD4^{+}$ double positive cells were subsequently gated. Within this population, subset specific Transcription factor expression was used to assess differentiation.

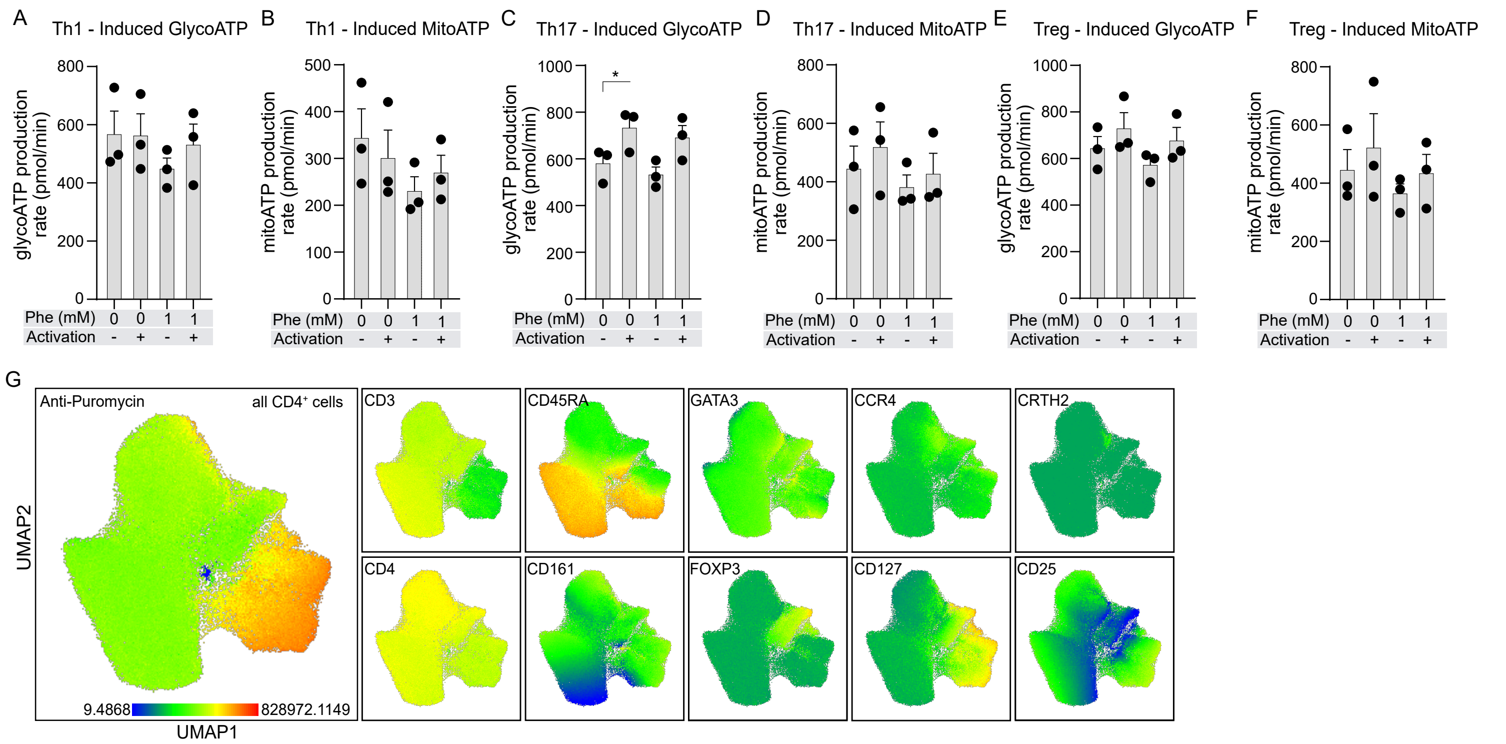

Figure S6. Energy metabolism profiling of Helper T cell subsets. Related to figure 4.
(**A-F**) GlycoATP **(A, C, E)** and MitoATP **(B, D, F)** production rates of *in vitro* short differentiated Th1, Th17, and Treg cells, respectively. Short differentiated Th cell subsets were incubated in R10+IL2 supplemented with 1mM Phe or Veh for 24h. They were subjected to Seahorse ATP Rate Assay with acute anti-CD2, anti-CD3 and anti-CD28 coated bead based activation (3:1 beads:cells). Each dot represents one subject (n=3 different subjects). Data are analysed by One-way ANOVA with Tukey correction. Bars represent mean±SEM. *p<0.05.
(**G**) Uniform Manifold Approximation and Projection (UMAP) plots of indicated protein expression in activated and non-activated $CD4^{+}$T cells. FlowJo plugin UMAP (Ver 4.0.4) was used for analysis. Frozen and thawed PBMCs were incubated in R10+IL2 supplementation of 1mM Phe or Veh with/without concurrent anti-CD2, anti-CD3, anti-CD28 antibody based activation. Subsequently, cells were harvested, washed and subjected to SCENITH depicted in Figure 4A using the panel provided in Table S9.

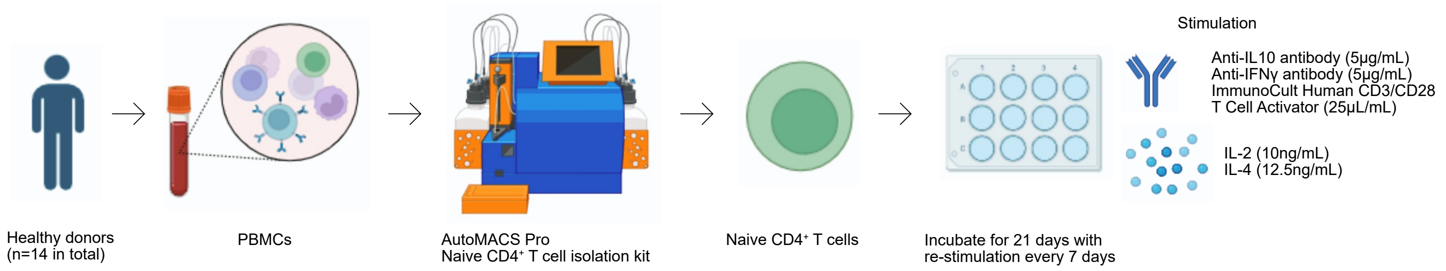

Figure S7. Methodology and timeline used for *in vitro* differentiation of naïve CD4$^+$T cells into Th2 cells. Related to figure 5 and 7. Briefly, naïve CD4$^+$T cells from PBMCs of healthy subjects are isolated and incubated in media containing IL-2 (10ng/mL), IL-4 (12.5ng/mL), anti-IFNγ antibody (5µg/mL), anti-IL-10 antibody (5µg/mL) and ImmunoCult Human CD3/CD28 T Cell Activator (25µL/mL) for 21 days with restimulation every 7 days. Further details are provided in STAR Methods. This differentiation protocol was adapted from Cousins, Lee, Staynov, 2002.[1] Prepared with Biorender.com.

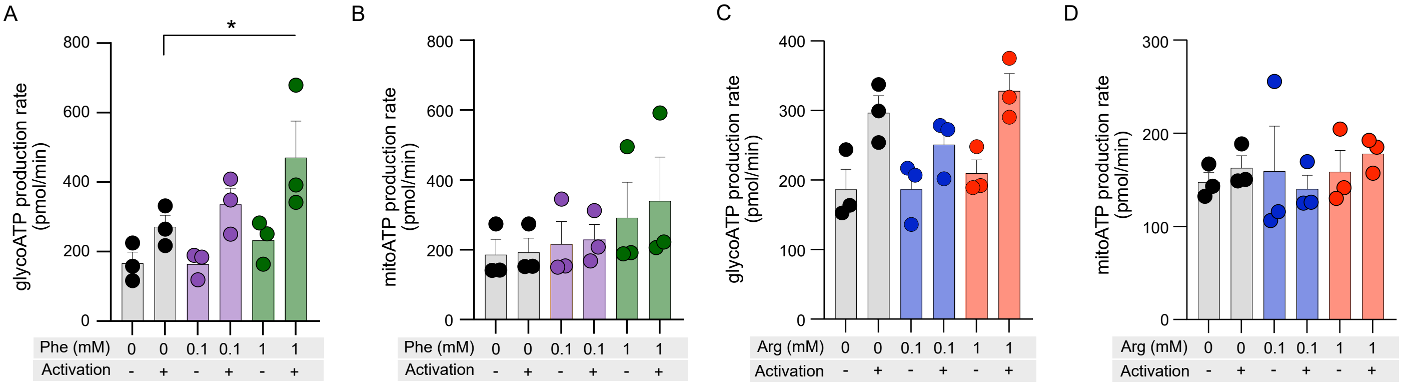

Figure S8. Quantification of induced glycoATP and mitoATP rates upon L-phenylalanine and L-arginine supplementation in *in vitro* differentiated Th2 cells. Related to figure 5.
(A-B) Quantification of induced glycoATP rate **(A)** and induced mitoATP rates **(B)** of *in vitro* differentiated Th2 cells incubated in full culture medium with/without additional Phe at concentrations of 0.1mM, 1mM, or Vehicle for 24h with/without acute CD2, CD3 and CD28 antibodies activation. **(C-D)** Quantification of induced glycoATP rate **(C)** and induced mitoATP rates **(D)** of *in vitro* differentiated Th2 cells incubated in full culture medium with/without additional Arg at concentrations of 0.1mM, 1mM, or Vehicle for 24h with/without acute CD2, CD3 and CD28 antibodies activation. Bars represent mean±SEM. Three independent experiments with 3 donors were conducted. Each dot represents the mean of more than 4 technical replicates per donor (n=3 different donors). One-way ANOVA with Sidaks´s multiple comparison correction was used to assess statistical significance. *p<0.05.

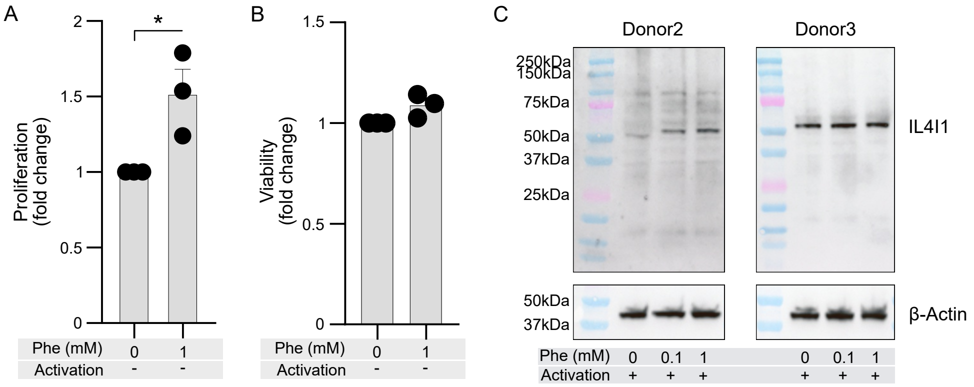

Figure S9. Effect of L-phenylalanine on the on the expression of IL4I1 and proliferation of *in vitro* differentiated Th2 cells. Related to figure 5. **(A-B)** Proliferation **(A)** and viability **(B)** of *in vitro* differentiated Th2 cells subjected to high doses of Phe. *In vitro* differentiated Th2 cells were incubated in full culture medium with or without additional supplementation of 1mM Phe without activation with CD2, CD3 and CD28 antibodies for 24h. Then, proliferation and viability were assessed using flow cytometry. Bar graphs show fold changes as compared to the Vehicle-treated, non-activated cells. Paired t-test was used for analysis. Each dot represents one donor (n=3 different donors). **(C)** Western Blot images depicting expression of IL4I1 in two additional different donors. *In vitro* long differentiated Th2 cells were incubated in media supplemented with 0.1mM and 1mM Phe or Vehicle with simultaneous activation using anti-CD2, anti-CD3 and anti-CD28 antibody coated beads for 24h. Subsequently, cells were harvested and lysed to isolate total RNA. Flow through from RNA isolation was stored and subjected to acetone precipitation to obtain total protein. WB was carried out with isolated protein using anti-IL4I1 primary antibody. Membrane was stripped and stained for β-Actin. β-Actin was used as loading control.

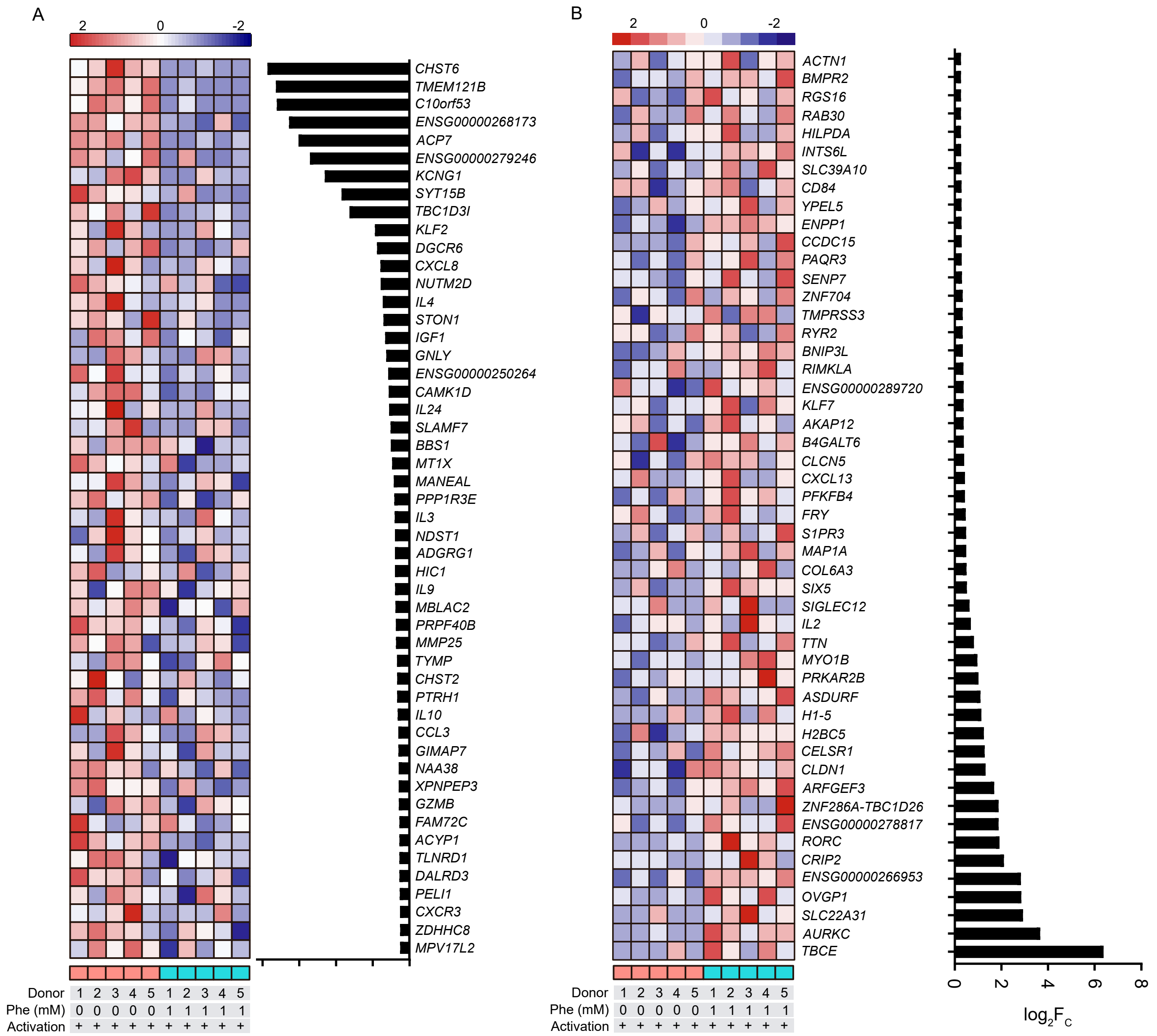

Figure S10. RNA sequencing of Phe-treated activated Th2 cells. Related to figure 5.
(A-B) Top 50 significantly downregulated **(A)** and upregulated **(B)** genes, by raw p-value < 0.05, depicted as a heatmap (left) and bar graph of fold changes (Log_2F_C) (right). Th2 cells from 5 different donors were incubated in media supplemented with 1mM Phe with or without simultaneous activation. These samples were harvested 24h later and subjected to RNA-sequencing.

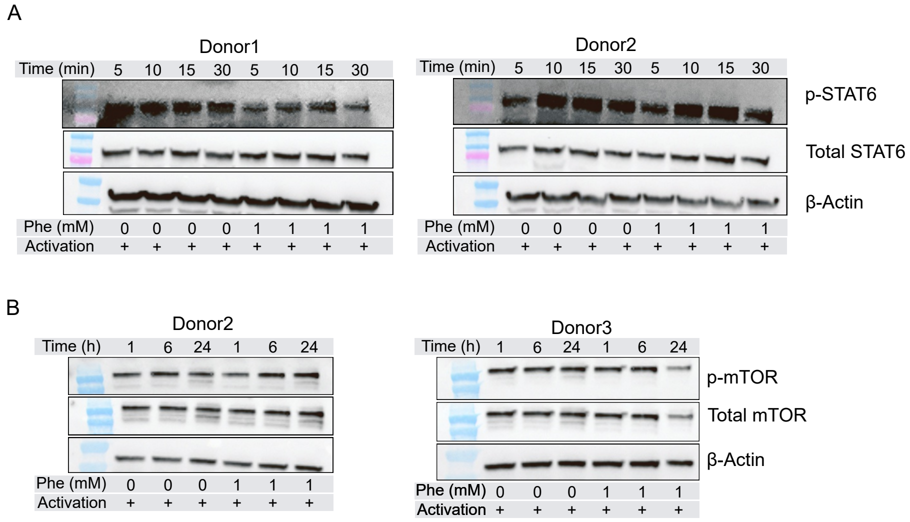

Figure S11. Effect of L-phenylalanine on phosphorylated and total STAT6 and mTOR. Related to figure 5.
(A-B) Western Blot images showing the expression of phosphorylated and total STAT6 **(A)**, and mTOR **(B)** in two additional different donors. *In vitro* long differentiated Th2 cells were incubated for mentioned time points in media supplemented with 1mM Phe or Vehicle with simultaneous activation using anti-CD2, anti-CD3 and anti-CD28 antibody coated beads. Subsequently, cells were harvested and lysed in RIPA containing protease and phosphatase inhibitors. WB was carried with cell lysate using different primary antibodies with intermediate washing and stripping steps. β-Actin serves as loading control and calibrator for quantification.

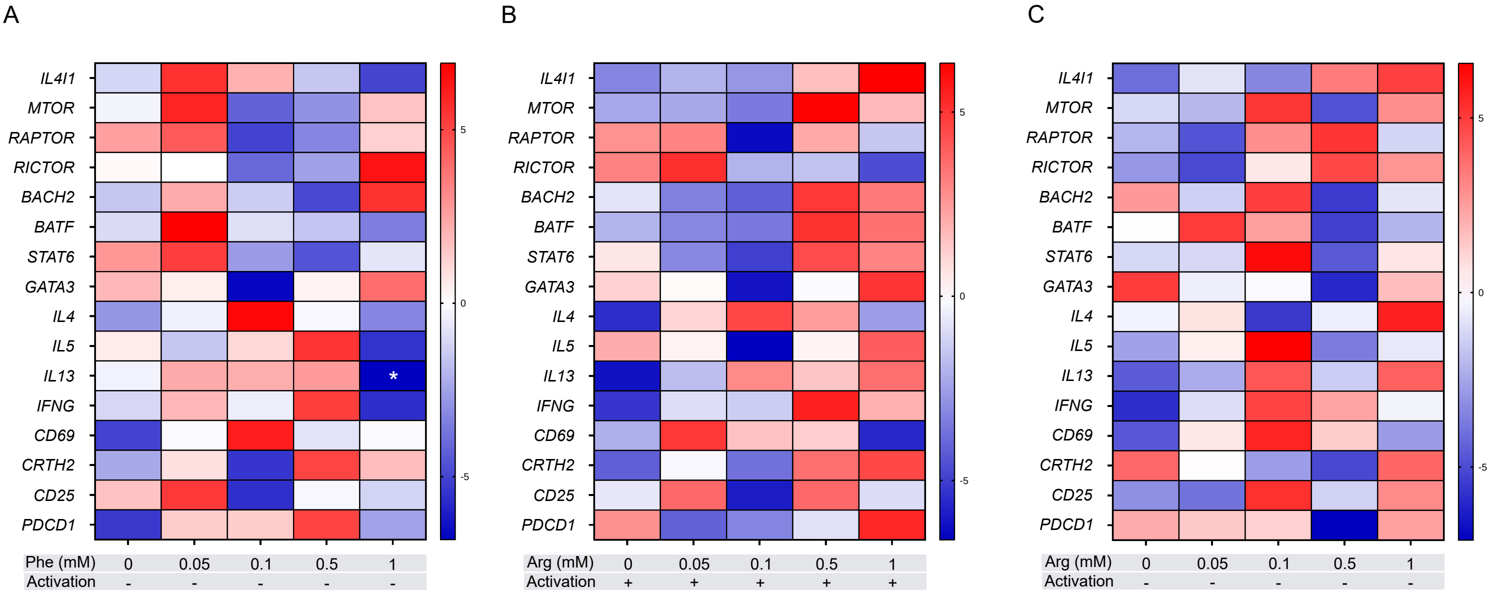

Figure S12. Effect of L-phenylalanine and L-arginine on the mRNA expression of type 2 transcription factors, cytokines, enzymes, and surface markers in Th2 cells. Related to figure 5.

(A-C) mRNA expression of transcription factors, cytokines, enzymes, and surface markers in in vitro differentiated Th2 cells treated with increasing doses of Phe **(A)**, and Arg **(B-C)** with **(B)** or without **(A, C)** activation with CD2, CD3 and CD28 antibodies for 24h.Th2 cells were incubated in full cell culture medium supplemented with Vehicle, 0.05mM, 0.1mM, 0.5mM and 1mM Phe. Following incubation, total RNA was isolated, and mRNA expression was determined using qRT-PCR. Data are analysed using One-way ANOVA with Dunnett's correction (n=6-8 different donors). Subsequently, z-scores were determined and plotted as heatmaps with individual genes represented in rows. Data are row normalized. *p<0.05.

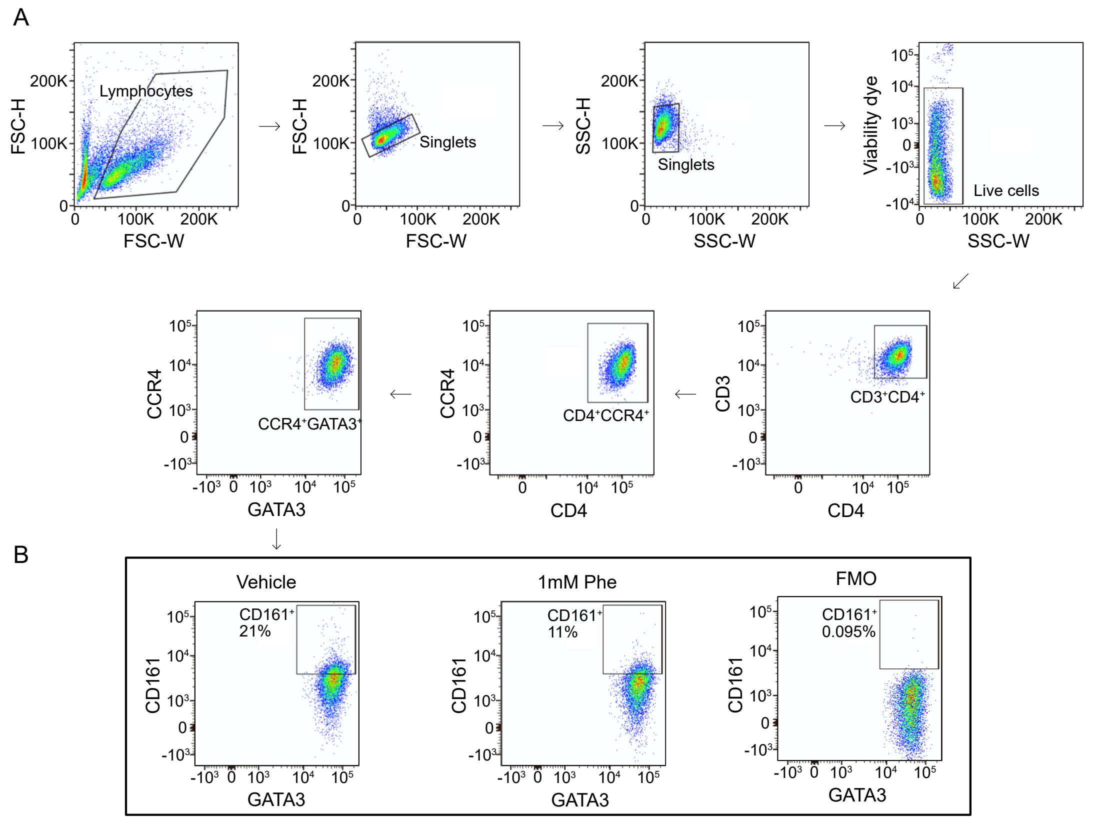

Figure S13. Effect of L-phenylalanine supplementation on Th2a cells. Related to figure 5.
(A) *In vitro* differentiated Th2 cells were incubated in media with or without supplementation of 1mM Phe for 24h with or without CD2, CD3 and CD28 activation antibodies. Following incubation, they were stained with panel provided in Table S8 and acquired on the flow cytometer. In short, live cells were negatively selected following elimination of doublets. $CD3^+CD4^+$ double positive cells were subsequently gated. Within this population, $CCR4^+$, $GATA3^+$ cells were positively selected and the frequency of $CD161^+$ Th2 (Th2a) cells was assessed in this population. **(B)** Representative flow cytometry plots showing the frequency of $CD161^+$ cells within $CD3^+CD4^+CCR4^+GATA3^+$ Th2 cells from one donor incubated in media additionally supplemented with Vehicle (left) or 1mM Phe (center). FMO control for CD161 signal has been included on the right. Pooled data from 3 different donors are shown in Figure 5P.

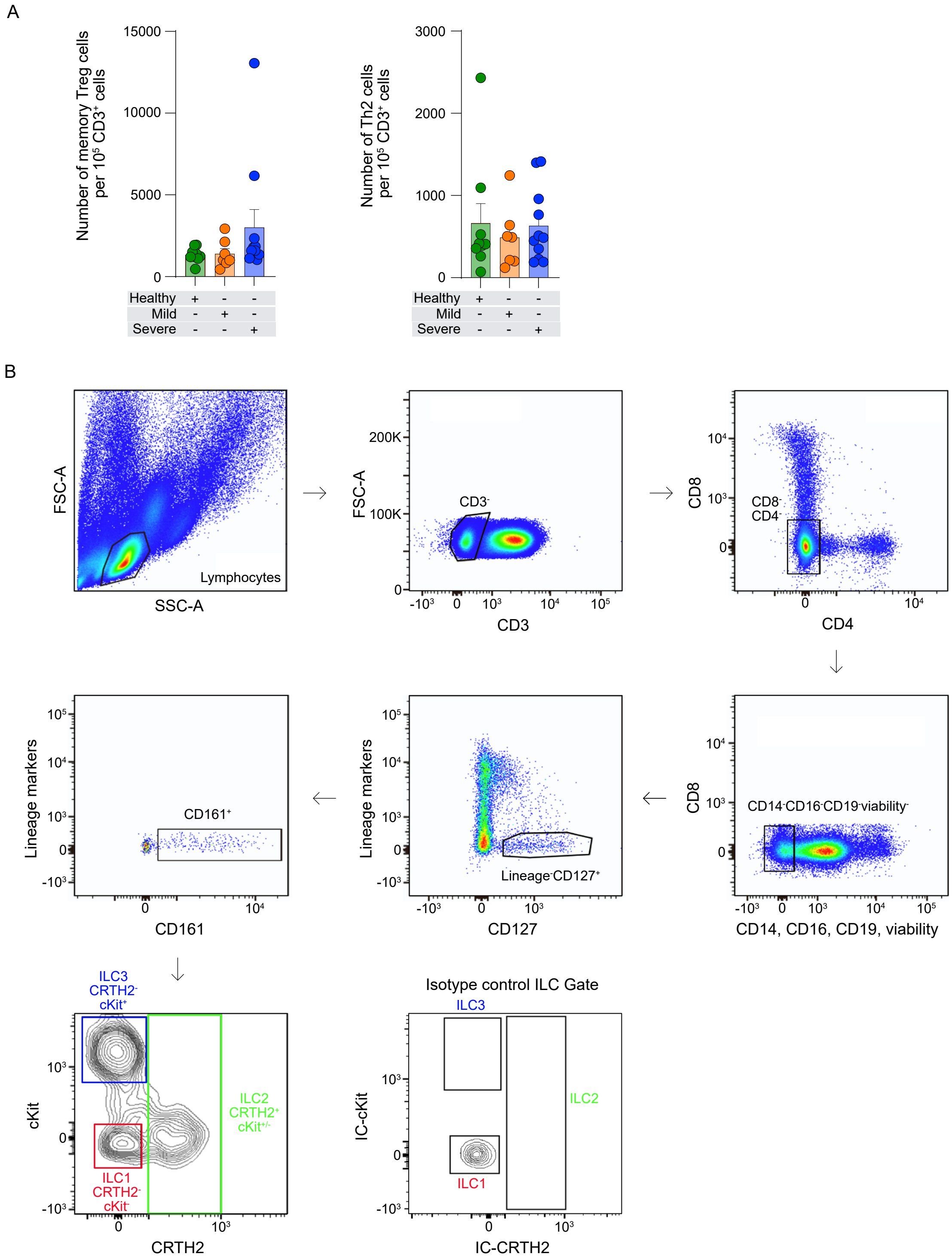

Figure S14. Flow cytometric assessment of memory cell populations and gating strategy for the study of ILCs in Cohort A samples. Related to figure 6. (A) Counts of memory Treg cells (left) and total Th2 cells (right) in healthy subjects (n=9), mild-allergic patients (n=7) and severe allergic patients (n=11) from participants in Cohort A. Counts were determined using flow cytometry and analysed with One-way ANOVA with Tukey correction. Bars represent mean±SEM and each dot represents one donor. **(B)** Gating strategy for assessment of innate lymphoid cells (ILCs) followed in classical analysis. ILCs were gated by eliminating doublets and all other immune cell lineages. Within $CD127^+$ and $CD161^+$ cells different classes of ILCs could be discerned by differential expression of CRTH2 and cKit: ILC1 ($CRTH2^-$, $cKit^-$), ILC2 ($CRTH2^+$, $cKit^{+/-}$), and ILC3 ($CRTH2^-$, $cKit^+$).

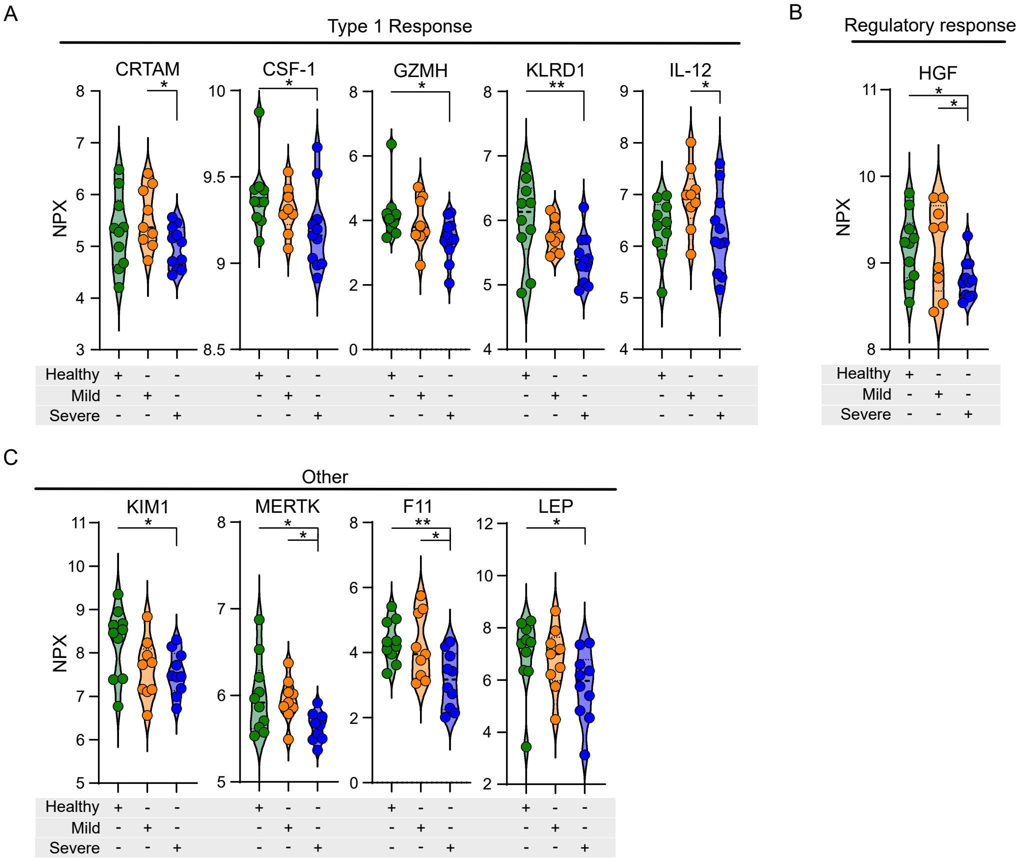

Figure S15. Systemic type 2 inflammatory profile of subjects from Cohort A by serum proteomics. Related to figure 6.
(A-C) Violin plots of differentially expressed type 1 response **(A)**, regulatory response **(B)** and key inflammation associated **(C)** proteins in the serum of controls (n=10), mild (n=9) and severe (n=10) allergic patients (Cohort A) analyzed by Proximity Extension Assay (PEA) and presented as NPX. One-way ANOVA with Fishers LSD test was used to compare differences between groups. All bars represent the mean±SEM and each dot represents one donor. *$p<0.05$, **$p<0.01$.

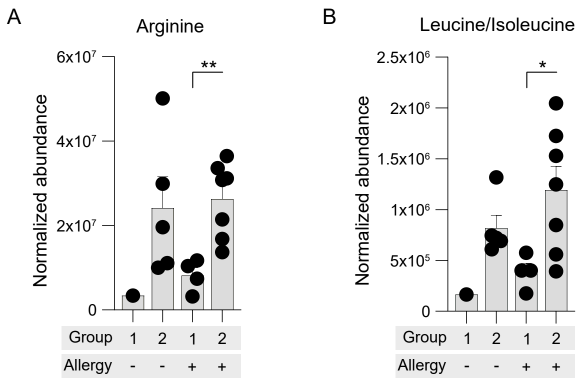

Figure S16. Quantification of Arg and leucine/isoleucine in Teff cells by metabolomics of Cohort A subjects. Related to figure 6. **(A-B)** Abundance of Arg **(A)** and Leu **(B)** in group 1 (control, n = 1; severe allergy, n = 4) and group 2 (control, n = 5; mild allergy = 1, severe allergy, n = 6) (subset of cohort A). Unpaired t-test was used to compare differences between groups. All graphs represent the mean±SEM. *p<0.05, **p<0.01.

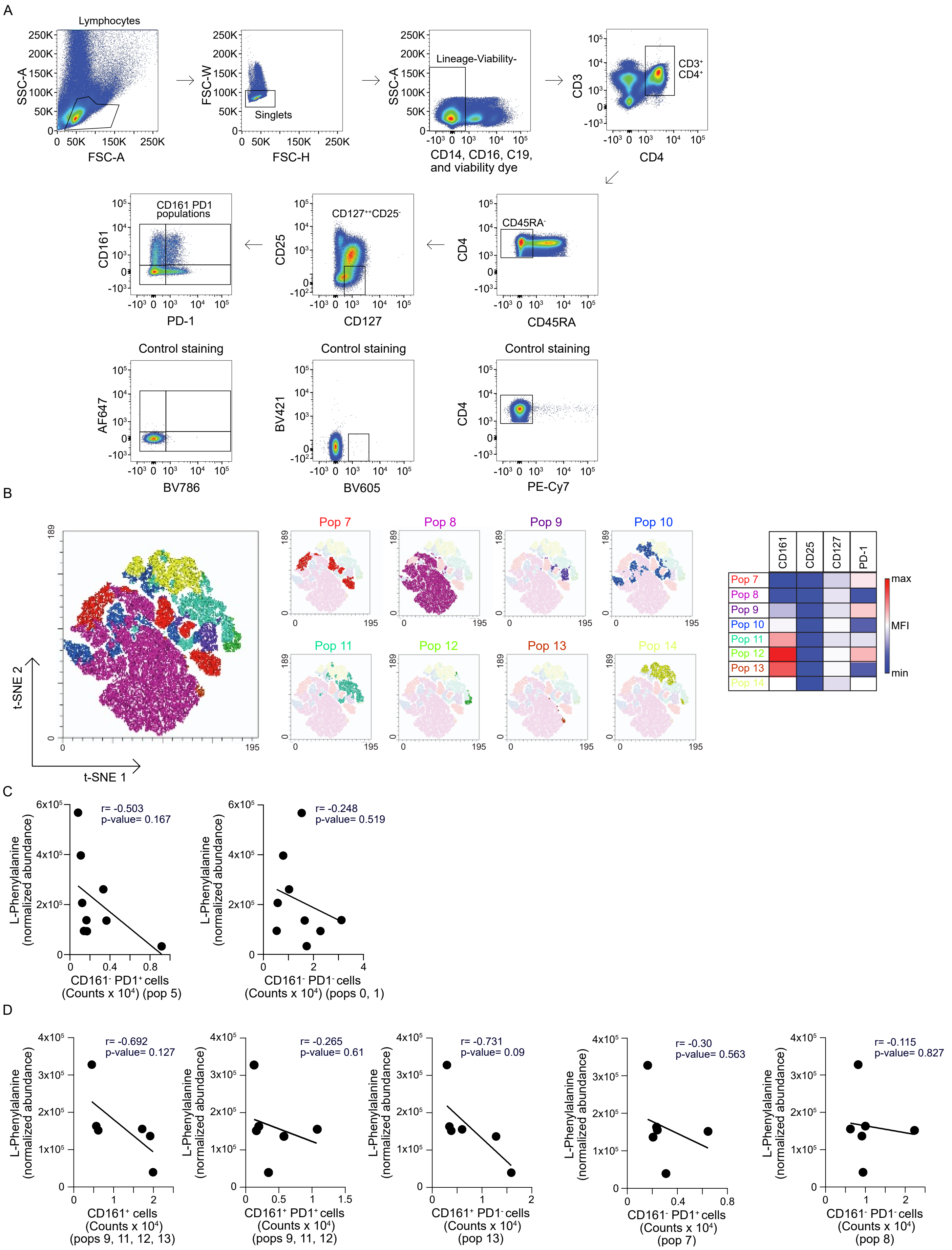

A
Lymphocytes
SSC-A
FSC-A
FSC-W
FSC-H
Singlets
Lineage-Viability-
SSC-A
CD14, CD16, C19, and viability dye
CD3
CD4
CD3+ CD4+
CD45RA-
CD4
CD45RA
CD127++CD25-
CD25
CD127
CD161 PD1 populations
CD161
PD-1
Control staining
AF647
BV786
Control staining
BV421
BV605
Control staining
CD4
PE-Cy7
B
t-SNE 2
t-SNE 1
Pop 7
Pop 8
Pop 9
Pop 10
Pop 11
Pop 12
Pop 13
Pop 14
CD161
CD25
CD127
PD-1
max
MFI
min
C
r= -0.503
p-value= 0.167
L-Phenylalanine (normalized abundance)
CD161- PD1+ cells (Counts x 10^4) (pop 5)
r= -0.248
p-value= 0.519
L-Phenylalanine (normalized abundance)
CD161- PD1- cells (Counts x 10^4) (pops 0, 1)
D
r= -0.692
p-value= 0.127
L-Phenylalanine (normalized abundance)
CD161+ cells (Counts x 10^4) (pops 9, 11, 12, 13)
r= -0.265
p-value= 0.61
L-Phenylalanine (normalized abundance)
CD161+ PD1+ cells (Counts x 10^4) (pops 9, 11, 12)
r= -0.731
p-value= 0.09
L-Phenylalanine (normalized abundance)
CD161+ PD1- cells (Counts x 10^4) (pop 13)
r= -0.30
p-value= 0.563
L-Phenylalanine (normalized abundance)
CD161- PD1+ cells (Counts x 10^4) (pop 7)
r= -0.115
p-value= 0.827
L-Phenylalanine (normalized abundance)
CD161- PD1- cells (Counts x 10^4) (pop 8)

Figure S17. Unbiased 2-dimensional flow cytometric analysis of memory CD4$^+$Teff cells from severe allergic patients and healthy controls (subset of Cohort A). Related to figure 6. (A) Gating strategy used for t-SNE and subsequent classical analysis to determine frequencies of populations based on expression levels of CD161, and PD-1 by flow cytometric assessment. **(B)** tSNE plot of unbiased 2-dimensional flow cytometric analysis of memory CD4$^+$Teff cells (CD3$^+$CD4$^+$CD45RA$^-$CD127$^+$CD25$^-$) from healthy controls (subset of Cohort A) identifying seven subpopulations based on the expression levels of CD161, and PD-1. **(C)** Pearson correlation of normalized abundance of intracellular Phe, measured in memory CD4$^+$Teff cells from patients with severe allergy, with total counts of CD161$^-$ populations within memory CD4$^+$Teff cells (subset of Cohort A). **(D)** Pearson correlation of normalized abundance of intracellular Phe, measured in memory CD4$^+$Teff cells from healthy controls, with total counts of CD161$^+$ and CD161$^-$ populations within memory CD4$^+$Teff cells (subset of Cohort A).

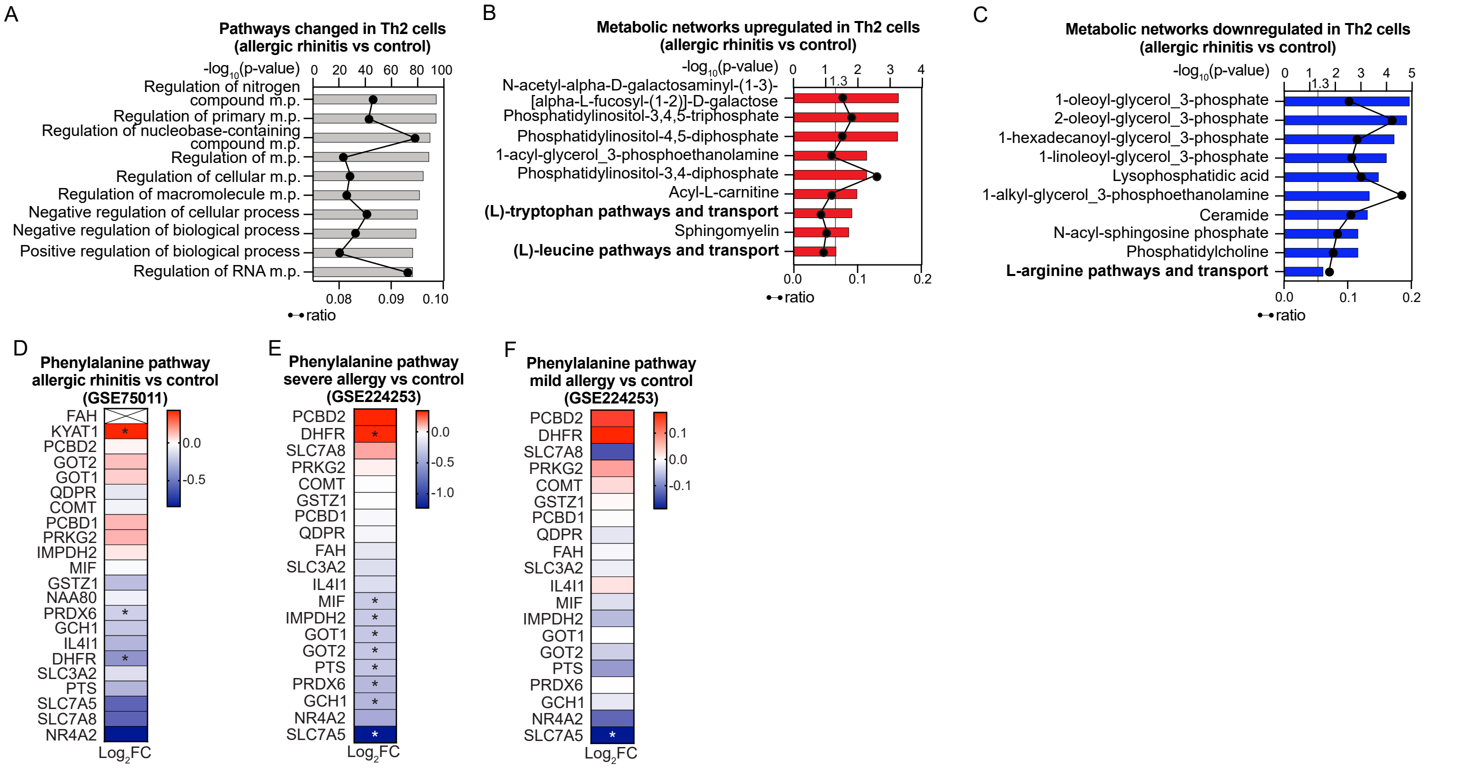

Figure S18. Expression of L-Phenylalanine metabolism related genes in previously published datasets (Cohorts B and C). Related to figure 7. (**A-C**) Top significantly enriched pathways (**A**), significantly upregulated (**B**) and significantly downregulated (**C**) metabolic networks within differentially expressed genes (DEG, $p<0.05$) in allergic rhinitis patients compared to controls (control n=15, allergic rhinitis n=25) from GSE75011[2] (Cohort B). Black line represents ratio of genes in experiment over complete pathway set. All data available in tables S21, S23 and S25, respectively. (**D**) Phe metabolism and transport pathway heatmap showing fold change (log2FC) of differentially expressed genes in allergic rhinitis patients (n=25) compared to controls (n=15) from GSE75011[2] (Cohort B). (**E**) Significant differentially expressed genes related to Phe metabolism in severe allergy patients (n=7) in comparison to healthy controls (n=8) from GSE224253[3] (Cohort C). (**F**) Significant differentially expressed genes related to Phe metabolism in mild allergy patients (n=9) in comparison to healthy controls (n=8) from GSE224253[3] (Cohort C). (B-F) Upregulated and downregulated genes are shown in red and blue, respectively. (D-F) Pathway curated and adapted from GSEA and MSigDB Database.

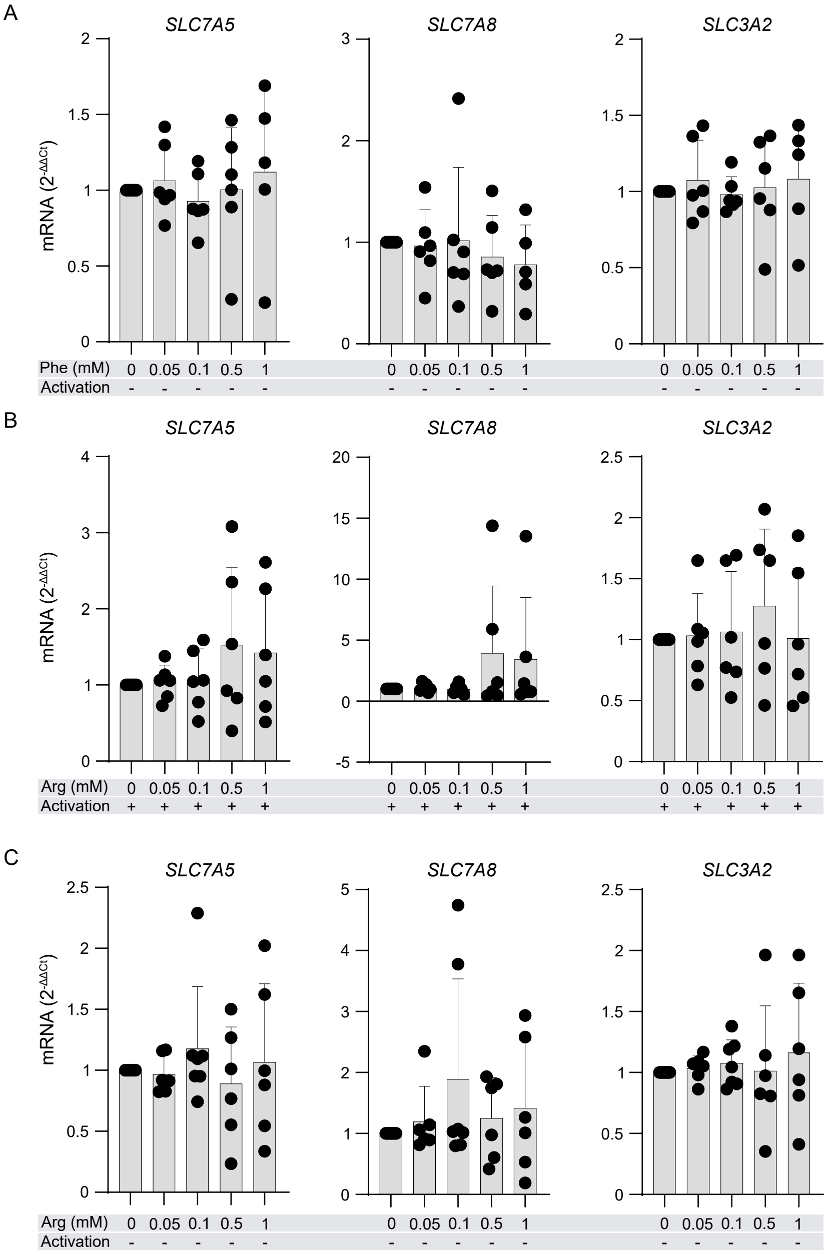

Figure S19. Effect of L-phenylalanine and L-arginine on the mRNA expression of *SLC7A5* (LAT1), *SLC7A8* (LAT2) and *SLC3A2* (CD98) in Th2 cells. Related to figure 7. (A-C) *In vitro* differentiated Th2 cells were treated with additional Phe without CD2, CD3 and CD28 activation **(A)** and additional Arg with **(B)** or without **(C)** CD2, CD3 and CD28 activation for 24h. Following incubation, mRNA expression was determined using qRT-PCR (n=6-8 different donors). Data are analysed using One-way ANOVA with Dunnett's correction (n=6-8 different donors). Bars represent mean±SEM.

Supplemental references list:

1. Cousins, D.J., Lee, T.H., and Staynov, D.Z. (2002). Cytokine coexpression during human Th1/Th2 cell differentiation: direct evidence for coordinated expression of Th2 cytokines. J Immunol *169*, 2498-2506. 10.4049/jimmunol.169.5.2498.

2. Seumois, G., Zapardiel-Gonzalo, J., White, B., Singh, D., Schulten, V., Dillon, M., Hinz, D., Broide, D.H., Sette, A., Peters, B., and Vijayanand, P. (2016). Transcriptional Profiling of Th2 Cells Identifies Pathogenic Features Associated with Asthma. J Immunol 197, 655-664. 10.4049/jimmunol.1600397.

3. Pablo-Torres, C., Garcia-Escribano, C., Romeo, M., Gomez-Casado, C., Arroyo Solera, R., Bueno-Cabrera, J.L., Del Mar Reano Martos, M., Iglesias-Cadarso, A., Tarin, C., Agache, I., et al. (2023). Transcriptomics reveals a distinct metabolic profile in T cells from severe allergic asthmatic patients. Front Allergy 4, 1129248. 10.3389/falgy.2023.1129248.
