# Supplementary material for: L-Phenylalanine is a metabolic checkpoint of human Th2 cells
Source: Cell Rep Med. 2025 Nov 26;6(12):102466. doi: 10.1016/j.xcrm.2025.102466 (PMC12765850; doi:10.1016/j.xcrm.2025.102466)
Supplement: Document S2. Article plus supplemental information [file mmc30.pdf]

Cell Reports
Medicine

Article

L-Phenylalanine is a metabolic checkpoint of human Th2 cells

Graphical abstract

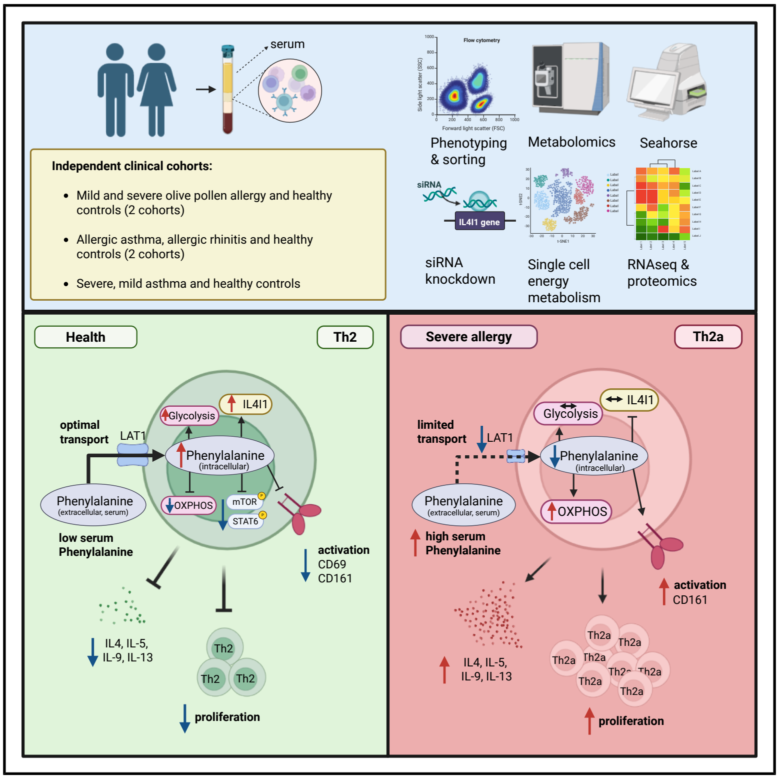

Authors

Abhijeet J. Kulkarni,
Juan Rodriguez-Coira, Nino Stocker, ...,
Domingo Barber, Alma Villaseñor,
Milena Sokolowska

Correspondence

milena.sokolowska@siaf.uzh.ch

In brief

Kulkarni et al. show that L-phenylalanine reprograms energy metabolism and restrains growth and differentiation of human Th2 cells. Patients with severe allergic diseases feature reduced L-phenylalanine transport into CD4^{+} T cells, leading to expansion of pathogenic Th2 cells.

Highlights

- L-phenylalanine boosts glycolysis and limits oxidative phosphorylation in Th2 cells
- L-phenylalanine limits proliferation of Th2 cells via interleukin-4 induced 1
- L-phenylalanine limits Th2 reprogramming by inhibiting STAT6 and mTOR signaling
- LAT1-dependent L-phenylalanine transport in Th2 cells is impaired in severe allergy

Kulkarni et al., 2025, Cell Reports Medicine 6, 102466
December 16, 2025 © 2025 The Author(s). Published by Elsevier Inc.
https://doi.org/10.1016/j.xcrm.2025.102466

CellPress

Cell Reports Medicine

CellPress
OPEN ACCESS

Article

L-Phenylalanine is a metabolic checkpoint of human Th2 cells

Abhijeet J. Kulkarni,[1,14] Juan Rodriguez-Coira,[1,2,3,14] Nino Stocker,[1] Urszula Radzikowska,[1] Antonio J. García-Cívico,[2,3] María Isabel Delgado Dolset,[2,3] Nuria Contreras,[2,3] Inés Jardón Parages,[1] Vanesa Saiz Sanchez,[4,5] Pilar Serrano,[4,5] Elena Izquierdo,[2] Cristina Gomez-Casado,[2] Javier Sanchez-Solares,[2] Carmela Pablo-Torres,[2,6] David Obeso,[2,3] Carmen Moreno-Aguilar,[4,5] Maria Luisa Espinazo,[4,5] Andrzej Eljaszewicz,[7,8] Jana Koch,[1,9,10] Katja Baerenfaller,[1,9] Anja Heider,[1] Ge Tan,[1,11] Damir Zhakparov,[1,9] Maria M. Escribese,[2] Berta Ruiz-Leon,[4,5] Cezmi A. Akdis,[1,12] Rafael J. Argüello,[13] Domingo Barber,[2] Alma Villaseñor,[2,3,15] and Milena Sokolowska[1,15,16,*]
[1]Swiss Institute of Allergy and Asthma Research (SIAF), University of Zurich, Davos, Switzerland
[2]Departamento de Ciencias Médicas Básicas, Facultad de Medicina, Instituto de Medicina Molecular Aplicada – Nemesio Díez (IMMA-ND), Universidad San Pablo-CEU, CEU Universities, Madrid, Spain
[3]Centro de Metabolómica y Bioanálisis (CEMBIO), Facultad de Farmacia, Universidad San Pablo-CEU, CEU Universities, Urbanización Montepríncipe, Boadilla del Monte 28660, Spain
[4]UGC Immunología y Alergia, Hospital Universitario Reina Sofía de Córdoba, Córdoba, Spain
[5]Instituto Maimónides de Investigación Biomédica de Córdoba (IMIBIC)/Hospital Universitario Reina Sofía/Universidad de Córdoba, Córdoba, Spain
[6]Department of Environmental Health, Harvard T.H. Chan School of Public Health, Boston, MA, USA
[7]Centre of Regenerative Medicine, Medical University of Bialystok, ul. Waszyngtona 15B, 15-269 Bialystok, Poland
[8]Tissue and Cell Bank, Medical University of Bialystok Clinical Hospital, Bialystok, Poland
[9]Swiss Institute of Bioinformatics, Lausanne, Switzerland
[10]Institute of Theoretical Medicine, University of Augsburg, Augsburg, Germany
[11]Functional Genomics Center Zurich, ETH Zurich/University of Zurich, Zurich, Switzerland
[12]Christine Kühne – Center for Allergy Research and Education (CK-CARE), Davos, Graubünden, Switzerland
[13]Aix Marseille University, CNRS, INSERM, CIML, Centre d'Immunologie de Marseille-Luminy, Marseille, France
[14]These authors contributed equally
[15]Senior author
[16]Lead contact
*Correspondence: milena.sokolowska@siaf.uzh.ch
https://doi.org/10.1016/j.xcrm.2025.102466

SUMMARY

After the primary response, circulating memory CD4$^+$T effector and T regulatory (Treg) cells regulate recall responses, typically impaired in allergy. We discovered distinct metabolomes of these cells in humans, differentially enriched in phenylalanine-related metabolites. Energy metabolism assessment in *in vitro* and *ex vivo* single-cell analyses revealed that increased intracellular L-phenylalanine boosts glycolysis while limiting oxidative phosphorylation (OXPHOS) in CD4$^+$T, memory CD4$^+$T, and Th2 cells, but not in Th1, Th17, or Treg cells. L-phenylalanine also restrains proliferation of memory CD4$^+$T, Th2, and Th17 cells in an IL4I1-dependent manner and limits Th2 differentiation via inhibition of STAT6 and mechanistic target of rapamycin (mTOR) signaling. RNA sequencing, metabolomics, flow cytometry, and proteomics, validated both *in vitro* and across patient cohorts, revealed impaired LAT1-dependent transport of L-phenylalanine into Th2 cells in allergy, with increased intracellular processing accompanied by expansion of pathogenic Th2 cells. Thus, our study identifies L-phenylalanine as a checkpoint in Th2 cell development, energy metabolism, and function.

INTRODUCTION

Upon activation, naive CD4$^+$T cells clonally expand and differentiate into several specialized effector (Teff) subsets, including T helper (Th) 1, Th2, and Th17, depending on the nature of antigen challenge and cues from the microenvironment.[1] Regulatory CD4$^+$CD25$^+$T (Treg) cells arise either from thymus or from peripheral conversion of conventional T cells.[2,3] After initial clonal expansion, majority of CD4$^+$Teff cells are removed in absence of antigen, but a fraction of them form long-lived memory T cells, persisting in tissues, lymphatic organs, and, importantly, circulation.[1,4,5] Memory CD4$^+$T cell subsets mount quicker and greater recall Teff and Treg responses after subsequent encounter with the antigen.[1] Quiescent naive and memory CD4$^+$T cells and Treg cells tend to primarily use fatty acid oxidation (FAO) and oxidative phosphorylation (OXPHOS),[6–9]

© 2025 The Author(s). Published by Elsevier Inc.
This is an open access article under the CC BY license (http://creativecommons.org/licenses/by/4.0/).

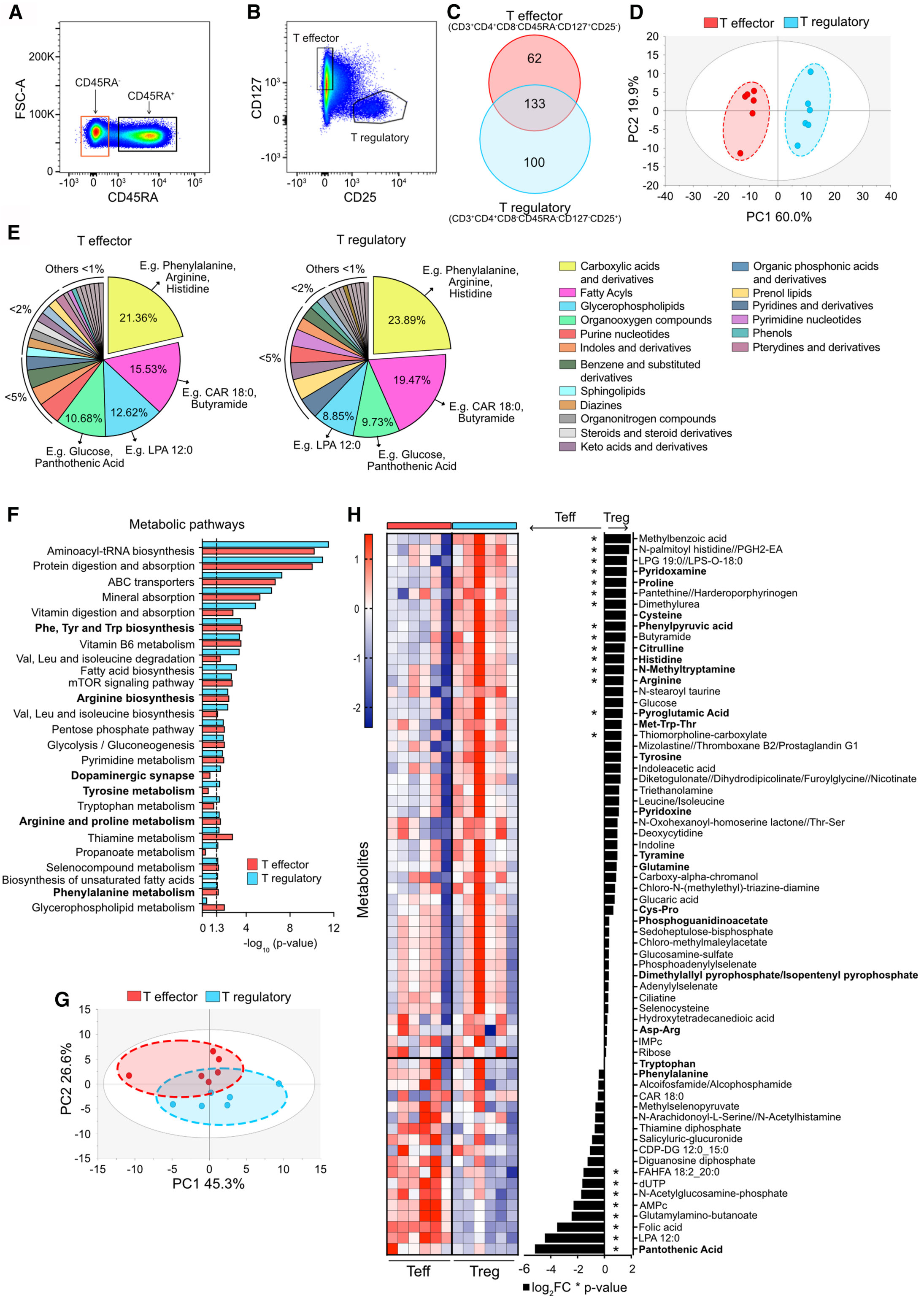

Figure 1. Metabolomics of circulating human memory CD4^{+} T effector and T regulatory cells reveals distinct metabolic profiles, enriched in phenylalanine and arginine metabolic pathways

(A and B) Intermediate gate depicting memory (CD45RA^{-}) and naive (CD45RA^{+}) CD3^{+}CD4^{+}T cells (A) and final sorting gate (B) of circulating memory CD4^{+}T effector and T regulatory cells.

(legend continued on next page)

although, in human Treg cells, glycolysis and fatty acid metabolism are also active and needed for their suppressive functions.[10,11] Post-antigen encounter, co-stimulation, and cytokine signaling, CD4+Teff and Treg subsets undergo further changes in their glucose, amino acid, and fatty acid metabolism fueling subset-specific proliferation and function.[7,12–16] Maintaining equilibrium among memory CD4+Teff and Treg subsets is essential for homeostasis and prevention of inflammatory or autoimmune diseases. However, while metabolic requirements of naive CD4+T cells upon initial activation and differentiation into effector and regulatory subsets are well studied,[17,18] much less is known about metabolic programs of circulating human memory CD4+Teff and Treg cells. T cell function and metabolism are controlled by amino acids via acquisition from extracellular milieu through cellular transporters, intracellular storage, and recycling within different metabolic pathways, especially glycolysis, tricarboxylic acid cycle, and OXPHOS.[13,19–21] Expression of amino acid transporters such as LAT1 (*SLC7A5*), LAT2 (*SLC7A8*), CD98 (*SLC3A2*), *SLC1A5*, *SLC38A2*, and *SLC7A1,* and others transporting key amino acids, increases upon T cell activation.[13,22] Expression of these transporters, sensing extracellular amino acids in a mechanistic target of rapamycin (mTOR)-dependent way, as well as their intracellular utilization, regulates CD4+Teff and Treg cells' metabolic reprogramming and subsequently their proliferation, differentiation, and function.[13,20,21,23–25] Amino acid metabolizing enzymes, such as interleukin 4 induced gene 1 (IL4I1)[26–29] or glutamate oxaloacetate transaminase 1 (GOT1),[30] also affect Teff and Treg cells. L-Phenylalanine (Phe), transported by the same transporters might be partly metabolized by the same enzymes, but its role in metabolism, proliferation, and differentiation of memory CD4+T cells has not been explored.

Allergic diseases are common type 2 inflammatory diseases, characterized by skewed Teff-Treg responses, where pathogenic Th2 cells cannot be efficiently suppressed by impaired Treg cells. In humans, pathogenic CRTH2+ or CD161+Th2 cells, overexpressing GATA3 and PPARγ and producing interleukin (IL)-4, IL-5, IL-9, and IL-13, are predominantly involved in the onset and maintenance phase.[31–35] Th2 cells require mTORC1 for engagement of glycolysis and cell-cycle entry,[36] mTORC2 for cell survival and migratory functions,[23,37] and fatty acid synthesis for lineage development.[38] We and others demonstrated that, in severe forms of allergic diseases, there are systemic and cellular alterations in amino acids, fatty acids, and glycolysis.[39–42] In patients with severe allergic asthma, many amino acids including arginine, phenylalanine, taurine, and xanthine are increased in plasma,[43] but how it affects metabolism and functions of memory Th2 and Treg cells remains elusive.

Here, we report that Phe constitutes a previously unknown checkpoint in the development, energy metabolism, and function of Th2 cells. We discovered that metabolome profiles of *ex vivo*-sorted circulating human memory CD4+Teff and Treg cells clearly differentiate these cell types and are enriched in Phe and Arg metabolites. Combining *in vitro* functional experiments, RNA sequencing (RNA-seq), and knockdown approaches in primary human CD4+T cells; memory CD4+T cells; and Th1, Th2, Th17, and Treg cells, with *ex vivo* single-cell energy metabolism profiling, we discovered that high level of intracellular Phe increases glycolysis but restricts OXPHOS, as well as affects memory CD4+T and Th2 cells proliferation in IL4I1-dependent manner. Phe also decreased phosphorylation of mTOR and STAT6 in Th2 cells, leading to the decrease in cell activation and type 2 cytokines production. Finally, multi-omics analysis of five independent allergic patient cohorts revealed that in severe allergy there is an impairment of SLC7A5-dependent transport of Phe into Th2 cells and an increase in its intracellular processing, leading to expansion of pathogenic Th2 cells.

RESULTS

Circulating human memory CD4+T effector and Treg cells have different metabolic profiles and are enriched in phenylalanine and arginine metabolites

We first aimed to assess the metabolome of circulating human memory T cells in an unbiased manner after *ex vivo* cell sorting. We focused on analyzing the memory T effector (Teff) and memory Treg cells by untargeted metabolomics and lipidomics (Figures 1A, 1B, and S1; Table S1). We obtained a total of 195 and 233 metabolites for Teff and Treg cells, respectively, of which 133 (45%) were shared (Figure 1C; Table S2). Principal-component analysis (PCA; Figure 1D) and orthogonal partial-least discriminant analysis (Figure S2A) models revealed a complete separation between these populations even though their metabolites belonged to similar families (Figure 1E). Carboxylic acids, which include major amino acids, such as phenylalanine (Phe) and arginine (Arg), represented the largest group in both cell types (Figure 1E). Fatty acyls, glycerophospholipids, and organooxygen compounds were the other major classes (Figure 1E; Table S3). The metabolomes of both cell subsets were enriched in metabolites related to pathways such as amino

(C) Venn diagram representing intracellular metabolites (n = 295) detected by untargeted mass spectrometry metabolomics and lipidomics in memory CD4+Teff (red) and Treg (blue) cells from healthy individuals.

(D) PCA model of memory CD4+Teff and Treg cells, based on all (shared and unique) metabolites. Data were logarithmic transformed and pareto scaled (log x Par).

(E) Pie charts representing biochemical composition of all (shared and unique) metabolites detected in Teff and Treg cells, ordered by abundance.

(F) Metabolic pathways analysis in memory CD4+Teff and Treg cells. Significant ($-\log_{10}$(p value) > 1.3) and corresponding pathways (n = 25) are shown for either Teff, Treg, or both. Over-representation analysis was performed by IMPaLA[44] including shared and unique metabolites. Pathways related to Phe and Arg metabolism are highlighted in bold.

(G) PCA model of memory CD4+Teff and Treg cells of shared metabolites only (n = 133). Data were log x Par.

(H) Heatmap (left) and bar graph of fold changes (Log_2FC) (right) of identified shared metabolites in memory CD4+Teff vs. Treg cells. Data were logarithmic transformed and unit variance scaled. *$p < 0.05$. Metabolites related to Phe and Arg metabolism are highlighted in bold.

(A–H) Analysis done in n = 6 different healthy donors. (E) Metabolites are organized in main biochemical classes according to Human Metabolome Database (HMDB v.2022).[45] Examples of metabolites are shown in major classes (>5%). See also Figures S1 and S2 and Tables S1, S2, S3, S4, S5, S6, and S7.

acids biosynthesis and metabolism, vitamin synthesis, and energy metabolism (Figure 1F). Among ten overrepresented amino-acid-related pathways, six were related to Phe and Arg metabolism (Figure 1F, bold). Moreover, memory Teff and Treg cells were uniquely enriched in glycerophospholipid metabolism and fatty acid biosynthesis, respectively. (Figure 1F; Tables S4 and S5).

Since the strongest over-representation in both cell types was present due to highly abundant shared metabolites, we focused primarily on these compounds. Interestingly, we found that shared metabolites can also clearly separate Teff cells from Treg cells (Figures 1G and S2B), suggesting different abundance of these metabolites. Regarding their biochemical classes, likewise, carboxylic acids, including many amino acids, organooxygen compounds, and fatty acyls, represented the biggest fractions (Figure S2C; Table S6). Among these, we found some organooxygen compounds, such as pantothenic acid, and glycerophospholipids, such as lysophosphatidic acid, to be significantly elevated in Teff cells, while fatty acyls like N-palmitoyl histidine//PGH2-EA and carboxylic acids, such as Arg, phenylpyruvic acid, histidine, and citrulline, were significantly higher in Treg cells (Figures 1H; Table S7). Interestingly, several of these differentially abundant metabolites are also linked to Phe and Arg metabolism (Figure 1H, bold).

L-Phenylalanine increases glycolysis and inhibits OXPHOS in CD4$^+$T cells

Since we observed enrichment in energy metabolism and Phe and Arg metabolism-related pathways in memory CD4$^+$Teff and Treg cells, we aimed to assess their effects on energy metabolism of CD4$^+$T cells at baseline and after activation. As expected, activated total CD4$^+$T cells displayed higher levels of inducible and compensatory glycolysis, and these parameters were further elevated after 72 h incubation with increasing concentrations of Arg (base medium contains 1.149 mM of Arg) (Figure 2A). We also observed increased levels of maximum respiratory capacity with higher concentrations of Arg (Figure 2B). Similarly, activated total CD4$^+$T cells incubated in additional 0.1 and 1 mM Phe (base medium contains 90.9 μM of Phe) for 72 h had higher inducible and compensatory glycolysis (Figure 2C). Interestingly, the maximum respiratory capacity of cells incubated in additional 1 mM of Phe was significantly lower compared to that of cells incubated in 0.1 mM Phe-supplemented media (Figure 2D) suggesting that elevated Phe negatively influences OXPHOS. When the same experimental setup was repeated in memory CD4$^+$T cells treated with Phe, we observed again increased inducible and compensatory glycolysis with increasing doses of Phe. Likewise, the maximum respiratory capacity was reduced in cells incubated in 1 mM Phe-supplemented media (Figure 2E).

To sum up, we observed that, while Arg induces both glycolysis and OXPHOS in human CD4$^+$T cells, Phe enhances glycolysis but impairs OXPHOS at higher doses in total and memory human CD4$^+$T cells.

L-Phenylalanine controls CD4$^+$T cell proliferation via induction of IL4I1 enzyme

Considering the ability of Phe to differentially influence glycolysis and OXPHOS in memory CD4$^+$T cells upon activation, we sought to determine if Phe also affects their proliferation. Memory CD4$^+$T cells incubated in full media (base medium contains 90.9 μM of Phe) supplemented additionally with 1 mM Phe showed reduced proliferation (Figures 3A and S3) with no difference in viability (Figure 3B). To gain insight into the involved mechanism, we assessed the mRNA expression of *IL4I1* that has been reported to limit effector T cell proliferation (total, memory, CD8$^+$T, CD4$^+$T, and Th17) and catalyzes the conversion of Phe to phenylpyruvate.[29,46,47] We found that supplementation of CD4$^+$T cells with high levels of Phe tended to upregulate mRNA expression of *IL4I1* (Figure 3C). To confirm that Phe-induced IL4I1 blocks memory CD4$^+$T cell proliferation, we carried out small interfering RNA (siRNA)-based knockdown. We observed significant knockdown of IL4I1 in these donors (Figure 3D). We found a significant increase in memory CD4$^+$T cells proliferation when IL4I1 was knocked down and cells were either activated (Figure 3E) (base medium contains 90.9 μM of Phe) or non-activated (Figure S4A), which was not decreased by additional Phe in IL4I1 knockdown. IL4I1 knockdown did not affect cell viability (Figures 3F and S4B). Next, we were curious if this effect was specific to certain T helper (Th) cell subsets. Hence, we differentiated human naive helper CD4$^+$T cells into Th1, Th2, Th17, and Treg cells (Figure S5; Table S8) and knocked down IL4I1 in these cells (Figure 3G). We observed that IL4I1 knockdown significantly increased Th2 cell and Th17 cell proliferation (Figure 3H).

In short, Phe in OXPHOS-impairing doses also limits proliferation of memory CD4$^+$T cells and of *in vitro*-differentiated Th2 and Th17 cells via an IL4I1-dependent mechanism.

L-Phenylalanine inhibits translation, induces glycolysis, and represses OXPHOS in activated human Th2 cells, analyzed on a single-cell level

Next, we studied the effect of Phe on energy metabolism of Th1, Th2, Th17, and Treg cells in *ex vivo*-isolated total CD4$^+$T cells and peripheral blood mononuclear cells (PBMCs) on a single-cell level. We employed single-cell energetic metabolism by profiling translation inhibition (SCENITH),[48] (Figure 4A; Tables S8 and S9). Activation-induced translation of Th1 cells was highest among the subsets and was not affected by Phe (Figure 4B). In contrast, Phe significantly decreased total translation in activated Th2 (Figure 4C). In Treg cells, Phe tended to limit total translation (Figure 4D). The SCENITH assessment of Th17 cells was unreliable due to the limited number of cells. Similarly, we did not see an effect of Phe on glycoATP and mitoATP production in *in vitro*-differentiated Th1, Th17, and Treg cells (Figures S6A–S6F). In PBMCs, assessed as a more physiological compartment, we included additional markers to further analyze Th2 and Treg cells in more detail (Figure S6G). Again, both subsets highly increased the level of translation upon activation (Figures 4E–4G). In Th2 cells, an increase in translation (Figure 4G), glucose dependence (Figure 4H), and glycolytic capacity (Figure 4I) was observed upon activation, paired with a significant decrease in mitochondrial dependence (Figure 4J) as well as FAO and amino acid oxidation (AAO) capacity (Figure 4K). Addition of Phe significantly increased glycolytic capacity of activated Th2 cells (Figure 4I) and decreased their mitochondrial dependence (Figure 4J), which was reflected in a decrease of translation

CellPress
OPEN ACCESS

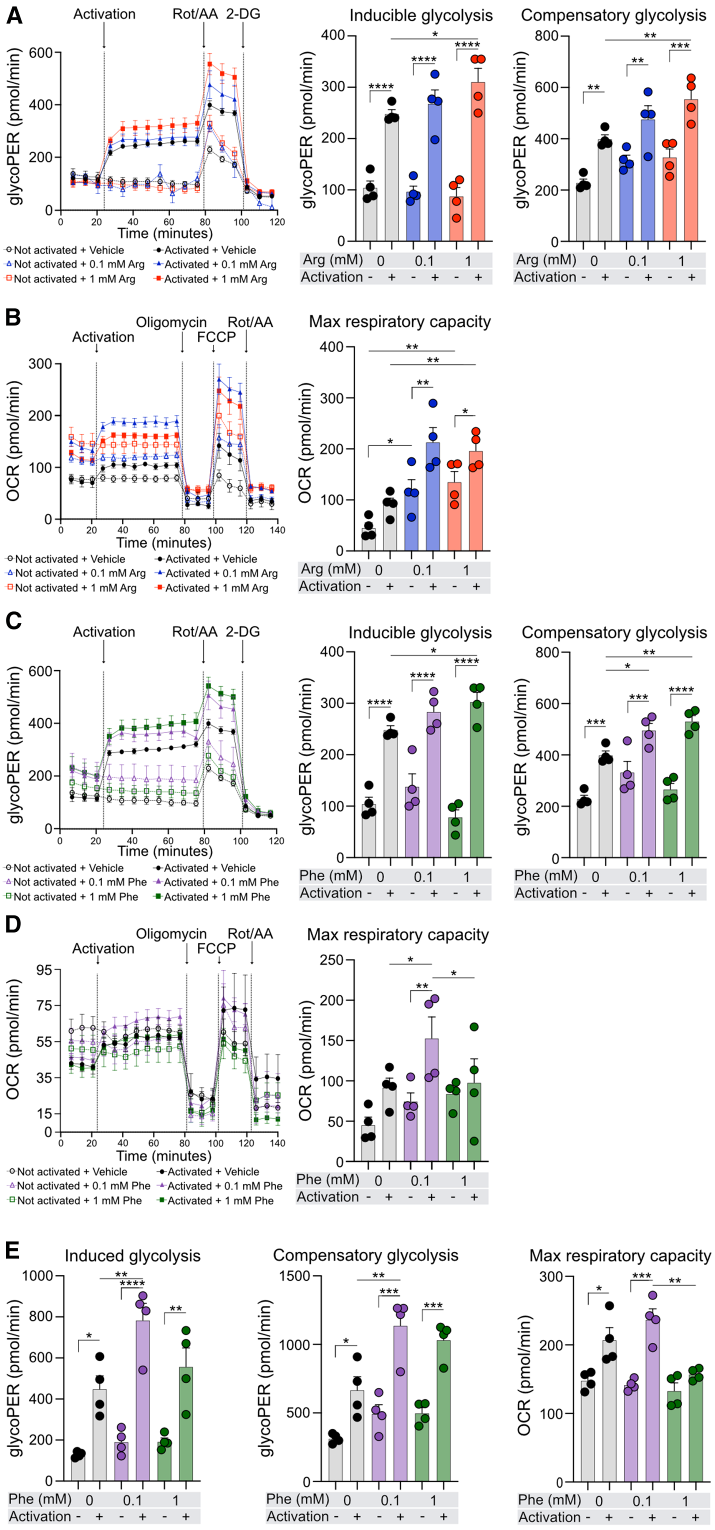

Figure 2. High level of L-phenylalanine enhances activation-induced glycolysis but inhibits OXPHOS, while arginine enhances activation-induced glycolysis and OXPHOS in human CD4$^+$T and memory CD4$^+$T cells

(A) Representative glycolytic proton efflux rate (glycoPER) graph of Seahorse glycolytic rate assay (left); quantification of inducible and compensatory glycolysis (right) of CD4$^+$T cells treated in full medium (containing 1.149 mM Arg) with additional 0.1 (blue) and 1 mM (red) Arg supplementation or vehicle (gray) for 72 h with/without acute CD2, CD3, and CD28 activation.

(B) Representative oxygen consumption rate (OCR) graph of Seahorse Mito Stress test (left); quantification of maximum respiratory capacity (right) of CD4$^+$T cells treated with Arg as in (A).

(C) Representative glycoPER graph of Seahorse glycolytic rate assay (left); quantification of inducible and compensatory glycolysis (right) of CD4$^+$T cells treated in full medium (containing 90.9 μM of Phe) additionally supplemented Phe at concentrations of 0.1 (violet) and 1 mM (green) or vehicle (gray) for 72 h, with/without acute CD2, CD3, and CD28 activation.

(D) Representative OCR graph of Seahorse Mito Stress test (left); quantification of maximum respiratory capacity (right) of CD4$^+$T cells treated with Phe as in (C).

(E) Quantification of induced glycolysis, compensatory glycolysis, and maximum respiratory capacity of memory CD4$^+$T cells treated with Phe as in (C) and (D).

(A–D) Data are representative of three independent experiments in three different donors or (E) in one donor. Data were analyzed by one-way ANOVA with Fisher LSD test. Bar graphs represent mean ± SEM. **p* < 0.05, ***p* < 0.01, ****p* < 0.001, and *****p* < 0.0001. Arg, L-arginine; Phe, L-phenylalanine; Rot/AA, rotenone/antimycin A; 2DG, 2-deoxyglucose; FCCP, carbonyl cyanide-*p*-trifluoromethoxyphenylhydrazone. All Seahorse measurements were normalized to total protein concentration.

(Figure 4G). In Treg cells, upon activation, we observed an increase in translation, which was lower than that in Th2 cells (Figures 4F and 4G). There was also an activation-induced increase in their glycolytic capacity (Figure 4M) and a reduction in mitochondrial dependence (Figure 4N) but no changes in glucose dependence (Figure 4L) or FAO and AAO capacity (Figure 4O). Interestingly, in Treg cells, Phe did not significantly affect any of the glycolytic or mitochondrial processes (Figures 4L–4O).

These data suggest that high intracellular levels of Phe repress the global ability of activated Th2 cells to produce ATP by inducing metabolic reprogramming toward increased glycolytic capacity and completely blocked mitochondrial dependence.

L-Phenylalanine inhibits Th2 cell proliferation; reduces mTOR and STAT6 phosphorylation; and inhibits Th2 transcription factors, cytokines, and activation markers

Following the effect of Phe on energy metabolism and proliferation of human CD4$^+$T cells, out of which Th2 was the most Phe-sensitive subset, we next studied in detail effect of Phe on *in vitro* highly/long differentiated human Th2 cells[49] (Figure S7). To confirm that extracellular Phe enters the cells, we measured intracellular Phe in activated Th2 cells incubated in Phe-supplemented media, observing its significant increase (Figure 5A). Similar to Phe-treated, SCENITH-assessed Th2 cells (Figure 4I), here, we observed increased levels of inducible glycoATP in activated *in vitro*-differentiated Th2 cells incubated in 1 mM of additional

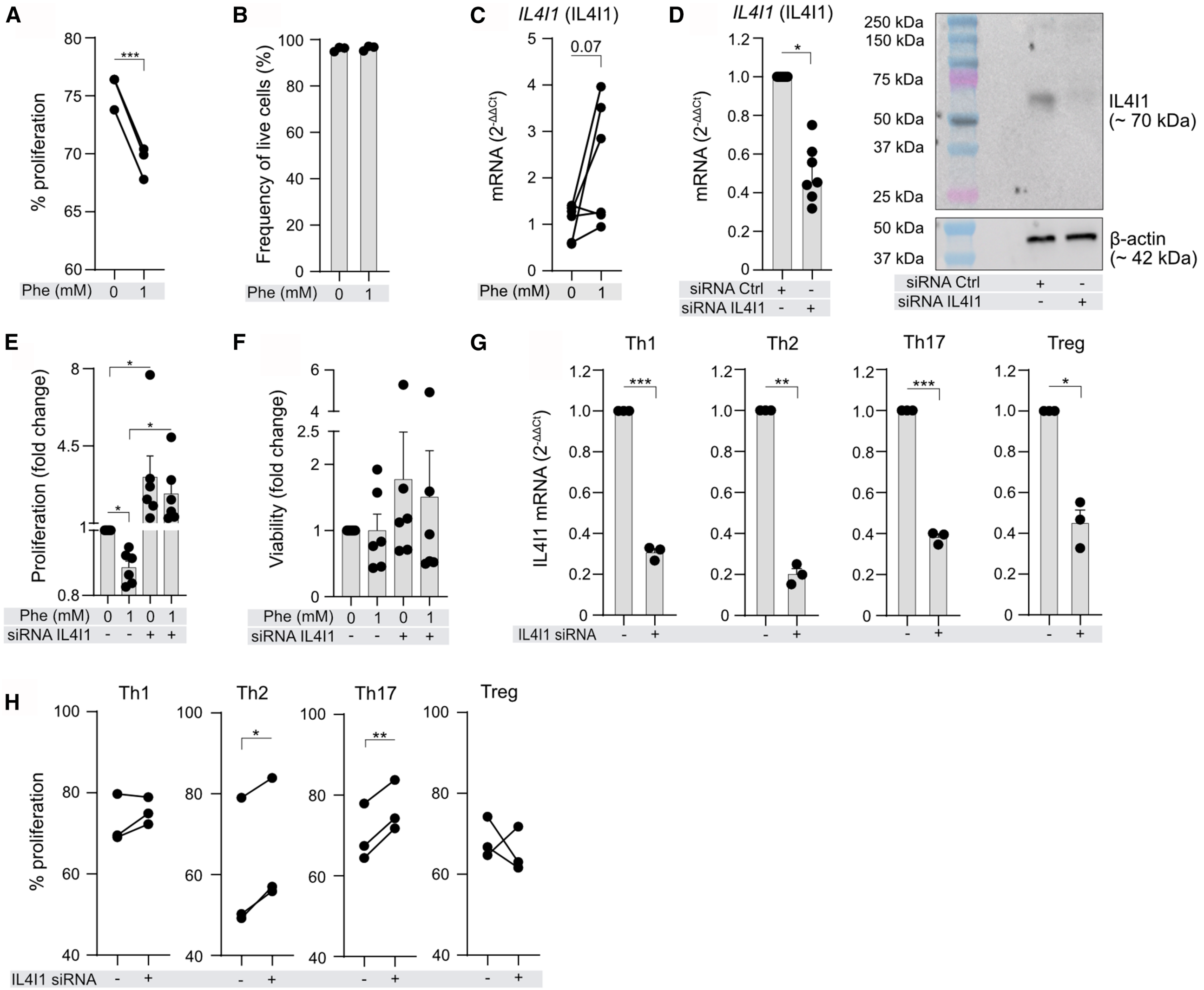

Figure 3. L-phenylalanine inhibits proliferation of human memory CD4$^+$T cells by induction of interleukin 4 induced gene 1 enzyme

(A) Proliferation of memory CD4$^+$T cells incubated in full medium (containing 90.9 μM of Phe) supplemented with vehicle or 1 mM Phe and activated with CD2, CD3, and CD28 antibody-coated beads for 72 h. n = 3 different subjects.

(B) Frequency of live memory CD4$^+$T cells in the same experiments as in (A).

(C) Expression of *IL4I1* mRNA in memory CD4$^+$T cells following similar treatment as in (A). Data from 6 independent experiments in 6 different subjects.

(D) *IL4I1* mRNA expression (left) and representative WB image of IL4I1 (right) in siRNA knockdown experiments in memory CD4$^+$T cells. Data show 3 independent experiments in 6 different subjects. One outlier was identified using Grubbs' test with α = 0.05. One donor was included in two experiments.

(E) Proliferation of control siRNA (Ctrl)- and IL4I1 siRNA-treated memory CD4$^+$T cells from 2 independent experiments in 5 different subjects. One donor was included in both experiments.

(F) Viability of control siRNA (Ctrl)- and IL4I1 siRNA-treated memory CD4$^+$T cells in the same experiments as in (E).

(G) Expression of *IL4I1* mRNA in *in vitro*-differentiated human Th1, Th2, Th17, and Treg cells in siRNA knockdown experiments following similar treatment as in (D) (n = 3 different donors).

(H) Proliferation of control siRNA (Ctrl)- and IL4I1 siRNA-treated Th1, Th2, Th17, and Treg cells, incubated in full medium (containing 90.9 μM of Phe) with 1 mM additional Phe and treated with CD2, CD3, and CD28 activation antibody-coated beads for 48 h before flow cytometry (n = 3 different donors).

(A–H) Each dot represents one donor. (E and F) Bar graph shows fold change as compared to activated vehicle-treated cells. Paired t test was used in (A), (B), (C), (G), and (H); Wilcoxon test was used in (D)–(F). All data are presented as mean ± SEM. *$p < 0.05$, **$p < 0.01$, and ***$p < 0.001$. See also Figures S3, S4, and S5 and Table S8.

Phe (Figure S8A). However, we did not note any major influence of Phe on mitoATP levels (Figure S8B), demonstrating superior sensitivity of SCENITH in assessing mitochondrial dependence. In contrast, additional Arg did not have any effect on *in vitro*-differentiated Th2 cell energy metabolism (Figures S8C and S8D). Supplementation with 1 mM of additional Phe significantly reduced proliferation of activated Th2 cells (Figure 5B) while increasing their viability (Figure 5C). In non-activated conditions, Phe increased proliferation of Th2 cells (Figure S9A), with no effect on their viability (Figure S9B). We observed an increase in the

CellPress
OPEN ACCESS

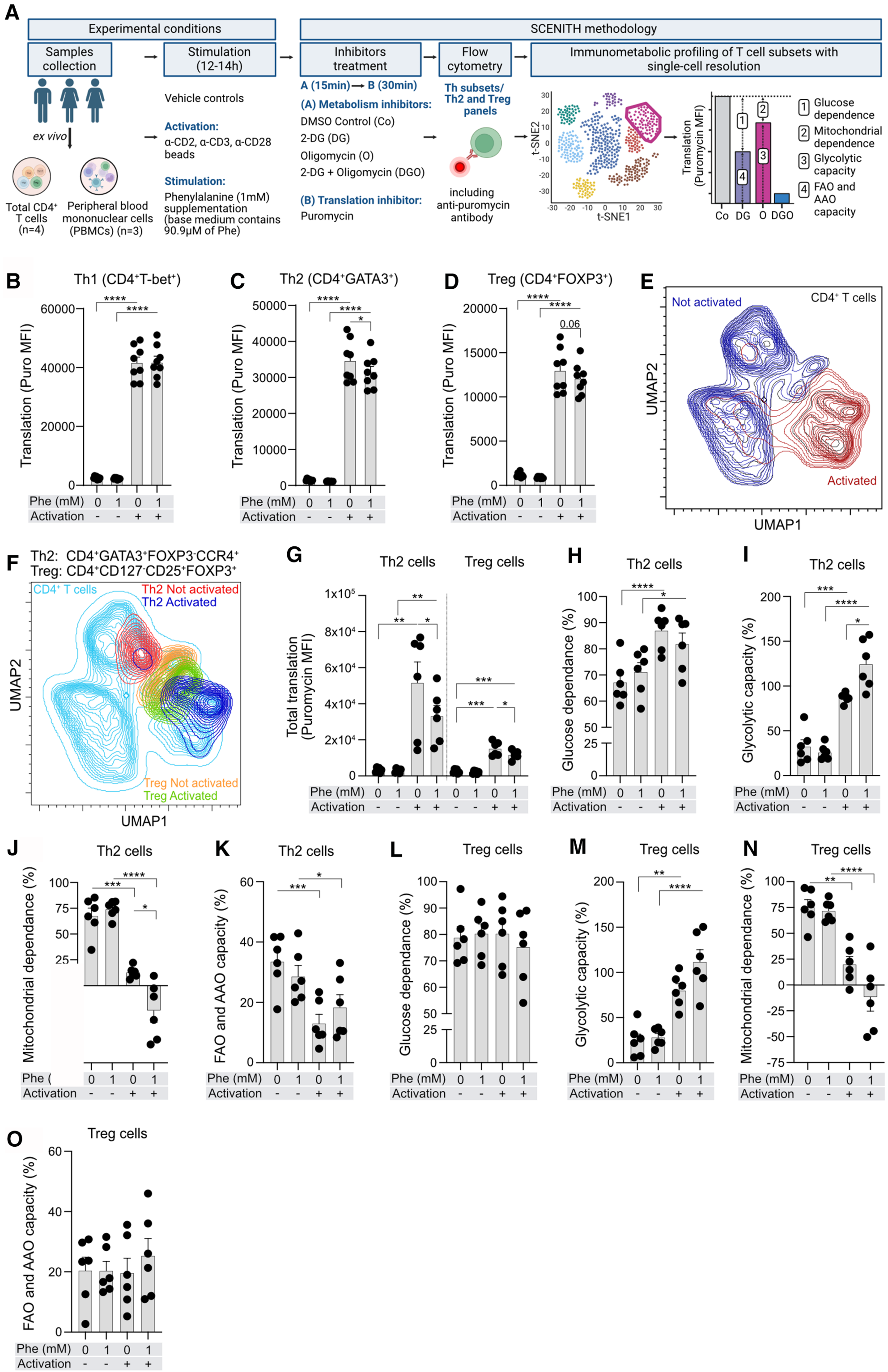

(legend on next page)

protein expression of IL4I1 (Figures 5D, 5E, and S9C), upon supplementation of 1 mM Phe suggesting IL4I1 involvement in limiting Th2 cell proliferation in agreement with our knockdown results (Figure 3H). To gain unbiased insights into the effect of Phe on Th2 cells, we conducted RNA sequencing. A total of 406 genes were significantly altered in activated 1 mM Phe-treated Th2 cells in comparison to Veh-treated activated cells (Figure S10; Table S10). Among these, we observed differential expression of several genes related to STAT6, mTOR, and AMP-activated protein kinase (AMPK) signaling pathways (Figure 5F), resulting in downregulation of cytokine and cell activation pathways (Figure 5G) and upregulation of lipid synthesis and glycolysis pathways (Figure 5H). Consequently, we observed that Phe significantly decreased STAT6 and mTOR phosphorylation within 30 and 60 min, respectively, after activation (Figures 5I, 5J, and S11A), which was followed by the decrease in mTOR protein (Figures 5K–5M and S11B). In agreement with RNA-seq data, we observed a gradual decrease in the mRNA expression of key type 2 cytokines such as *IL-4*, *IL-5*, and *IL-13* only upon treatment with increasing doses of Phe, but not Arg (Figures 5N and S12; Table S11). Similarly, mRNA levels of important type 2 transcription factors including *mTOR*, *RAPTOR*, *RICTOR*, *BACH2*, and *BATF* and activation markers such as *CD69* and *PTGDR2* were significantly reduced in Phe- but not Arg-treated activated Th2 cells (Figures 5N and S12). Expression of *GATA3*, *STAT6*, *IFNG*, *IL2RA*, and *PDCD1* was unchanged (Figure 5N). Phe also reduced frequency of IL4$^+$Th2 cells (Figure 5O). Finally, since it has been previously reported that CD161 is an important marker associated with pathogenicity of Th2 cells (so-called Th2a cells) in humans,[50] we assessed if CD161 expression is influenced by Phe. Indeed, we observed a significant reduction of CD3$^+$CD4$^+$CCR4$^+$GATA3$^+$ CD161$^+$Th2 cell frequency in activated conditions upon treatment with Phe (Figures 5P and S13).

In summary, in *in vitro*-differentiated human Th2 cells, Phe, but not Arg, affects T cell receptor (TCR) activation-induced glycolysis, proliferation, phosphorylation, transcription, and translation of key molecules involved in differentiation and function of Th2 cells, including markers of their pathogenicity.

Low intracellular levels of L-Phenylalanine in human memory CD4$^+$T effector cells characterize severe allergic patients with systemic type 2 inflammation

Having observed an influence of Phe on Th2 cells and knowing that patients with severe forms of type 2 diseases exhibit alterations in memory Teff cells and Treg cells,[51–53] we hypothesized that the metabolomes of these cells in allergy might reflect, to some extent, their changed functionality.

We recruited non-allergic, mild, and severe allergic individuals to olive pollen out of the pollen season (Cohort A) (Tables S12 and S13). Cohort A consisted of clinically well-described subjects at different stages of allergic sensitization and symptoms.[54,55] Severe allergic patients had higher number of pathogenic memory CRTH2$^+$CD161$^+$ Th2a cells[32,50] (Figure 6A), although, their total CRTH2$^+$Th2 and Treg cells were not significantly different (Figure S14A). Interestingly, they also had higher number of CRTH2$^+$Treg and PD1$^+$Treg cells (Figure 6B), which have been reported to have Th2-like phenotype and inefficient regulatory capacity.[56–58] In addition, severe allergic patients had high numbers of ILC2 and ILC3 (Figures 6C and S14B), in agreement with previous reports.[59–61] By performing targeted proteomics, we found several proteins to be differentially expressed in serum of severe allergic patients (Figures 6D; S15). IL-5 and proteins involved in cell proliferation, namely brother of CDO (BOC) and pappalysin-1 (PAPPA), were significantly elevated in severe allergic patients (Figure 6D). These patients also had lower concentration of Th1-related cytokines such as IL-12, colony-stimulating factor-1 (CSF-1), or granzyme H (GZMH) and Treg-associated hepatocyte growth factor (Figure S15).

Next, we sorted the circulating memory CD4$^+$Teff and Treg cells from a subset of Cohort A patients and compared them to non-atopic, non-allergic healthy controls and analyzed their metabolomes. Metabolomic profiling of memory Th2 cells was not technically possible due to the limited abundance of these cells. We did not find any significant differences between healthy and allergic patients either in Teff or in Treg cells metabolomes (Tables S14 and S15). However, we identified two groups of subjects, whose samples clustered together (Figure 6E). Most metabolites in group 1, consisting of 80% of severe allergy patients, were less abundant than those in group 2, consisting of 50% of severe patients. These findings suggest that there are two distinctive cellular metabolic phenotypes in both cell subsets. Interestingly, group 1 and 2 consisted of mostly the same subjects in both cell types potentially suggesting their metabolic profiles are linked. Among them, Teff cells from allergic patients in group 1 had significantly lower levels of Phe (Figure 6F), Arg, and Leu/Ile (Figure S16). To understand this grouping, amid memory CD4$^+$Teff cells heterogeneity, we performed unbiased multidimensional phenotyping. We identified seven different populations within Teff cells in allergic patients (Figure 6G) and eight in healthy controls (Figures S17A and

Figure 4. Single-cell energy metabolism profiling revealing that L-phenylalanine increases glycolytic capacity and decreases mitochondrial dependence in activated human Th2 cells

(A) Schematic explaining workflow of single-cell energetic metabolism by profiling translation inhibition (SCENITH).[48] Created with Biorender.com.

(B–D) Total translation in activated Th1 (B), Th2 (C), and Treg (D) cells assessed in MACS-isolated total CD4$^+$T cells. Each dot represents one technical replicate per donor (n = 4 donors, 2 replicates each).

(E and F) Uniform manifold approximation and projection (UMAP) contour plots demonstrating activated and non-activated CD4$^+$T (E) and Th2 and Treg (F) cells. FlowJo plugin UMAP (version 4.0.4) was used for analysis.

(G) Total translation in Th2 and Treg cells assessed in whole PBMCs. One-way ANOVA with Fisher's LSD test was used for analysis.

(H–O) (H–L) Glucose dependence (I and M), glycolytic capacity (J and N), mitochondrial dependence (J and N), and (K and O) FAO and AAO capacity in Th2 and Treg cells, respectively.

(G–O) Each dot represents one technical replicate per donor (n = 3 donors, 2 replicates each). (B–D) Paired t test was used for analysis. (H–O) For comparing dependencies and capacities, one-way ANOVA with Sidak's multiple comparison correction was used. Bars represent mean ± SEM. *$p < 0.05$, **$p < 0.01$, ***$p < 0.001$, and ****$p < 0.0001$. See also Figure S6 and Tables S8 and S9.

CellPress
OPEN ACCESS

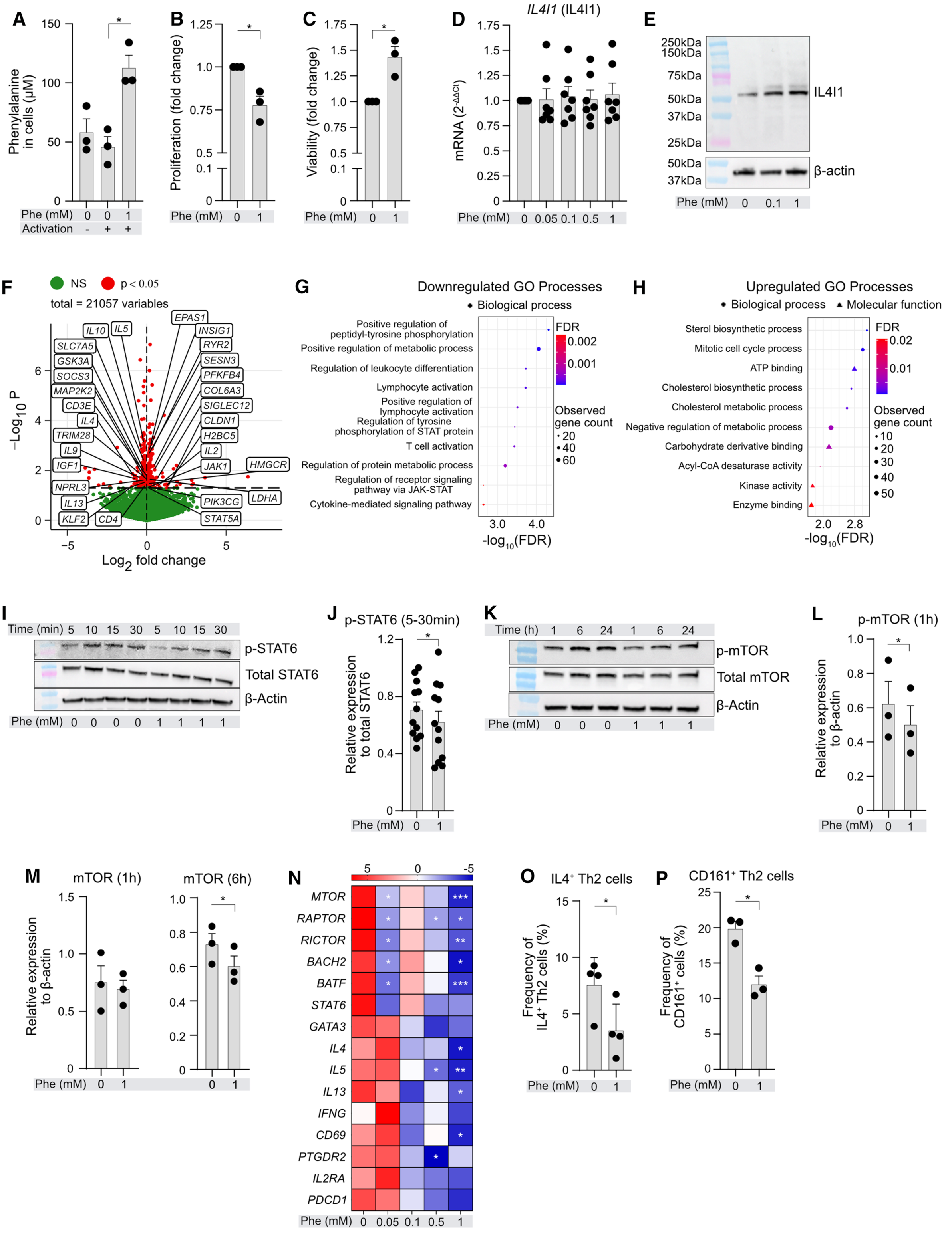

Figure 5. L-phenylalanine inhibits Th2 cell proliferation and mTOR and STAT6 phosphorylation as well as expression of type 2 transcription factors, cytokines, activation, and pathogenicity markers

(A) Phe uptake into Th2 cells. *In vitro*-differentiated Th2 cells from 3 different donors were incubated in indicated conditions for 6 h, and intracellular Phe was colorimetrically quantified in lysates.

(legend continued on next page)

S17B). As CRTH2 was expressed at low levels in sorting-activated cells,[62] and CRTH2$^+$ cells were both rare after down-sampling and heterogeneous, clustering was performed based on PD1 and CD161 expression. Notably, in allergic patients, we found strong negative correlations between levels of intracellular Phe and counts of CD161$^+$ populations (Figures 6H; Tables S16 and S17), but not CD161$^-$ populations (Figure S17C; Tables S16 and S17), which was not the case in healthy controls (Figure S17D; Tables S18 and S19). We observed that CD3$^+$CD4$^+$CD45RA$^-$CD127^{++}CD25$^-$CD161$^+$ cells (pool of populations 2, 3, 4, and 6; Figure 6H left), CD3$^+$CD4$^+$CD45RA$^-$CD127^{++}CD25$^-$CD161$^+$PD1$^+$ cells (pool of populations 3 and 4; Figure 6H center), and CD3$^+$CD4$^+$CD45RA$^-$CD127^{++}CD25$^-$CD161$^+$PD1$^-$ cells (pool of populations 2 and 6; Figure 6H right) correlated negatively with intracellular abundance of Phe.

In summary, severe allergy is associated with reduced intracellular phenylalanine in memory CD4$^+$ Teff cells, most notably in CD161$^+$Th2a enriched cells.

Extracellular L-phenylalanine decreases expression of large neutral amino acid transporters in Th2 cells *in vitro*, which is reflected *in vivo* in allergic patients

Prompted by these findings, we next explored expression of main molecules involved in Phe metabolism and transport in independent cohorts of patients with allergic diseases.

First, we analyzed Phe metabolism and transport in previously published RNA-seq data from Th2 cells sorted from patients with allergic asthma and allergic rhinitis or healthy controls (Cohort B).[63] We found that several of the top significant Gene Ontology (GO) processes enriched in Th2 cells were related with metabolic processes in patients with allergic asthma (Figure 7A; Table S20) and allergic rhinitis (Figure S18A; Table S21). There was an upregulation of amino acid metabolism (Figures 7B and S18B; Tables S22 and S23) and downregulation of L-threonine and Arg pathways and transport in allergic asthma (Figures 7C; Table S24) and allergic rhinitis patients (Figure S18C; Table S25), respectively. Several genes responsible for Phe metabolism and transport were present in these pathways (Table S26). We noted that *SLC7A5* (LAT1), *SLC7A8* (LAT2), and *SLC3A2* (CD98), part of LAT1 and LAT2 heterodimer complexes, were significantly downregulated in allergic asthma but not in allergic rhinitis (Figures 7D, 7E, and S18D), suggesting decreased transport of Phe into the Th2 cells in more advanced allergic disease. In addition, we noted that *GOT1* and *GOT2* were significantly upregulated in allergic asthma patients in Th2 cells (Figures 7D and 7E). Also, other genes encoding for enzymes indirectly involved in metabolism of Phe, such as pterin-4-alpha-carbinolamine dehydratase 2 (*PCBD2*), catechol-O-methyltransferase (*COMT*), and kynurenine aminotransferase 1 (*KYAT1*), were significantly upregulated, altogether suggesting potentially faster turnover of intracellular Phe in Th2 cells of patients with allergic asthma. We also analyzed gene expression of molecules belonging to Phe metabolism and amino acid transport in total CD3$^+$T cells in our own previously reported cohort (Cohort C)[64] consisting of healthy non-allergic controls and patients with mild and severe allergic asthma (GEO: GSE224253). We observed significantly reduced expression of multiple key genes involved in Phe transport and metabolism in total CD3$^+$T cells from severe, but not mild, allergy patients in comparison to controls (Figures S18E and S18F).

Next, to determine if serum levels of Phe were altered, we measured Phe concentration in a new, large cohort of subjects including healthy controls and mild and severe allergic patients enrolled in the same hospital and with the same clinical criteria as those from the Cohort A (Cohort D, Table S27). We observed that mild and severe allergic patients had higher levels of Phe in serum compared to non-allergic individuals (Figure 7F). To understand the relationship between the lower abundance of intracellular Phe and higher concentration of Phe in the serum of severe allergic patients, we analyzed an influence of extracellular Phe on gene expression of all parts of the LAT complexes. We found that increasing concentrations of extracellular Phe, but not Arg, significantly decreases mRNA expression of *SLC7A5* and *SLC7A8*, but not *SLC3A2*, in *in vitro*-cultured Th2 cells (Figures 7G and S19A–S19C). These observations were corroborated by our RNA-seq analysis wherein *SLC7A5* was significantly downregulated in Phe-treated activated Th2 cells (Figures 5F; Table S10). We confirmed this finding, demonstrating decreasing expression of LAT1 protein with increasing doses of Phe in the culture medium (Figures 7H and 7I). In addition, we confirmed that LAT1 is needed for the transport of Phe into the activated Th2 cells, since LAT1 inhibitor KYT0353

(B and C) Proliferation (B) and viability (C) of *in vitro* differentiated Th2 cells subjected to high doses of additional Phe. Bar graphs show fold changes compared to vehicle-treated, activated cells. $n = 3$ different donors.

(D and E) *IL4I1* mRNA expression (D) and representative WB image of IL4I1 protein expression (E) in *in vitro*-differentiated Th2 cells following incubation in increasing doses of Phe with/without concurrent activation. mRNA ($n = 6$–8 different donors) and protein expression ($n = 3$ different donors).

(F) Volcano plot of differentially expressed genes (DEGs, raw p value < 0.05) between activated Th2 cells treated with Phe (1mM) vs. vehicle for 24 h, obtained by RNA-seq analysis ($n = 5$ different donors). Genes related to STAT6/mTOR/AMPK signaling, critical for T cell activity, are highlighted in boxes.

(G and H) Significantly enriched downregulated (G) and upregulated (H) GO processes in Th2 cells following treatment as in (F). STRING analysis was conducted with significantly changed DEGs (raw p value < 0.05), and relevant enriched pathways are presented.

(I–M) Representative WB image (I and K) and quantification (J, L, and M) of phosphorylation of STAT6 and mTOR, respectively, in *in vitro*-differentiated Th2 cells ($n = 3$ different donors) treated with CD2, CD3, and CD28 activation antibodies with/without additional supplementation of 1 mM of Phe for indicated time points.

(N) Heatmap of mRNA expression of critical transcription factors, cytokines, and activation markers in activated *in vitro*-differentiated Th2 cells treated with increasing doses of Phe. mRNA expression was determined using RT-qPCR. $n = 6$–8 different donors. Data are analyzed using one-way ANOVA with Dunnett's correction. Z scores were determined and plotted as heatmap with different genes mentioned as rows. Data are row normalized.

(O) Frequency of activated IL4$^+$ Th2 cells with/without additional supplementation of 1 mM of Phe ($n = 4$ different donors).

(P) Frequency of activated CD3$^+$CD4$^+$CCR4$^+$GATA3$^+$CD161$^+$Th2 cells following incubation with/without supplementation of 1 mM Phe for 24 h ($n = 3$ different donors).

(A–D, J, L, M, N, O, and P) Each dot represents one donor. (A–C, J, L, M, O, and P) Paired t test was used for analysis. Bars represent mean ± SEM. *$p < 0.05$, **$p < 0.01$, and ***$p < 0.001$. See also Figures S7–S13 and Tables S9, S10, and S11.

CellPress
OPEN ACCESS

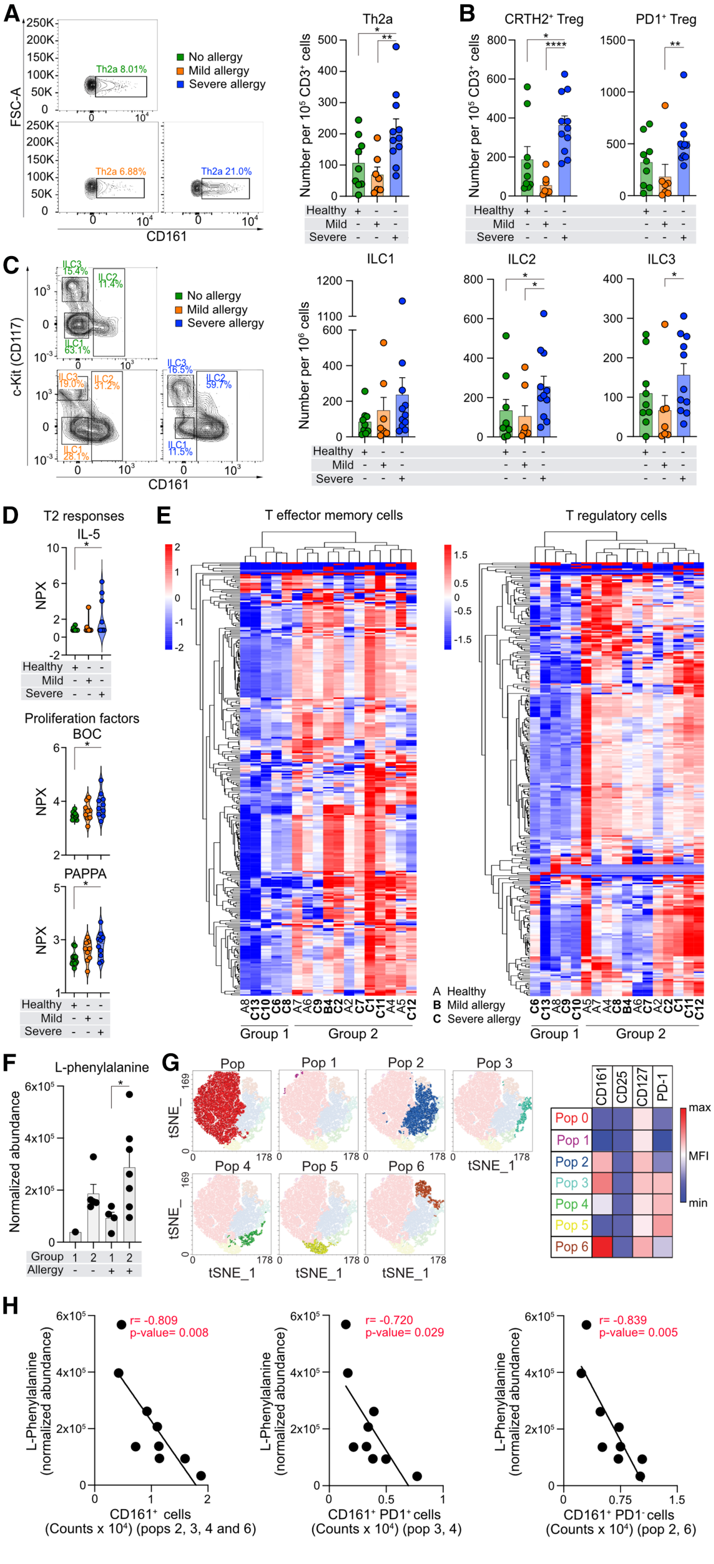

Figure 6. Low intracellular L-phenylalanine levels in pathogenic memory CD4$^+$T effector cell populations in severe allergic patients

(A–C) Representative flow cytometry dot plots of Th2a cells (A-left) and ILC1, ILC2, and ILC3 (C-left). Number of Th2a cells (A-right); memory CRTH2$^+$Treg cells and PD1$^+$Treg cells (B); and ILC1, ILC2, and ILC3 (C-right) in controls (n = 9) and patients with mild (n = 7) and severe (n = 11) allergy (Cohort A).

(D) Differentially expressed proteins in serum of controls (n = 10) and mild (n = 9) and severe (n = 10) allergic patients (Cohort A) assessed with PEA technology and presented as NPX.

(E) Heatmaps with hierarchical clustering analysis of all metabolites measured in memory CD4$^+$Teff (n = 195, left) and Treg (n = 233, right) cells in controls (n = 6) and allergic (n = 11) subjects. A, controls; B, mild allergy; C, severe allergy (from Cohort A).

(F) Normalized abundance of Phe in Teff cells in group 1 (control, n = 1; severe allergy, n = 4) and 2 (control, n = 5; mild allergy, n = 1, severe allergy, n = 6) (from Cohort A).

(G) t-distributed stochastic neighbor embedding (tSNE) plot of unbiased 2-dimensional flow cytometric analysis of memory CD4$^+$Teff cells (CD3$^+$CD4$^+$CD45RA$^-$CD127$^+$CD25$^-$) from patients with severe allergy (subset of cohort A, n = 9) identifying seven subpopulations based on CD161 and PD-1.

(H) Pearson correlation of normalized abundance of intracellular Phe, in memory CD4$^+$Teff cells from patients with severe allergy (n = 9), with counts of CD161$^+$ populations within memory CD4$^+$Teff cells (from Cohort A).

Mann-Whitney U test (A–C), one-way ANOVA with Fisher's LSD test (D), and unpaired t test (F) were used to compare differences among groups. Graphs represent mean ± SEM. *p < 0.05, **p < 0.01, and ***p < 0.001. Clinical characteristics of Cohort A are shown in Tables S12 and S13. NPX, normalized protein expression; Pop, population. See also Figures S14–S17 and Tables S1, S2, S12, S13, S14, S15, S16, S17, S18, and S19.

decreased uptake of Phe (Figure 7J). Finally, we measured LAT1 expression in CD4$^+$T cells and Phe levels in serum from patients with allergic asthma and healthy controls from our previous study[66] (Cohort E, Table S28). We found a negative correlation between serum Phe and LAT1 expression only in allergic asthma patients (Figure 7K).

In summary, severe allergy shows high serum Phe with LAT1 downregulation and increased Phe catabolism in Th2 cells, which likely reduces intracellular Phe and drives pathogenic Th2 expansion.

DISCUSSION

Metabolic specialization of human and mouse Teff subsets and Treg cells upon activation has been continuously demonstrated, showing higher dependence on glycolysis in Teff subsets and on FAO and OXPHOS in Treg cells,[6,9,67] although recent studies demonstrate that this metabolic reprogramming is tissue, nutrient, and context dependent.[10,11] In fact, T cells show metabolic plasticity depending on the environment and accessibility of nutrients. *In vivo*, when glucose level is much lower than in *in vitro* experiments, their energy metabolism, proliferative capacity, and effector response depend on other nutrients, such as amino acids or fatty acids.[12,20,68,69] Such experiments, however, have limited use in humans. Therefore, employing a combination of *ex vivo* cell sorting with high-resolution metabolomics and flow cytometry-based single-cell energy metabolism profiling

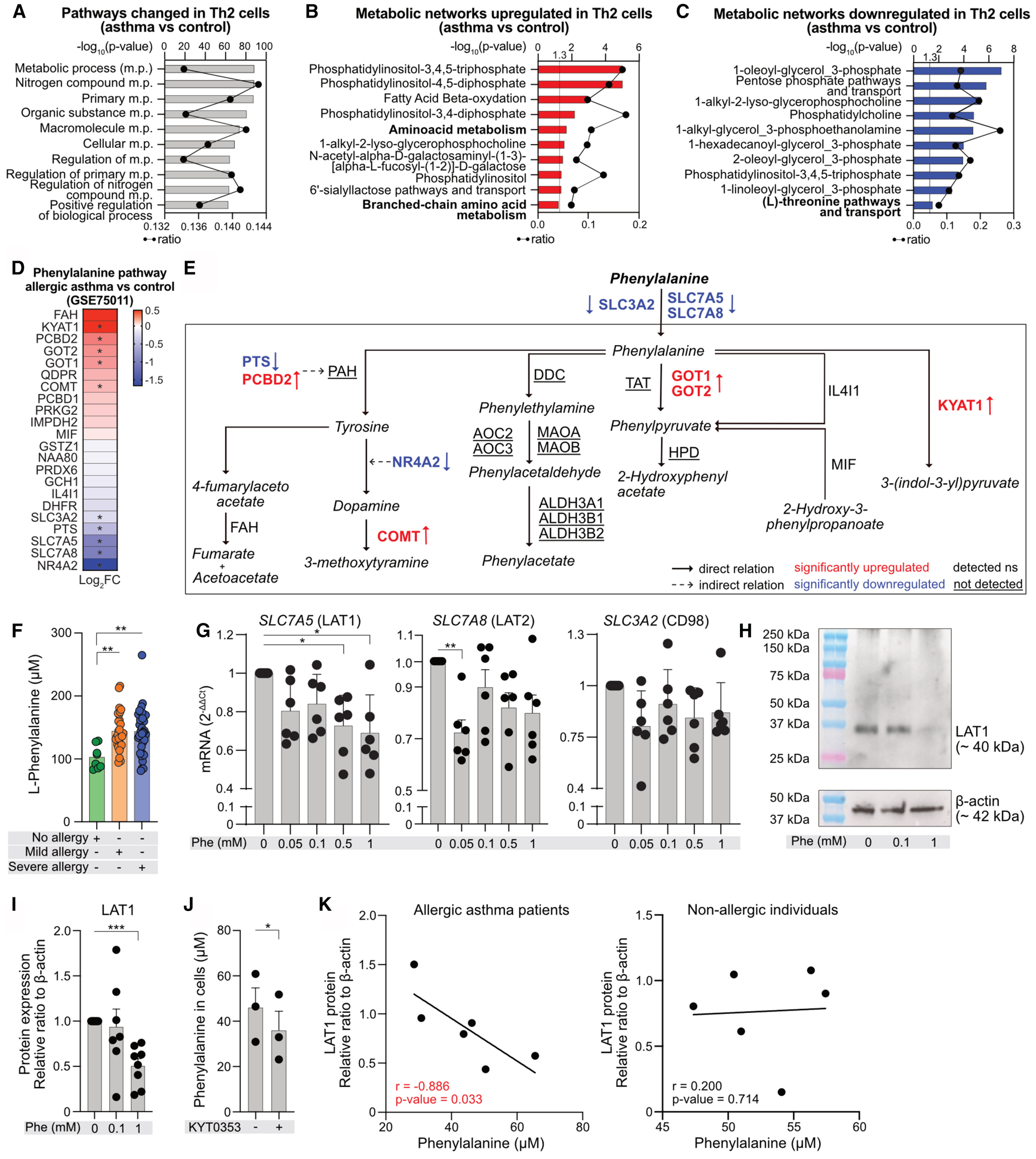

Figure 7. Decreased expression of large amino acid transporters in Th2 cells of allergic patients correlates with elevated serum levels of L-phenylalanine

(A–C) Top significantly enriched pathways (A) and upregulated (B) and downregulated (C) metabolic networks within differentially expressed genes (DEG, $p < 0.05$) in allergic asthma patients compared to controls (control $n = 15$, allergic asthma $n = 37$) from GEO: GSE75011 (Cohort B).[63] Black line represents ratio of genes in experiment over complete pathway set.

(D) Phe metabolism and transport pathway heatmap showing fold change (Log_2FC) of DEGs in allergic asthma patients ($n = 37$) compared to controls ($n = 15$) from GEO: GSE75011 (Cohort B).[63] *$p < 0.05$. Pathway curated and adapted from GSEA and MSigDB Database (Table S26).

(legend continued on next page)

methods has a potential to explore the metabolism of human T cells. Hence, we first utilized *ex vivo* cell sorting and metabolomics to study circulating human memory CD4$^+$T cell compartment, focusing on the previously unexplored comparison between human memory Teff and Treg cells. Our finding that metabolomes of these cells at the steady state differ substantially is quite striking. This adds to the previous findings showing that metabolome of *ex vivo*-purified human naive CD4$^+$T cells differs from metabolome of naturally occurring CD4$^+$CD25$^+$Treg[10] or thymus-derived Treg cells.[70] We found enrichment in various amino acids in both cell types. The strongest enrichment was observed in the phenylalanine and arginine metabolic pathways. In each cell type, however, particular amino acids might be used by different metabolic pathway,[19] considering their significantly different abundance. This is in line with previous observations of differential enrichment in amino acids in Th1, Th17, and T follicular helper cells.[71] Likewise, we observed a reduction in Phe- and Arg-related metabolites in CD4$^+$Teff compared to Treg cells, suggesting their faster utilization.[20] We also confirmed in the single-cell energy profiling experiments that *ex vivo*-activated Th1 and Th2 cells tended to have higher translation-used ATP production than Treg cells, suggesting differential anabolic and catabolic usage of available amino acids in both cell types.

Amino acids guide cellular functions through their selective sensing and acquisition.[19] Here, we showed that supplementation of Arg during activation increases glycolysis and maximum respiratory capacity in freshly sorted human CD4$^+$T cells but does not change glycoATP or mitoATP production in *in vitro*-differentiated human Th2 cells. Arg is transported in T cells via cationic amino acid transporter CAT-1 (SLC7A1).[20,72] Interestingly, in naive human CD4$^+$T cells upon activation, in the conditions of very high extracellular Arg supply (4 mM), Arg is quickly metabolized, feeding gluconeogenesis, limiting glucose uptake, and increasing OXPHOS.[20] This, in turn, limits differentiation but increases survival and maintains them in the memory like state.[20] In contrast, Phe, transported via LAT1 or LAT2,[73,74] in the same concentrations increased activation-induced glycolysis in total and memory human CD4$^+$T cells, glycoATP production in *in vitro*-differentiated Th2 cells, and glycolytic capacity in single-cell-assessed Th2 cells. It underlines Phe-induced glycolysis importance at different stages of CD4$^+$T cells differentiation. In highest concentrations, however, in total and memory CD4$^+$T cells Phe blocked activation-induced OXPHOS and downregulated mitochondrial dependence in *ex vivo*-treated Th2 cells. This observation is interesting, especially since we noted that the same concentrations of Phe limited memory CD4$^+$T cell and Th2 cell proliferation and type 2 cytokine production, while increasing their viability. It all suggests that during recall responses Phe might regulate the human memory Th2 cells by shifting its metabolites between OXPHOS and glycolysis.

In human T cells, phenylalanine hydroxylase, the main enzyme in Phe metabolism, is not expressed.[63,75] We found, however, that they express other enzymes metabolizing Phe. GOT1 and GOT2,[76] as well as IL4I1,[77] can catalyze conversion of Phe to phenylpyruvate. We also found phenylpyruvate in the memory Teff and Treg cells, as well as upregulated expression of GOT1 and GOT2 in Th2 cells of allergic asthma patients, suggesting that they might be involved in faster turnover of Phe driving pathogenic Th2 cell activity. IL4I1 can catalyze the conversion of Phe to phenylpyruvate liberating H_2O_2 and ammonia,[46] as well as can convert tryptophan to indoles and kynurenic acid activating aryl hydrocarbon receptor (AHR).[29] In CD8$^+$T and CD4$^+$T cells, external IL4I1 or intracellular upregulation of IL4I1 can limit their proliferation via temporary downregulation of TCR complex.[29,46,47] In CD4$^+$T cells, along with inhibition of proliferation,[46] external IL4I1 also facilitates differentiation of naive CD4$^+$T cells into Treg cells.[28] Here, we found that high levels of Phe increased expression of *IL4I1* in memory CD4$^+$T cells and in Th2 cells and, in agreement with previous findings[47] that genetic inhibition of IL4I1 increased proliferation of memory CD4$^+$T cells and, among CD4$^+$Teff cells, Th2 and Th17 cells were most affected.

Amino-acid-induced Raptor-mTORC1 activity is required in CD4$^+$T cells to regulate TCR-induced glycolysis, lipid biosynthesis, and OXPHOS, needed to exit from quiescence and initiate differentiation into various T helper subsets.[36] Lack of Raptor decreases IL-4 production by Th2 cells *in vitro* and diminishes allergic airway inflammation *in vivo*.[36] Also, Rictor-mTORC2 pathway is important for Th2 cells reprogramming.[23,37] Amino acids also regulate Treg function via licensing TCR-induced mTORC1 activation.[78] Accordingly, we noted an over-representation of mTOR pathway in metabolomes of human *ex vivo* memory CD4$^+$Teff and in Treg cells, paired with the enrichment of many amino acid pathways. In addition, we found that increased concentrations of Phe, but not Arg, upon TCR activation decreased phosphorylation of mTOR[23,79] and STAT6[80] and expression of MTOR, *RPTOR*, and *RICTOR* essential for their function, under established Th2 conditions, which

(E) Phe metabolism and transport schematic highlighting DEGs in Th2 cells of allergic asthma and controls (GEO: GSE75011) (Cohort B).[63] Significantly upregulated (red) and downregulated (blue) genes; detected but not significantly different (black); not detected in original dataset (black and underlined). Adapted from KEGG pathway.

(F) Serum Phe concentration in controls (n = 8) and mild (n = 30) and severe (n = 37) allergic patients (Cohort D) quantified by targeted metabolomics.[65] Kruskal-Wallis test was used for analysis.

(G) *SLC7A5* (LAT1), *SLC7A8* (LAT2), and *SLC3A2* (CD98; LAT3) mRNA expression in *in vitro*-differentiated Th2 cells treated with additional Phe with/without CD2, CD3, and CD28 activation for 24 h. Following incubation, mRNA expression was determined using RT-qPCR (n = 6–8 different donors).

(H and I) Representative WB image of LAT1 expression (H) and LAT1 protein quantification in 8 different donors (I) in *in vitro*-differentiated Th2 cells treated with additional Phe with/without CD2, CD3, and CD28 activation for 24 h. Expression of LAT1 presented as relative ratio normalized to β-actin.

(J) Phe uptake into *in vitro*-differentiated Th2 cells was quantified colorimetrically. Cells were incubated in full medium with/without SLC7A5 inhibitor (KYT0353) and activation of CD2, CD3, and CD28 for 6 h. Data were analyzed using paired t test (n = 3 different donors).

(K) Spearman correlation of serum Phe concentration and relative LAT1 expression in CD4$^+$T cells in allergic asthma patients (left) and controls (right) (Cohort E).

(G–I) Data are analyzed by one-way ANOVA with Dunnett's correction. (F–J) Bars represent mean ± SEM. Each dot represents one donor. *$p < 0.05$, **$p < 0.01$, and ***$p < 0.001$. See also Figures S18 and S19 and Tables S20, S21, S22, S23, S24, S25, S26, S27, and S28.

was coupled with decreases in expression of all type 2 cytokines, *IL4*, *IL5*, and *IL13*, surface activation marker *CD69*, and Th2-pathogenicity markers such as CD161 protein. Since these phenomena were coupled with an induction of IL4I1 pathway by Phe, they might result from the negative impact of IL4I1 on mTORC1 signaling,[28] potentially via activation of AHR.[29,47] Additionally, high doses of Phe also increased glycolysis and survival in Th2 cells *in vitro*, as well as increased glycolytic capacity, but decreased OXPHOS and translation in *ex vivo*-studied human Th2 cells, suggesting activation of AMPK signaling. Accordingly, we observed an increase in mRNA expression of *IL2*, 6-phosphofructo-2-kinase/fructose-2,6-biphosphatase 4 (*PFKFB4*), and lactate dehydrogenase A (*LDHA*), in RNA-seq analysis, which are critical factors facilitating survival and glycolysis in T cells,[81] as well as increase of 3-hydroxy-3-methylglutaryl-CoA reductase (*HMGCR*), and insulin-induced gene 1 (*INSIG1*), but downregulation of insulin-like growth factor (*IGF1*), further supporting activation of AMPK and subsequent repression of mTORC2.[82] It all suggests that high intracellular concentration of Phe present during TCR activation of Th2 cells reduced mTORC1, mTORC2, and STAT6 signaling via IL4I1 and reduced OXPHOS and/or AMPK activation. This leads to decreased proliferation, transcription, and translation of type 2 cytokines alongside of enhanced glycolysis and survival.

The increase in amino acid demand following T cell activation is satisfied via the upregulation of transporters among which heterodimers SLC7A5/SLC3A2 (LAT1) or SLC7A8/SLC3A2 (LAT2) mediate transport of large neutral amino acids, including Phe.[13] Without SLC7A5,[22] naive T cells are unable to proliferate, differentiate, or function properly, displaying profound defects in metabolic reprogramming.[13] Here, by analyzing different cohorts of allergic patients, we observed a reduction of LAT1 and LAT2 in circulating Th2 cells. It might lead to a decrease in intracellular concentration of Phe in memory Th2a subpopulations, which we observed in the most severe cases, paired with an increase of circulating type 2 inflammatory cells (Th2a, CRTH2$^+$Treg cells, and ILC2) and type 2 cytokines. This decrease in LAT1 expression on Th2a cells might result from the increased concentration of Phe in the plasma, as we noted here in patients from different groups and confirmed its causality *in vitro*. The physiological concentration of Phe in sera of healthy subjects has been reported to be between 16 and 166 μM[83] with daily variations of 50%[84] and in phenylketonuria (PKU) can be greater than 1.2 mM.[85] However, increased systemic levels of Phe and other amino acids in allergic patients[43,86] or in animal models of allergic inflammation and helminth infection have been reported previously.[87,88] Increased incidence of asthma and other type 2 diseases has also been reported in patients with PKU.[89] Decrease in LAT1 expression in circulating Th2 cells at the beginning of allergic disease may serve as a mechanism preventing excessive activation of Th2 cells, regulating the influx of several amino acids, thus limiting local Th2 and ILC2 cell activation.[87,88] However, in more severe allergic disease, when it is paired with overexpression of other enzymes metabolizing Phe, it leads to a decrease of intracellular Phe levels in Th2 cells, which cannot then block OXPHOS, activate IL4I1, or inhibit proliferation, which results in unleashed expansion of pathogenic Th2a cells.

Limitations and future directions

This study highlights the pleiotropic effects of Phe metabolism on proliferation, metabolic reprogramming, and function of human memory CD4$^+$T cell and Th2 cells and provides description of its impairment in severe allergic diseases in humans. However, it does not deliver mechanistic *in vivo* data in animal models of allergic airway inflammation. Although circulating memory CD4$^+$T cells often reflect tissue inflammation,[90–92] metabolic assessment of nasal or lung tissue-resident memory CD4$^+$T cells[93] and their subsets in *in vivo* and *ex vivo* models would be important to better understand immunometabolic dependencies in the tissue. Our metabolomics, proteomics, RNA-seq, and flow cytometry analyses, while conducted on relatively small cohorts of allergic patients and healthy controls, are mutually validated across each of all studied groups, underscoring the robustness and reliability of our findings. Nonetheless, studies in larger patient cohorts of different disease severity should follow to further explore the observed phenomena. Moreover, similar studies in other diseases such as cancer and diabetes would be beneficial.

RESOURCE AVAILABILITY

Lead contact

Further information and requests for resources and reagents should be directed to and will be fulfilled by the lead contact, Milena Sokolowska (milena.sokolowska@siaf.uzh.ch).

Materials availability

No new unique reagents were generated in this study.

Data and code availability

- Metabolomics data have been deposited in National Metabolomics Data Repository (NMDR)/Metabolomics workbench[94] repository with the accession number NMDR: ST003135 and are publicly available as of the date of publication. Transcriptomics data have been deposited in GEO with the accession number GEO: GSE291310. PEA proteomics data have been deposited in Mendeley Data (doi: https://doi.org/10.17632/9b64psp627.1) and are available as of the date of publication. Accession numbers are also listed in the key resources table.
- No original code was generated in the study.
- Any additional information is available from the lead contact upon request.

ACKNOWLEDGMENTS

This work was funded by the Swiss National Science Foundation (SNSF) grants (nr 310030_189334/1 and 320030-236264) to M.S. and ISCIII (project number PI24/00992) and co-funded by FEDER for the RICORS “Red de Enfermedades Inflamatorias (REI)” (RD24/0007/0018) and by Fundación Mutua Madrileña (project number: AP17023). J.R.-C. was supported by EAACI, CEINDO-BANCO SANTANDER, and UZH SEMP fellowships. A.V. acknowledges “Programa Ayudas Puente 2024-2025” from San Pablo CEU University Foundation and grant PEJ-2023-AI/SAL-GL-27622 from the Community of Madrid. J.R.-C., N.C., J.S.-S., and C.P.-T. were supported by predoctoral FPIs from Universidad CEU San Pablo. A.J.G.-C. was supported by grant PEJ-2023-AI/SAL-GL-27622. C.G.-C. was supported by a contract “Atracción de talento investigador” from Community of Madrid, Spain (2016-T2/BMD-1838). B.R.-L. acknowledges project PI22/01737, funded by ISCIII and the European Union.

AUTHOR CONTRIBUTIONS

Conceptualization: A.J.K., J.R.-C., V.S.S., D.B., A.V., and M.S. Methodology and investigation: A.J.K., J.R.-C., N.S., U.R., A.J.G.-C., M.I.D.D., N.C., I.J.P.,

CellPress
OPEN ACCESS

V.S.S., P.S., E.I., C.G.-C., J.S.-S., C.P.-T., C.M.-A., M.L.E., D.O., J.K., K.B., A.H., B.R.-L., C.A.A., R.J.A., A.V., and M.S. Data analysis and visualization: A.J.K., J.R.-C., N.S., U.R., A.J.G.-C., M.I.D.D., N.C., I.J.P., C.G.-C., A.E., G.T., D.Z., A.V., and M.S. Writing – original draft: A.J.K. and M.S. Writing – review: all authors. Supervision: C.G.-C., M.M.E., D.B., A.V., and M.S. Funding acquisition: J.R.-C., B.R.-L., M.M.E., D.B., A.V., and M.S. Project administration: D.B., A.V., and M.S.

DECLARATION OF INTERESTS

J.R.-C. is currently an employee of Thermo Fisher Scientific.

A.E. reports grants from Polish Medical Research Agency (ABM), and National Science Centre (NCN), during the conduct of the study and is a Board member of the Section of Medical Sciences at the Polish Academy of Science Olsztyn and Bialystok, EAACI Basic and Clinical Immunology Section Secretary, EAACI Task Force Chair, Polish Society for Experimental and Clinical Immunology - Board Member (Bialystok Section), and Scientific Board Member of the FlowPol Foundation.

C.A.A. has received research grants from the Swiss National Science Foundation, European Union (EU CURE, EU Syn-Air-G), Novartis Research Institutes (Basel, Switzerland), Stanford University (Redwood City, Calif), Seed Health (Boston, USA), and SciBase (Stockholm, Sweden); is the Co-Chair for EAACI Guidelines on Environmental Science in Allergic diseases and Asthma; Chair of the EAACI Epithelial Cell Biology Working Group; is on the Advisory Boards of Sanofi/Regeneron (Bern, Switzerland, New York, USA), Stanford University Sean Parker Asthma Allergy Center (CA, USA), Novartis (Basel, Switzerland), Glaxo Smith Kline (Zurich, Switzerland), Bristol-Myers Squibb (New York, USA), Seed Health (Boston, USA), and SciBase (Stockholm, Sweden); and is the editor-in-chief of *Allergy*.

R.J.A. is a tenured CNRS researcher at the Centre d'Immunologie de Marseille-Luminy and scientific advisor/co-founder of the start-up GammaOmics, which holds the exclusive license for SCENITH technology, which was used in this manuscript. This role is non-remunerated at present, but it is declared here for transparency.

M.S. has received research grants from the Swiss National Science Foundation (SNSF nr 310030_189334/1, nr 320030E_224154, and nr 320030-236264), GSK, Novartis, Stiftung vorm. Bündner Heilstätte Arosa, CK-Care, and OM Pharma and consultation fee from Roche, as well as speaker's fee from AstraZeneca and is an EAACI Executive Committee Member-at-Large.

STAR★METHODS

Detailed methods are provided in the online version of this paper and include the following:

- KEY RESOURCES TABLE
- EXPERIMENTAL MODELS AND STUDY PARTICIPANT DETAILS
  - Study participants recruitment
  - Buffy coats and human primary CD4$^+$T cell subsets
  - *In vitro* short differentiation of helper T cell subsets
  - *In vitro* long differentiation of human Th2 cells
- METHOD DETAILS
  - Isolation of peripheral blood mononuclear cells (PBMCs)
  - Flow cytometry and fluorescence-activated cell sorting (FACS)
  - Flow cytometry data analyses
  - Metabolomics
  - Analysis by RP-UHPLC-ESI-QTOF-MS analysis
  - Data processing
  - Workflow for lipid annotation
  - Targeted analysis of phenylalanine
  - Serum Protein Extension Assay (PEA) proteomics and targeted immunoassays
  - Seahorse flux analyses
  - Proliferation assay
  - Quantitative reverse transcription-polymerase chain reaction (RT-qPCR)
  - IL4I1 knockdown in human memory CD4$^+$T cells and proliferation
  - IL4I1 knockdown in human *in vitro* short-differentiated T helper cell subsets and proliferation
  - SCENITH: Single cell ENergetIc metabolism by profilIng translation inHibition
  - RNA sequencing sample preparation and analysis
  - RNA-seq and microarray data analysis of previously published datasets
  - Western blotting
  - Phenylalanine quantification in *in vitro* experiments
- QUANTIFICATION AND STATISTICAL ANALYSIS
  - Statistical analysis
  - Correlation analyses

SUPPLEMENTAL INFORMATION

Supplemental information can be found online at https://doi.org/10.1016/j.xcrm.2025.102466.

Received: July 2, 2024
Revised: May 7, 2025
Accepted: October 29, 2025
Published: November 26, 2025

REFERENCES

1. Kunzli, M., and Masopust, D. (2023). CD4(+) T cell memory. Nat. Immunol. *24*, 903–914. https://doi.org/10.1038/s41590-023-01510-4.
2. Sakaguchi, S., Yamaguchi, T., Nomura, T., and Ono, M. (2008). Regulatory T cells and immune tolerance. Cell *133*, 775–787. https://doi.org/10.1016/j.cell.2008.05.009.
3. Lee, W., and Lee, G.R. (2018). Transcriptional regulation and development of regulatory T cells. Exp. Mol. Med. *50*, e456. https://doi.org/10.1038/emm.2017.313.
4. Sallusto, F., Lanzavecchia, A., Araki, K., and Ahmed, R. (2010). From vaccines to memory and back. Immunity *33*, 451–463. https://doi.org/10.1016/j.immuni.2010.10.008.
5. Klicznik, M.M., Morawski, P.A., Hollbacher, B., Varkhande, S.R., Motley, S.J., Kuri-Cervantes, L., Goodwin, E., Rosenblum, M.D., Long, S.A., Brachtl, G., et al. (2019). Human CD4(+)CD103(+) cutaneous resident memory T cells are found in the circulation of healthy individuals. Sci. Immunol. *4*, eaav8995. https://doi.org/10.1126/sciimmunol.aav8995.
6. Jones, N., Vincent, E.E., Cronin, J.G., Panetti, S., Chambers, M., Holm, S.R., Owens, S.E., Francis, N.J., Finlay, D.K., and Thornton, C.A. (2019). Akt and STAT5 mediate naive human CD4+ T-cell early metabolic response to TCR stimulation. Nat. Commun. *10*, 2042. https://doi.org/10.1038/s41467-019-10023-4.
7. Michalek, R.D., Gerriets, V.A., Jacobs, S.R., Macintyre, A.N., MacIver, N.J., Mason, E.F., Sullivan, S.A., Nichols, A.G., and Rathmell, J.C. (2011). Cutting edge: distinct glycolytic and lipid oxidative metabolic programs are essential for effector and regulatory CD4+ T cell subsets. J. Immunol. *186*, 3299–3303. https://doi.org/10.4049/jimmunol.1003613.
8. Gerriets, V.A., Kishton, R.J., Johnson, M.O., Cohen, S., Siska, P.J., Nichols, A.G., Warmoes, M.O., de Cubas, A.A., MacIver, N.J., Locasale, J.W., et al. (2016). Foxp3 and Toll-like receptor signaling balance T(reg) cell anabolic metabolism for suppression. Nat. Immunol. *17*, 1459–1466. https://doi.org/10.1038/ni.3577.
9. Angelin, A., Gil-de-Gomez, L., Dahiya, S., Jiao, J., Guo, L., Levine, M.H., Wang, Z., Quinn, W.J., Kopinski, P.K., Wang, L., et al. (2017). Foxp3 Reprograms T Cell Metabolism to Function in Low-Glucose, High-Lactate Environments. Cell Metab. *25*, 1282–1293 e1287. https://doi.org/10.1016/j.cmet.2016.12.018.
10. Li, L., Liu, X., Sanders, K.L., Edwards, J.L., Ye, J., Si, F., Gao, A., Huang, L., Hsueh, E.C., Ford, D.A., et al. (2019). TLR8-Mediated Metabolic

Control of Human Treg Function: A Mechanistic Target for Cancer Immunotherapy. Cell Metab. *29*, 103–123.e5. https://doi.org/10.1016/j.cmet.2018.09.020.

11. Procaccini, C., Carbone, F., Di Silvestre, D., Brambilla, F., De Rosa, V., Galgani, M., Faicchia, D., Marone, G., Tramontano, D., Corona, M., et al. (2016). The Proteomic Landscape of Human Ex Vivo Regulatory and Conventional T Cells Reveals Specific Metabolic Requirements. Immunity *44*, 712. https://doi.org/10.1016/j.immuni.2016.02.022.
12. Blagih, J., Coulombe, F., Vincent, E.E., Dupuy, F., Galicia-Vázquez, G., Yurchenko, E., Raissi, T.C., van der Windt, G.J.W., Viollet, B., Pearce, E.L., et al. (2015). The energy sensor AMPK regulates T cell metabolic adaptation and effector responses in vivo. Immunity *42*, 41–54. https://doi.org/10.1016/j.immuni.2014.12.030.
13. Sinclair, L.V., Rolf, J., Emslie, E., Shi, Y.B., Taylor, P.M., and Cantrell, D.A. (2013). Control of amino-acid transport by antigen receptors coordinates the metabolic reprogramming essential for T cell differentiation. Nat. Immunol. *14*, 500–508. https://doi.org/10.1038/ni.2556.
14. Wang, R., Dillon, C.P., Shi, L.Z., Milasta, S., Carter, R., Finkelstein, D., McCormick, L.L., Fitzgerald, P., Chi, H., Munger, J., and Green, D.R. (2011). The transcription factor Myc controls metabolic reprogramming upon T lymphocyte activation. Immunity *35*, 871–882. https://doi.org/10.1016/j.immuni.2011.09.021.
15. O'Sullivan, D. (2019). The metabolic spectrum of memory T cells. Immunol. Cell Biol. *97*, 636–646. https://doi.org/10.1111/imcb.12274.
16. Klein Geltink, R.I., O'Sullivan, D., Corrado, M., Bremser, A., Buck, M.D., Buescher, J.M., Firat, E., Zhu, X., Niedermann, G., Caputa, G., et al. (2017). Mitochondrial Priming by CD28. Cell *171*, 385–397 e311. https://doi.org/10.1016/j.cell.2017.08.018.
17. Almeida, L., Lochner, M., Berod, L., and Sparwasser, T. (2016). Metabolic pathways in T cell activation and lineage differentiation. Semin. Immunol. *28*, 514–524. https://doi.org/10.1016/j.smim.2016.10.009.
18. Shyer, J.A., Flavell, R.A., and Bailis, W. (2020). Metabolic signaling in T cells. Cell Res. *30*, 649–659. https://doi.org/10.1038/s41422-020-0379-5.
19. Kelly, B., and Pearce, E.L. (2020). Amino Assets: How Amino Acids Support Immunity. Cell Metab. *32*, 154–175. https://doi.org/10.1016/j.cmet.2020.06.010.
20. Geiger, R., Rieckmann, J.C., Wolf, T., Basso, C., Feng, Y., Fuhrer, T., Kogadeeva, M., Picotti, P., Meissner, F., Mann, M., et al. (2016). L-Arginine Modulates T Cell Metabolism and Enhances Survival and Anti-tumor Activity. Cell *167*, 829–842.e13. https://doi.org/10.1016/j.cell.2016.09.031.
21. Wang, S., Tsun, Z.Y., Wolfson, R.L., Shen, K., Wyant, G.A., Plovanich, M.E., Yuan, E.D., Jones, T.D., Chantranupong, L., Comb, W., et al. (2015). Metabolism. Lysosomal amino acid transporter SLC38A9 signals arginine sufficiency to mTORC1. Science *347*, 188–194. https://doi.org/10.1126/science.1257132.
22. Marchingo, J.M., Sinclair, L.V., Howden, A.J., and Cantrell, D.A. (2020). Quantitative analysis of how Myc controls T cell proteomes and metabolic pathways during T cell activation. eLife *9*, e53725. https://doi.org/10.7554/eLife.53725.
23. Delgoffe, G.M., Pollizzi, K.N., Waickman, A.T., Heikamp, E., Meyers, D.J., Horton, M.R., Xiao, B., Worley, P.F., and Powell, J.D. (2011). The kinase mTOR regulates the differentiation of helper T cells through the selective activation of signaling by mTORC1 and mTORC2. Nat. Immunol. *12*, 295–303. https://doi.org/10.1038/ni.2005.
24. Marchingo, J.M., Sinclair, L.V., Howden, A.J., and Cantrell, D.A. (2020). Quantitative analysis of how Myc controls T cell proteomes and metabolic pathways during T cell activation. eLife *9*, e53725. https://doi.org/10.7554/eLife.53725.
25. Ikeda, K., Kinoshita, M., Kayama, H., Nagamori, S., Kongpracha, P., Umemoto, E., Okumura, R., Kurakawa, T., Murakami, M., Mikami, N., et al. (2017). Slc3a2 Mediates Branched-Chain Amino-Acid-Dependent Maintenance of Regulatory T Cells. Cell Rep. *21*, 1824–1838. https://doi.org/10.1016/j.celrep.2017.10.082.
26. Aubatin, A., Sako, N., Decrouy, X., Donnadieu, E., Molinier-Frenkel, V., and Castellano, F. (2018). IL4-induced gene 1 is secreted at the immune synapse and modulates TCR activation independently of its enzymatic activity. Eur. J. Immunol. *48*, 106–119. https://doi.org/10.1002/eji.201646769.
27. Santarlasci, V., Maggi, L., Mazzoni, A., Capone, M., Querci, V., Rossi, M.C., Beltrame, L., Cavalieri, D., De Palma, R., Liotta, F., et al. (2014). IL-4-induced gene 1 maintains high Tob1 expression that contributes to TCR unresponsiveness in human T helper 17 cells. Eur. J. Immunol. *44*, 654–661. https://doi.org/10.1002/eji.201344047.
28. Cousin, C., Aubatin, A., Le Gouvello, S., Apetoh, L., Castellano, F., and Molinier-Frenkel, V. (2015). The immunosuppressive enzyme IL4I1 promotes FoxP3(+) regulatory T lymphocyte differentiation. Eur. J. Immunol. *45*, 1772–1782. https://doi.org/10.1002/eji.201445000.
29. Sadik, A., Somarribas Patterson, L.F., Ozturk, S., Mohapatra, S.R., Panitz, V., Secker, P.F., Pfander, P., Loth, S., Salem, H., Prentzell, M.T., et al. (2020). IL4I1 Is a Metabolic Immune Checkpoint that Activates the AHR and Promotes Tumor Progression. Cell *182*, 1252–1270 e1234. https://doi.org/10.1016/j.cell.2020.07.038.
30. Xu, W., Patel, C.H., Zhao, L., Sun, I.H., Oh, M.H., Sun, I.M., Helms, R.S., Wen, J., and Powell, J.D. (2023). GOT1 regulates CD8(+) effector and memory T cell generation. Cell Rep. *42*, 111987. https://doi.org/10.1016/j.celrep.2022.111987.
31. Wambre, E., Bajzik, V., DeLong, J.H., O'Brien, K., Nguyen, Q.A., Speake, C., Gersuk, V.H., DeBerg, H.A., Whalen, E., Ni, C., et al. (2017). A phenotypically and functionally distinct human T. Sci. Transl. Med. *9*, eaam9171. https://doi.org/10.1126/scitranslmed.aam9171.
32. Mitson-Salazar, A., Yin, Y., Wansley, D.L., Young, M., Bolan, H., Arceo, S., Ho, N., Koh, C., Milner, J.D., Stone, K.D., et al. (2016). Hematopoietic prostaglandin D synthase defines a proeosinophilic pathogenic effector human T(H)2 cell subpopulation with enhanced function. J. Allergy Clin. Immunol. *137*, 907–918.e9. https://doi.org/10.1016/j.jaci.2015.08.007.
33. Vandamme, C., Rytkonen-Nissinen, M., Lonnberg, T., Randell, J., Harvima, R.J., Kinnunen, T., and Virtanen, T. (2022). Single-cell characterization of dog allergen-specific T cells reveals T(H)2 heterogeneity in allergic individuals. J. Allergy Clin. Immunol. *149*, 1732–1743 e1715. https://doi.org/10.1016/j.jaci.2021.11.018.
34. Bangert, C., Rindler, K., Krausgruber, T., Alkon, N., Thaler, F.M., Kurz, H., Ayub, T., Demirtas, D., Fortelny, N., Vorstandlechner, V., et al. (2021). Persistence of mature dendritic cells, T(H)2A, and Tc2 cells characterize clinically resolved atopic dermatitis under IL-4Ralpha blockade. Sci. Immunol. *6*, eabe2749. https://doi.org/10.1126/sciimmunol.abe2749.
35. Henriksson, J., Chen, X., Gomes, T., Ullah, U., Meyer, K.B., Miragaia, R., Duddy, G., Pramanik, J., Yusa, K., Lahesmaa, R., and Teichmann, S.A. (2019). Genome-wide CRISPR Screens in T Helper Cells Reveal Pervasive Crosstalk between Activation and Differentiation. Cell *176*, 882–896.e18. https://doi.org/10.1016/j.cell.2018.11.044.
36. Yang, K., Shrestha, S., Zeng, H., Karmaus, P.W.F., Neale, G., Vogel, P., Guertin, D.A., Lamb, R.F., and Chi, H. (2013). T cell exit from quiescence and differentiation into Th2 cells depend on Raptor-mTORC1-mediated metabolic reprogramming. Immunity *39*, 1043–1056. https://doi.org/10.1016/j.immuni.2013.09.015.
37. Lee, K., Gudapati, P., Dragovic, S., Spencer, C., Joyce, S., Killeen, N., Magnuson, M.A., and Boothby, M. (2010). Mammalian target of rapamycin protein complex 2 regulates differentiation of Th1 and Th2 cell subsets via distinct signaling pathways. Immunity *32*, 743–753. https://doi.org/10.1016/j.immuni.2010.06.002.
38. Berod, L., Friedrich, C., Nandan, A., Freitag, J., Hagemann, S., Harmrolfs, K., Sandouk, A., Hesse, C., Castro, C.N., Bähre, H., et al. (2014). De novo fatty acid synthesis controls the fate between regulatory T and T helper 17 cells. Nat. Med. *20*, 1327–1333. https://doi.org/10.1038/nm.3704.

39. Obeso, D., Mera-Berriatua, L., Rodríguez-Coira, J., Rosace, D., Fernández, P., Martín-Antoniano, I.A., Santaolalla, M., Marco Martín, G., Chivato, T., Fernández-Rivas, M., et al. (2018). Multi-omics analysis points to altered platelet functions in severe food-associated respiratory allergy. Allergy *73*, 2137–2149. https://doi.org/10.1111/all.13563.

40. Crestani, E., Harb, H., Charbonnier, L.M., Leirer, J., Motsinger-Reif, A., Rachid, R., Phipatanakul, W., Kaddurah-Daouk, R., and Chatila, T.A. (2020). Untargeted metabolomic profiling identifies disease-specific signatures in food allergy and asthma. J. Allergy Clin. Immunol. *145*, 897–906. https://doi.org/10.1016/j.jaci.2019.10.014.

41. Perales-Chorda, C., Obeso, D., Twomey, L., Rojas-Benedicto, A., Puchades-Carrasco, L., Roca, M., Pineda-Lucena, A., Laguna, J.J., Barbas, C., Esteban, V., et al. (2021). Characterization of anaphylaxis reveals different metabolic changes depending on severity and triggers. Clin. Exp. Allergy *51*, 1295–1309. https://doi.org/10.1111/cea.13991.

42. Sokolowska, M., Chen, L.Y., Liu, Y., Martinez-Anton, A., Logun, C., Alsaaty, S., Cuento, R.A., Cai, R., Sun, J., Quehenberger, O., et al. (2017). Dysregulation of lipidomic profile and antiviral immunity in response to hyaluronan in patients with severe asthma. J. Allergy Clin. Immunol. *139*, 1379–1383. https://doi.org/10.1016/j.jaci.2016.09.031.

43. Reinke, S.N., Gallart-Ayala, H., Gomez, C., Checa, A., Fauland, A., Naz, S., Kamleh, M.A., Djukanovic, R., Hinks, T.S., and Wheelock, C.E. (2017). Metabolomics analysis identifies different metabotypes of asthma severity. Eur. Respir. J. *49*, 1601740. https://doi.org/10.1183/13993003.01740-2016.

44. Kamburov, A., Cavill, R., Ebbels, T.M.D., Herwig, R., and Keun, H.C. (2011). Integrated pathway-level analysis of transcriptomics and metabolomics data with IMPaLA. Bioinformatics *27*, 2917–2918. https://doi.org/10.1093/bioinformatics/btr499.

45. Wishart, D.S., Guo, A., Oler, E., Wang, F., Anjum, A., Peters, H., Dizon, R., Sayeeda, Z., Tian, S., Lee, B.L., et al. (2022). HMDB 5.0: the Human Metabolome Database for 2022. Nucleic Acids Res. *50*, D622–D631. https://doi.org/10.1093/nar/gkab1062.

46. Boulland, M.L., Marquet, J., Molinier-Frenkel, V., Möller, P., Guiter, C., Lasoudris, F., Copie-Bergman, C., Baia, M., Gaulard, P., Leroy, K., and Castellano, F. (2007). Human IL4I1 is a secreted L-phenylalanine oxidase expressed by mature dendritic cells that inhibits T-lymphocyte proliferation. Blood *110*, 220–227. https://doi.org/10.1182/blood-2006-07-036210.

47. Santarlasci, V., Maggi, L., Capone, M., Querci, V., Beltrame, L., Cavalieri, D., D'Aiuto, E., Cimaz, R., Nebbioso, A., Liotta, F., et al. (2012). Rarity of human T helper 17 cells is due to retinoic acid orphan receptor-dependent mechanisms that limit their expansion. Immunity *36*, 201–214. https://doi.org/10.1016/j.immuni.2011.12.013.

48. Arguello, R.J., Combes, A.J., Char, R., Gigan, J.P., Baaziz, A.I., Bousiquot, E., Camosseto, V., Samad, B., Tsui, J., Yan, P., et al. (2020). SCENITH: A Flow Cytometry-Based Method to Functionally Profile Energy Metabolism with Single-Cell Resolution. Cell Metab. *32*, 1063–1075 e1067. https://doi.org/10.1016/j.cmet.2020.11.007.

49. Cousins, D.J., Lee, T.H., and Staynov, D.Z. (2002). Cytokine coexpression during human Th1/Th2 cell differentiation: direct evidence for coordinated expression of Th2 cytokines. J. Immunol. *169*, 2498–2506. https://doi.org/10.4049/jimmunol.169.5.2498.

50. Wambre, E., Bajzik, V., DeLong, J.H., O'Brien, K., Nguyen, Q.A., Speake, C., Gersuk, V.H., DeBerg, H.A., Whalen, E., Ni, C., et al. (2017). A phenotypically and functionally distinct human T(H)2 cell subpopulation is associated with allergic disorders. Sci. Transl. Med. *9*, eaam9171. https://doi.org/10.1126/scitranslmed.aam9171.

51. Yuan, Y., Wang, C., Wang, G., Guo, X., Jiang, S., Zuo, X., Wang, X., Hsu, A.C.Y., Qi, M., and Wang, F. (2021). Airway Microbiome and Serum Metabolomics Analysis Identify Differential Candidate Biomarkers in Allergic Rhinitis. Front. Immunol. *12*, 771136. https://doi.org/10.3389/fimmu.2021.771136.

52. Delgado-Dolset, M.I., Obeso, D., Rodríguez-Coira, J., Tarin, C., Tan, G., Cumplido, J.A., Cabrera, A., Angulo, S., Barbas, C., Sokolowska, M., et al. (2022). Understanding uncontrolled severe allergic asthma by integration of omic and clinical data. Allergy *77*, 1772–1785. https://doi.org/10.1111/all.15192.

53. Rahimi, R.A., Nepal, K., Cetinbas, M., Sadreyev, R.I., and Luster, A.D. (2020). Distinct functions of tissue-resident and circulating memory Th2 cells in allergic airway disease. J. Exp. Med. *217*, e20190865. https://doi.org/10.1084/jem.20190865.

54. Barber, D., Moreno, C., Ledesma, A., Serrano, P., Galán, A., Villalba, M., Guerra, F., Lombardero, M., and Rodríguez, R. (2007). Degree of olive pollen exposure and sensitization patterns. Clinical implications. J Investig. Allergol. Clin. Immunol. *17*, 11–16.

55. Barber, D., de la Torre, F., Feo, F., Florido, F., Guardia, P., Moreno, C., Quiralte, J., Lombardero, M., Villalba, M., Salcedo, G., and Rodríguez, R. (2008). Understanding patient sensitization profiles in complex pollen areas: a molecular epidemiological study. Allergy *63*, 1550–1558. https://doi.org/10.1111/j.1398-9995.2008.01807.x.

56. Boonpiyathad, T., Capova, G., Duchna, H.W., Croxford, A.L., Farine, H., Dreher, A., Clozel, M., Schreiber, J., Kubena, P., Lunjani, N., et al. (2020). Impact of high-altitude therapy on type-2 immune responses in asthma patients. Allergy *75*, 84–94. https://doi.org/10.1111/all.13967.

57. Chantveerawong, T., Sangkangjanavanich, S., Chiewchalermsri, C., Pradubpongsa, P., Mitthamsiri, W., Jindarat, S., Wang, M., Akdis, M., Sokolowska, M., Akdis, C.A., et al. (2022). Increased circulating CRTH2(+) Tregs are associated with asthma control and exacerbation. Allergy *77*, 681–685. https://doi.org/10.1111/all.15145.

58. Tan, C.L., Kuchroo, J.R., Sage, P.T., Liang, D., Francisco, L.M., Buck, J., Thaker, Y.R., Zhang, Q., McArdel, S.L., Juneja, V.R., et al. (2021). PD-1 restraint of regulatory T cell suppressive activity is critical for immune tolerance. J. Exp. Med. *218*, e20182232. https://doi.org/10.1084/jem.20182232.

59. Lao-Araya, M., Steveling, E., Scadding, G.W., Durham, S.R., and Shamji, M.H. (2014). Seasonal increases in peripheral innate lymphoid type 2 cells are inhibited by subcutaneous grass pollen immunotherapy. J. Allergy Clin. Immunol. *134*, 1193–1195.e4. https://doi.org/10.1016/j.jaci.2014.07.029.

60. Zhong, H., Fan, X.L., Yu, Q.N., Qin, Z.L., Chen, D., Xu, R., Chen, D.H., Lin, Z.B., Wen, W., and Fu, Q.L. (2017). Increased innate type 2 immune response in house dust mite-allergic patients with allergic rhinitis. Clin. Immunol. *183*, 293–299. https://doi.org/10.1016/j.clim.2017.09.008.

61. Fan, D., Wang, X., Wang, M., Wang, Y., Zhang, L., Li, Y., Fan, E., Cao, F., Van Crombruggen, K., and Zhang, L. (2016). Allergen-Dependent Differences in ILC2s Frequencies in Patients With Allergic Rhinitis. Allergy Asthma Immunol. Res. *8*, 216–222. https://doi.org/10.4168/aair.2016.8.3.216.

62. MacLean Scott, E., Solomon, L.A., Davidson, C., Storie, J., Palikhe, N.S., and Cameron, L. (2018). Activation of Th2 cells downregulates CRTh2 through an NFAT1 mediated mechanism. PLoS One *13*, e0199156. https://doi.org/10.1371/journal.pone.0199156.

63. Seumois, G., Zapardiel-Gonzalo, J., White, B., Singh, D., Schulten, V., Dillon, M., Hinz, D., Broide, D.H., Sette, A., Peters, B., and Vijayanand, P. (2016). Transcriptional Profiling of Th2 Cells Identifies Pathogenic Features Associated with Asthma. J. Immunol. *197*, 655–664. https://doi.org/10.4049/jimmunol.1600397.

64. Pablo-Torres, C., Garcia-Escribano, C., Romeo, M., Gomez-Casado, C., Arroyo Solera, R., Bueno-Cabrera, J.L., Del Mar Reaño Martos, M., Iglesias-Cadarso, A., Tarín, C., Agache, I., et al. (2023). Transcriptomics reveals a distinct metabolic profile in T cells from severe allergic asthmatic patients. Front. Allergy *4*, 1129248. https://doi.org/10.3389/falgy.2023.1129248.

65. Obeso, D., Contreras, N., Dolores-Hernandez, M., Carrillo, T., Barbas, C., Escribese, M.M., Villasenor, A., and Barber, D. (2022). Development of a Novel Targeted Metabolomic LC-QqQ-MS Method in Allergic Inflammation. Metabolites *12*, 592. https://doi.org/10.3390/metabo12070592.

66. Eljaszewicz, A., Ruchti, F., Radzikowska, U., Globinska, A., Boonpiyathad, T., Gschwend, A., Morita, H., Helbling, A., Arasi, S., Kahlert, H., et al. (2021). Trained immunity and tolerance in innate lymphoid cells, monocytes, and dendritic cells during allergen-specific immunotherapy. J. Allergy Clin. Immunol. *147*, 1865–1877. https://doi.org/10.1016/j.jaci.2020.08.042.

67. Gerriets, V.A., Kishton, R.J., Nichols, A.G., Macintyre, A.N., Inoue, M., Ilkayeva, O., Winter, P.S., Liu, X., Priyadharshini, B., Slawinska, M.E., et al. (2015). Metabolic programming and PDHK1 control CD4+ T cell subsets and inflammation. J. Clin. Investig. *125*, 194–207. https://doi.org/10.1172/JCI76012.

68. Qiu, J., Villa, M., Sanin, D.E., Buck, M.D., O'Sullivan, D., Ching, R., Matsushita, M., Grzes, K.M., Winkler, F., Chang, C.H., et al. (2019). Acetate Promotes T Cell Effector Function during Glucose Restriction. Cell Rep. *27*, 2063–2074.e5. https://doi.org/10.1016/j.celrep.2019.04.022.

69. Ma, E.H., Verway, M.J., Johnson, R.M., Roy, D.G., Steadman, M., Hayes, S., Williams, K.S., Sheldon, R.D., Samborska, B., Kosinski, P.A., et al. (2019). Metabolic Profiling Using Stable Isotope Tracing Reveals Distinct Patterns of Glucose Utilization by Physiologically Activated CD8(+) T Cells. Immunity *51*, 856–870.e5. https://doi.org/10.1016/j.immuni.2019.09.003.

70. de Kivit, S., Mensink, M., Hoekstra, A.T., Berlin, I., Derks, R.J.E., Both, D., Aslam, M.A., Amsen, D., Berkers, C.R., and Borst, J. (2020). Stable human regulatory T cells switch to glycolysis following TNF receptor 2 costimulation. Nat. Metab. *2*, 1046–1061. https://doi.org/10.1038/s42255-020-00271-w.

71. Zeng, H., Cohen, S., Guy, C., Shrestha, S., Neale, G., Brown, S.A., Cloer, C., Kishton, R.J., Gao, X., Youngblood, B., et al. (2016). mTORC1 and mTORC2 Kinase Signaling and Glucose Metabolism Drive Follicular Helper T Cell Differentiation. Immunity *45*, 540–554. https://doi.org/10.1016/j.immuni.2016.08.017.

72. Jungnickel, K.E.J., Parker, J.L., and Newstead, S. (2018). Structural basis for amino acid transport by the CAT family of SLC7 transporters. Nat. Commun. *9*, 550. https://doi.org/10.1038/s41467-018-03066-6.

73. Kanai, Y., Segawa, H., Miyamoto, K.i., Uchino, H., Takeda, E., and Endou, H. (1998). Expression cloning and characterization of a transporter for large neutral amino acids activated by the heavy chain of 4F2 antigen (CD98). J. Biol. Chem. *273*, 23629–23632. https://doi.org/10.1074/jbc.273.37.23629.

74. Segawa, H., Fukasawa, Y., Miyamoto, K., Takeda, E., Endou, H., and Kanai, Y. (1999). Identification and functional characterization of a Na+-independent neutral amino acid transporter with broad substrate selectivity. J. Biol. Chem. *274*, 19745–19751. https://doi.org/10.1074/jbc.274.28.19745.

75. Karlsson, M., Zhang, C., Mear, L., Zhong, W., Digre, A., Katona, B., Sjostedt, E., Butler, L., Odeberg, J., Dusart, P., et al. (2021). A single-cell type transcriptomics map of human tissues. Sci. Adv. *7*, abh2169. https://doi.org/10.1126/sciadv.abh2169.

76. Panteghini, M. (1990). Aspartate aminotransferase isoenzymes. Clin. Biochem. *23*, 311–319. https://doi.org/10.1016/0009-9120(90)80062-n.

77. Mason, J.M., Naidu, M.D., Barcia, M., Porti, D., Chavan, S.S., and Chu, C.C. (2004). IL-4-induced gene-1 is a leukocyte L-amino acid oxidase with an unusual acidic pH preference and lysosomal localization. J. Immunol. *173*, 4561–4567. https://doi.org/10.4049/jimmunol.173.7.4561.

78. Shi, H., Chapman, N.M., Wen, J., Guy, C., Long, L., Dhungana, Y., Rankin, S., Pelletier, S., Vogel, P., Wang, H., et al. (2019). Amino Acids License Kinase mTORC1 Activity and Treg Cell Function via Small G Proteins Rag and Rheb. Immunity *51*, 1012–1027.e7. https://doi.org/10.1016/j.immuni.2019.10.001.

79. Chi, H. (2012). Regulation and function of mTOR signalling in T cell fate decisions. Nat. Rev. Immunol. *12*, 325–338. https://doi.org/10.1038/nri3198.

80. Zhu, J., Guo, L., Watson, C.J., Hu-Li, J., and Paul, W.E. (2001). Stat6 is necessary and sufficient for IL-4's role in Th2 differentiation and cell expansion. J. Immunol. *166*, 7276–7281. https://doi.org/10.4049/jimmunol.166.12.7276.

81. Ma, S., Ming, Y., Wu, J., and Cui, G. (2024). Cellular metabolism regulates the differentiation and function of T-cell subsets. Cell. Mol. Immunol. *21*, 419–435. https://doi.org/10.1038/s41423-024-01148-8.

82. Pandit, M., Timilshina, M., Gu, Y., Acharya, S., Chung, Y., Seo, S.U., and Chang, J.H. (2022). AMPK suppresses Th2 cell responses by repressing mTORC2. Exp. Mol. Med. *54*, 1214–1224. https://doi.org/10.1038/s12276-022-00832-x.

83. Wishart, D.S., Tzur, D., Knox, C., Eisner, R., Guo, A.C., Young, N., Cheng, D., Jewell, K., Arndt, D., Sawhney, S., et al. (2007). HMDB: the human metabolome database. Nucleic acids research *35*, D521–D526.

84. Cleary, M., Trefz, F., Muntau, A.C., Feillet, F., van Spronsen, F.J., Burlina, A., Bélanger-Quintana, A., Giżewska, M., Gasteyger, C., Bettiol, E., et al. (2013). Fluctuations in phenylalanine concentrations in phenylketonuria: a review of possible relationships with outcomes. Mol. Genet. Metab. *110*, 418–423. https://doi.org/10.1016/j.ymgme.2013.09.001.

85. Williams, R.A., Mamotte, C.D.S., and Burnett, J.R. (2008). Phenylketonuria: an inborn error of phenylalanine metabolism. Clin. Biochem. Rev. *29*, 31–41.

86. Cottrill, K.A., Chandler, J.D., Kobara, S., Stephenson, S.T., Mohammad, A.F., Tidwell, M., Mason, C., Van Dresser, M., Patrignani, J., Kamaleswaran, R., et al. (2023). Metabolomics identifies disturbances in arginine, phenylalanine, and glycine metabolism as differentiating features of exacerbating atopic asthma in children. J. Allergy Clin. Immunol. Glob. *2*, 100115. https://doi.org/10.1016/j.jacig.2023.100115.

87. Hodge, S.H., Krauss, M.Z., Kaymak, I., King, J.I., Howden, A.J.M., Panic, G., Grencis, R.K., Swann, J.R., Sinclair, L.V., and Hepworth, M.R. (2023). Amino acid availability acts as a metabolic rheostat to determine the magnitude of ILC2 responses. J. Exp. Med. *220*, e20221073. https://doi.org/10.1084/jem.20221073.

88. Healey, D.C.C., Cephus, J.Y., Barone, S.M., Chowdhury, N.U., Dahunsi, D.O., Madden, M.Z., Ye, X., Yu, X., Olszewski, K., Young, K., et al. (2021). Targeting In Vivo Metabolic Vulnerabilities of Th2 and Th17 Cells Reduces Airway Inflammation. J. Immunol. *206*, 1127–1139. https://doi.org/10.4049/jimmunol.2001029.

89. Trefz, K.F., Muntau, A.C., Kohlscheen, K.M., Altevers, J., Jacob, C., Braun, S., Greiner, W., Jha, A., Jain, M., Alvarez, I., et al. (2019). Clinical burden of illness in patients with phenylketonuria (PKU) and associated comorbidities - a retrospective study of German health insurance claims data. Orphanet J. Rare Dis. *14*, 181. https://doi.org/10.1186/s13023-019-1153-y.

90. Varona, R., Ramos, T., Escribese, M.M., Jimeno, L., Galán, A., Würtzen, P.A., Vega, F., Marín, A., Martín, S., Carrera, A.C., et al. (2019). Persistent regulatory T-cell response 2 years after 3 years of grass tablet SLIT: Links to reduced eosinophil counts, sIgE levels, and clinical benefit. Allergy *74*, 349–360. https://doi.org/10.1111/all.13553.

91. Boonpiyathad, T., Sokolowska, M., Morita, H., Rückert, B., Kast, J.I., Wawrzyniak, M., Sangasapaviliya, A., Pradubpongsa, P., Fuengthong, R., Thantiworasit, P., et al. (2019). Der p 1-specific regulatory T-cell response during house dust mite allergen immunotherapy. Allergy *74*, 976–985. https://doi.org/10.1111/all.13684.

92. Cardoso-Vigueros, C., von Blumenthal, T., Rückert, B., Rinaldi, A.O., Tan, G., Dreher, A., Radzikowska, U., Menz, G., Schmid-Grendelmeier, P., Akdis, C.A., and Sokolowska, M. (2022). Leukocyte redistribution as immunological biomarker of corticosteroid resistance in severe asthma. Clin. Exp. Allergy *52*, 1183–1194. https://doi.org/10.1111/cea.14128.

93. Kabat, A.M., Hackl, A., Sanin, D.E., Zeis, P., Grzes, K.M., Baixauli, F., Kyle, R., Caputa, G., Edwards-Hicks, J., Villa, M., et al. (2022). Resident T(H)2 cells orchestrate adipose tissue remodeling at a site adjacent to infection. Sci. Immunol. *7*, eadd3263. https://doi.org/10.1126/sciimmunol.add3263.

CellPress
OPEN ACCESS

94. Sud, M., Fahy, E., Cotter, D., Azam, K., Vadivelu, I., Burant, C., Edison, A., Fiehn, O., Higashi, R., Nair, K.S., et al. (2016). Metabolomics Workbench: An international repository for metabolomics data and metadata, metabolite standards, protocols, tutorials and training, and analysis tools. Nucleic Acids Res. *44*, D463–D470. https://doi.org/10.1093/nar/gkv1042.

95. Gil-de-la-Fuente, A., Godzien, J., Saugar, S., Garcia-Carmona, R., Badran, H., Wishart, D.S., Barbas, C., and Otero, A. (2019). CEU Mass Mediator 3.0: A Metabolite Annotation Tool. J. Proteome Res. *18*, 797–802. https://doi.org/10.1021/acs.jproteome.8b00720.

96. Fletez-Brant, K., Špidlen, J., Brinkman, R.R., Roederer, M., and Chattopadhyay, P.K. (2016). flowClean: Automated identification and removal of fluorescence anomalies in flow cytometry data. Cytometry. A. *89*, 461–471. https://doi.org/10.1002/cyto.a.22837.

97. Yu, G., Wang, L.G., Han, Y., and He, Q.Y. (2012). clusterProfiler: an R package for comparing biological themes among gene clusters. OMICS *16*, 284–287. https://doi.org/10.1089/omi.2011.0118.

98. Schindelin, J., Arganda-Carreras, I., Frise, E., Kaynig, V., Longair, M., Pietzsch, T., Preibisch, S., Rueden, C., Saalfeld, S., Schmid, B., et al. (2012). Fiji: an open-source platform for biological-image analysis. Nat. Methods *9*, 676–682. https://doi.org/10.1038/nmeth.2019.

99. Yang, X., Zhu, X., Sheng, J., Fu, Y., Nie, D., You, X., Chen, Y., Yang, X., Ling, Q., Zhang, H., et al. (2024). RNF213 promotes Treg cell differentiation by facilitating K63-linked ubiquitination and nuclear translocation of FOXO1. Nat. Commun. *15*, 5961. https://doi.org/10.1038/s41467-024-50392-z.

100. Belkina, A.C., Ciccolella, C.O., Anno, R., Halpert, R., Spidlen, J., and Snyder-Cappione, J.E. (2019). Automated optimized parameters for T-distributed stochastic neighbor embedding improve visualization and analysis of large datasets. Nat. Commun. *10*, 5415. https://doi.org/10.1038/s41467-019-13055-y.

101. Van Gassen, S., Callebaut, B., Van Helden, M.J., Lambrecht, B.N., Demeester, P., Dhaene, T., and Saeys, Y. (2015). FlowSOM: Using self-organizing maps for visualization and interpretation of cytometry data. Cytometry. A. *87*, 636–645. https://doi.org/10.1002/cyto.a.22625.

102. Melero-Fernandez de Mera, R.M., Villaseñor, A., Rojo, D., Carrión-Navarro, J., Gradillas, A., Ayuso-Sacido, A., and Barbas, C. (2021). Ceramide Composition in Exosomes for Characterization of Glioblastoma Stem-Like Cell Phenotypes. Front. Oncol. *11*, 788100. https://doi.org/10.3389/fonc.2021.788100.

103. Binek, A., Rojo, D., Godzien, J., Rupérez, F.J., Nuñez, V., Jorge, I., Ricote, M., Vázquez, J., and Barbas, C. (2019). Flow Cytometry Has a Significant Impact on the Cellular Metabolome. J. Proteome Res. *18*, 169–181. https://doi.org/10.1021/acs.jproteome.8b00472.

104. Armitage, E.G., Godzien, J., Alonso-Herranz, V., López-Gonzálvez, Á., and Barbas, C. (2015). Missing value imputation strategies for metabolomics data. Electrophoresis *36*, 3050–3060. https://doi.org/10.1002/elps.201500352.

105. Kellogg, J.J., Kvalheim, O.M., and Cech, N.B. (2020). Composite score analysis for unsupervised comparison and network visualization of metabolomics data. Anal. Chim. Acta *1095*, 38–47. https://doi.org/10.1016/j.aca.2019.10.029.

106. Gil de la Fuente, A., Godzien, J., Fernández López, M., Rupérez, F.J., Barbas, C., and Otero, A. (2018). Knowledge-based metabolite annotation tool: CEU Mass Mediator. J. Pharm. Biomed. Anal. *154*, 138–149. https://doi.org/10.1016/j.jpba.2018.02.046.

107. Koelmel, J.P., Li, X., Stow, S.M., Sartain, M.J., Murali, A., Kemperman, R., Tsugawa, H., Takahashi, M., Vasiliou, V., Bowden, J.A., et al. (2020). Lipid Annotator: Towards Accurate Annotation in Non-Targeted Liquid Chromatography High-Resolution Tandem Mass Spectrometry (LC-HRMS/MS) Lipidomics Using A Rapid and User-Friendly Software. Metabolites *10*, 101. https://doi.org/10.3390/metabo10030101.

108. Tsugawa, H., Ikeda, K., Takahashi, M., Satoh, A., Mori, Y., Uchino, H., Okahashi, N., Yamada, Y., Tada, I., Bonini, P., et al. (2020). A lipidome atlas in MS-DIAL 4. Nat. Biotechnol. *38*, 1159–1163. https://doi.org/10.1038/s41587-020-0531-2.

109. Han, X. (2016). Lipidomics: Comprehensive Mass Spectrometry of Lipids (John Wiley & Sons). https://doi.org/10.1002/9781119085263.

110. Hatakeyama, M., Opitz, L., Russo, G., Qi, W., Schlapbach, R., and Rehrauer, H. (2016). SUSHI: an exquisite recipe for fully documented, reproducible and reusable NGS data analysis. BMC Bioinf. *17*, 228. https://doi.org/10.1186/s12859-016-1104-8.

111. S, A. (2010). FastQC: A Quality Control Tool for High Throughput Sequence Data. http://www.bioinformatics.babraham.ac.uk/projects/fastqc/.

112. Chen, S., Zhou, Y., Chen, Y., and Gu, J. (2018). fastp: an ultra-fast all-in-one FASTQ preprocessor. Bioinformatics *34*, i884–i890. https://doi.org/10.1093/bioinformatics/bty560.

113. Bray, N.L., Pimentel, H., Melsted, P., and Pachter, L. (2016). Near-optimal probabilistic RNA-seq quantification. Nat. Biotechnol. *34*, 525–527. https://doi.org/10.1038/nbt.3519.

114. Frankish, A., Diekhans, M., Jungreis, I., Lagarde, J., Loveland, J.E., Mudge, J.M., Sisu, C., Wright, J.C., Armstrong, J., Barnes, I., et al. (2021). Gencode 2021. Nucleic Acids Res. *49*, D916–D923. https://doi.org/10.1093/nar/gkaa1087.

115. Love, M.I., Huber, W., and Anders, S. (2014). Moderated estimation of fold change and dispersion for RNA-seq data with DESeq2. Genome Biol. *15*, 550. https://doi.org/10.1186/s13059-014-0550-8.

116. Benjamini, Y.,K.A.M., and Yekutieli, D. (2006). Adaptive linear step-up procedures that control the false discovery rate. Biometrika *93*, 491–507. https://doi.org/10.1093/biomet/93.3.491.

STAR★METHODS

KEY RESOURCES TABLE

REAGENT or RESOURCE	SOURCE	IDENTIFIER
Antibodies		
Mouse anti-GATA3-BUV395	BD Biosciences	Cat#565448; RRID: AB_2739241
Mouse anti-human CD4-BUV737	BD Biosciences	Cat#612748; RRID: AB_2870079
Rat anti-human IL4-BV421	BD Biosciences	Cat#564110; RRID: AB_2738599
Mouse anti-human CD3-BV510	BD Biosciences	Cat#563109; RRID: AB_2732053
Mouse anti-human CD161-BV605	BD Biosciences	Cat#747762; RRID: AB_2872229
Rat anti-human and viral IL10-BV711	BD Biosciences	Cat#564050; RRID: AB_2738564
Mouse anti-human CD127-BV786	BD Biosciences	Cat#563324; RRID: AB_2738138
Mouse anti-human CD25-BB515	BD Biosciences	Cat#565096; RRID: AB_2739065
Mouse anti-human FoxP3-RB705	BD Biosciences	Cat#570239; RRID: AB_3685601
Mouse anti-human IL17A-PE	BD Biosciences	Cat#560436; RRID: AB_1645514
Mouse anti-human RORγt-PE-CF594	BD Biosciences	Cat#567532; RRID: AB_2916639
Mouse anti-human T-bet-RY703	BD Biosciences	Cat#571477; RRID: AB_3686550
Mouse anti-human IFN-γ-RY775	BD Biosciences	Cat#571386; RRID: AB_3686466
Mouse anti-human CD45RA-R718	BD Biosciences	Cat#567073; RRID: AB_2916420
Mouse anti-human CD4-BV786	BD Biosciences	Cat#563877; RRID: AB_2738462
Mouse anti-human GATA3-BV711	BD Biosciences	Cat#565449; RRID: AB_2739242
Mouse anti-human CD161-BV650	BD Biosciences	Cat#748283; RRID: AB_2872711
Mouse anti-human CD45RA-BV510	BD Biosciences	Cat#563031; RRID: AB_2722499
Mouse anti-human CD25-BV421	BD Biosciences	Cat#564033; RRID: AB_2738555
Mouse anti-human FOXP3-BB700	BD Biosciences	Cat#566526; RRID: AB_2744476
Mouse anti-human CD3-FITC	BD Biosciences	Cat#555332; RRID: AB_395739
Mouse anti-human CCR4-PE-Cy7	BD Biosciences	Cat#557864; RRID: AB_396907
Rat anti-human CRTH2-PE-CF594	BD Biosciences	Cat#563501; RRID: AB_2738244
Mouse anti-human CD127-R718	BD Biosciences	Cat#566967; RRID: AB_2869977
Mouse anti-human CD8-APC-Cy7	BioLegend	Cat#344714; RRID: AB_2044006
Mouse anti-human CD14-APC-Cy7	BioLegend	Cat#325620; RRID: AB_830693
Mouse anti-human CD16-APC-Cy7	BioLegend	Cat#302018; RRID: AB_314218
Mouse anti-human CD19-APC-Cy7	BioLegend	Cat#302218; RRID: AB_314248
Mouse anti-human CD1a-FITC	BioLegend	Cat#300104; RRID: AB_314018
Mouse anti-human CD11c-FITC	BioLegend	Cat#301604; RRID: AB_314174
Mouse anti-human CD34-FITC	BioLegend	Cat#343504; RRID: AB_1731852
Mouse anti-human CD94-FITC	BioLegend	Cat#305504; RRID: AB_314534
Mouse anti-human CD123-FITC	BioLegend	Cat#306014; RRID: AB_2124259
Mouse anti-human CD303-FITC	BioLegend	Cat#354208; RRID: AB_2561364
Mouse anti-human FceR1a-FITC	BioLegend	Cat#334608; RRID: AB_1227653
Rat anti-human CRTH2-PE	BD Biosciences	Cat#563665; RRID: AB_2738360
Mouse anti-human CD117-PE-CF594	BD Biosciences	Cat#562407; RRID: AB_11154595
Mouse anti-human CD8-PerCP-Cy5.5	BioLegend	Cat#344710; RRID: AB_2044010
Mouse anti-human CD45RA-PE-Cy7	BD Biosciences	Cat#568557; RRID: AB_3166166
Mouse anti-human CD161-AF647	BioLegend	Cat#339910; RRID: AB_1574977
Mouse anti-human CD4-AF700	BioLegend	Cat#344622; RRID: AB_2563150
Mouse anti-human CD25-BV421	BioLegend	Cat#302630; RRID: AB_11126749
Mouse anti-human CD3-V500	BD Biosciences	Cat#561416; RRID: AB_10611584

(*Continued on next page*)

Continued

REAGENT or RESOURCE	SOURCE	IDENTIFIER
Mouse anti-human CD127-BV605	BioLegend	Cat#351334; RRID: AB_2562022
Mouse anti-human PD1-BV785	BioLegend	Cat#329930; RRID: AB_2563443
Mouse anti-human CD45-BV510	BD Biosciences	Cat#563204; RRID: AB_2738067
Mouse anti-human CD45-FITC	BioLegend	Cat#304038; RRID: AB_2562050
Rat anti-human IL10 (JES3-9D7)	Thermo Fisher Scientific	Cat#16-7108-85; RRID: AB_469228
Mouse anti-human IFNγ (NIB42)	Thermo Fisher Scientific	Cat#16-7318-85; RRID: AB_469251
Rat anti-human IL4 (MP4-25D2)	Thermo Fisher Scientific	Cat#16-7048-85; RRID: AB_469210
Rabbit anti-human LAT1	Cell Signaling Technology	Cat#5347; RRID: AB_10695104
Rabbit anti-human IL4I1	Thermo Fisher Scientific	Cat#PA5-113266; RRID: AB_2868000
mTOR (7C10) Rabbit mAb	Cell Signaling Technology	Cat#2983; RRID: AB_2105622
Stat6 (D3H4) Rabbit mAb	Cell Signaling Technology	Cat#5397; RRID: AB_11220421
Phospho-mTOR (Ser2448) (D9C2) XP® Rabbit mAb	Cell Signaling Technology	Cat#5536; RRID: AB_10691552
Phospho-Stat6 (Tyr641) Antibody	Cell Signaling Technology	Cat#9361; RRID: AB_331595
HRP conjugated goat anti-rabbit IgG (H + L)	Jackson Laboratories	Cat#111-035-144; RRID: AB_2307391
HRP conjugated anti-beta actin	Thermo Fisher Scientific	Cat#MA5-15739-HRP; RRID: AB_2537667
Chemicals, peptides, and recombinant proteins		
Heat inactivated Fetal Calf Serum	Sigma-Aldrich	Cat#F7524
DMSO	Sigma-Aldrich	Cat#D2650-100ML
Bovine Serum Albumin	Sigma-Aldrich	Cat#A3294-500G
EDTA	Sigma-Aldrich	Cat# 60-00-4
RPMI1640	Lonza	Cat#12-115Q/12
100X Penicillin/Streptomycin	Sigma-Aldrich	Cat#P4333-100ML
100X MEM Vitamins	Sigma-Aldrich	Cat#M6895-100ML
MEM non-essential amino acids	Sigma-Aldrich	Cat#M7145-100ML
Sodium Pyruvate	Sigma-Aldrich	Cat#S8636-100ML
Recombinant human IL2	PeproTech	Cat#200-02
Recombinant human IL4	PeproTech	Cat#200-04
Recombinant human IL6	PeproTech	Cat#200-06
Recombinant human IL12	STEMCELL Technologies	Cat#78027
Recombinant human TGF-β1	PeproTech	Cat#100-21C
ImmunoCult Human CD3/CD28 T cell Activator	STEMCELL Technologies	Cat#10990
Ficoll Paque	Bio & Sell GmbH	Cat#BS.L6115
BD Pharm Lyse	BD Biosciences	Cat#555899
Trypan blue	Thermo Fisher Scientific	Cat#15250061
eBioscience fixable viability dye eFluor 780	Thermo Fisher Scientific	Cat#65-0865-18
Fixable Viability Stain 780	BD Biosciences	Cat#565388
MeOH (Methanol), LC-MS grade	Thermo Fisher Scientific	Cat# A456212
ACN (acetonitrile), LC-MS grade	Thermo Fisher Scientific	Cat# A955212
IPA (isopropanol), LC-MS grade	Thermo Fisher Scientific	Cat# A461212
MTBE (methyl *tert*-butyl ether), LC-MS grade	Sigma-Aldrich	Cat# 34875
Chloroform, LC-MS grade	Sigma-Aldrich	Cat# 366927
NH_4F (ammonium florade), ACS reagent ≥98%	Sigma-Aldrich	Cat# 216011
Ammonia, analytical grade (28%)	VWR Chemicals	Cat# 21182.294
Acetic acid glacial	VWR Chemicals	Cat# 20104.298
L-arginine	Sigma-Aldrich	Cat#A8094-25G

(*Continued on next page*)

Continued

REAGENT or RESOURCE	SOURCE	IDENTIFIER
L-phenylalanine	Sigma-Aldrich	Cat#P5482-25G
Poly-D-Lysine	Sigma-Aldrich	Cat#P7280-5MG
SMARTPOOL ON-TARGETplus human IL4I1 siRNA	Horizon Discovery	Cat#L-008109-00-0005
ON-TARGETplus Non-targeting control pool	Horizon Discovery	Cat#D-001810-10-05
BD Horizon Fixable Viability Stain 780	BD Biosciences	Cat#565388
Acetone	Sigma-Aldrich	Cat#32201-1L
4X Laemlli buffer	Bio-Rad Laboratories	Cat#1610747
β-mercatopethanol	Sigma-Aldrich	Cat#63689-100ML
Precision Plus Protein Dual Color Standards	Bio-Rad Laboratories	Cat# #1610374
WesternBright Quantum - HRP Substrate	Advansta Corporation	Cat#K-12042-D10
Restore PLUS Western Blot Stripping buffer	Thermo Fisher Scientific	Cat#46430
KYT0353	Tocris Bioscience	Cat#5026
RIPA Lysis and Extraction buffer	Thermo Fisher Scientific	Cat#89901
Pierce Protease and Phosphatase inhibitor	Thermo Fisher Scientific	Cat#A32959
Critical commercial assays		
Naive CD4+T cell isolation Kit II, human	Miltenyi Biotec	Cat#130-094-131
eBioscience^{TM} Foxp3/Transcription Factor Staining Buffer Set	Thermo Fisher Scientific	Cat#00-5523-00
CD4+T cell Isolation Kit, human	Miltenyi Biotec	Cat#130-096-533
Memory CD4+T cell Isolation Kit, human	Miltenyi Biotec	Cat#130-091-893
Seahorse Glycolytic Rate Assay kit	Agilent Technologies	Cat#103344-100
Seahorse Mito-stress Assay kit	Agilent Technologies	Cat#103015-100
Seahorse ATP-Rate Assay kit	Agilent Technologies	Cat#103592-100
Olink Target 96 Immuno-Oncology panel	Olink	Cat#95311
Olink Target 96 Cardiovascular II panel	Olink	Cat#95500
Olink Target 96 Cardiometabolic panel	Olink	Cat#95360
T cell Activation/Expansion Kit, human	Miltenyi Biotec	Cat#130-091-441
Pierce BCA protein quantification	Thermo Fisher Scientific	Cat#23227
RNeasy Mini Kit	QIAGEN	Cat#74104
RNeasy Micro kit	QIAGEN	Cat#74004
RevertAid RT kit	Thermo Fisher Scientific	Cat#K1691
Maxima SYBR Green/ROX qPCR Master Mix (2X)	Thermo Fisher Scientific	Cat#K0223
Human T cell Nucleofector Kit	Lonza	Cat#VPA-1002
SCENITH kit	Argüello et al.[48]	N/A
Phenylalanine Assay Kit	Abcam	Cat#ab241000
CFSE (CellTrace^{TM} CFSE Cell Proliferation Kit, for flow cytometry)	Thermo Fisher Scientific	Cat#C34554
CellTrace^{TM} Violet Cell Proliferation Kit	Thermo Fisher Scientific	Cat#C34557
P3 Primary Cell 4D-Nucleofector® X Kit L	Lonza	Cat#V4XP-3024
Deposited data		
Metabolomics data	This paper	NMDR: ST003135; Table S2
Transcriptomics data	This paper	GEO: GSE291310
PEA Proteomics data	This paper	DOI: https://doi.org/10.17632/9b64psp627.1
Th2 cell transcriptomics data	Seumois G et al.[63]	GEO: GSE75011
CD3+T cell Microarray data	Pablo-Torres et al.[64]	GEO: GSE224253

(*Continued on next page*)

Continued		
REAGENT or RESOURCE	SOURCE	IDENTIFIER
Experimental models: Cell lines		
Healthy adult buffy coats (male/female)	Chur Blutspendezentrum, Switzerland	N/A
Oligonucleotides		
Please see Table S11 for primers.	N/A	N/A
Software and algorithms		
Agilent MassHunter Profinder B.10.0.2 software	Agilent Technologies	RRID:SCR_017026
CEU Mass Mediator (CEUMM)	Gil-de-la-Fuente et al.[95]	http://ceumass.eps.uspceu.es/mediator/RRID:SCR_022090
Lipid Annotator	Agilent Technologies	N/A
MS-DIAL software	N/A	RRID:SCR_023076
Agilent MassHunter Qualitative Analysis	Agilent Technologies	SCR_019081
FlowJo X v.10.7.1.	BD Biosciences	RRID:SCR_008520
FlowClean algorithm	Fletez-Brant et al.[96]	N/A
DownSample v3.3.1.	BD Biosciences	RRID:SCR_021056
FlowSOM v4.0.	BD Biosciences	RRID:SCR_016899
Cluster Profiler 1.7.4.	Yu et al. 2012[97]	N/A
Seahorse Wave Desktop Software	Agilent Technologies	RRID:SCR_024491
Prism	GraphPad	RRID:SCR_002798
GEO2R interactive web tool	NCBI	www.ncbi.nlm.nih.gov/geo/geo2r/RRID:SCR_016569
Metacore software version 23.4.71500	Clarivate	RRID:SCR_008125
Fiji 2.14.0/1.54f	NIH/Schindelin et al.[98]	RRID:SCR_002285
MetaboAnalyst 5.0	MetaboAnalyst	https://www.metaboanalyst.ca/home.xhtml RRID:SCR_015539
IMPaLA	Kamburov et al.[44]	http://impala.molgen.mpg.de/
SIMCA 16.0	Umetrics	http://umetrics.com/products/simca RRID:SCR_014688
MATLAB R2018b	Mathworks	https://es.mathworks.com/products/matlab.html RRID:SCR_001622
R 4.1.0.	R project	https://www.r-project.org/RRID:SCR_001905
R pack 'Hmisc' v4.5-0	Harrell et al.	https://hbiostat.org/R/Hmisc/
Venny 2.0	Oliveros, J.C	https://bioinfogp.cnb.csic.es/tools/venny/

EXPERIMENTAL MODELS AND STUDY PARTICIPANT DETAILS

Study participants recruitment

Cohort A

The study was approved by the ethical committee of the Hospital Universitario Reina Sofia, Cordoba, Spain (registration number: 5029). Written informed consent was signed by all participants and the study followed all the directives from the Declaration of Helsinki. Study participants were recruited out of the olive pollen season. The Inclusion criteria were: individuals above the age of 18 years, continuously living for a minimum of 10 years in the Cordoba region (one of the areas with a highest seasonal exposure to olive pollen (10.000 pollen grains/m^3)) and, in case of patients, clinically diagnosed with allergic asthma and/or allergic rhinitis for at least 2 consecutive years along with olive pollen specific IgE (sIgE) $\geq$ 0.35 KU/L. Lack of signed consent, incomplete understanding of the nature of study, pregnancy and oncological and other inflammatory diseases constituted as the exclusion criteria. Upon recruitment, the participants were divided into 3 groups, namely non allergic controls, mild, and severe allergic patients depending on their sensitization profile and accompanying symptoms[54,55] as listed.

(1) Non allergic controls (n = 10) – no sensitization to allergens and absence of symptoms.

(2) Mild allergy (n = 9) – olive pollen sensitization with IgE against Ole e 7 < 15 kU/L and mild respiratory symptoms.
(3) Severe allergy (n = 11) – olive pollen sensitization with IgE against Ole e 7 > 15 kU/L with severe respiratory symptoms during pollen season.

De-identified patients' demographics and clinical characteristics are available in Tables S12 and S13. Memory $CD4^{+}$Teff and Treg cells from this cohort were analyzed by metabolomics, lipidomics and flow cytometry, whereas serum samples were analyzed by Proximity Extension Assay (PEA) technology (Olink, Uppsala, Sweden), as described below.

Cohort B

This previously published cohort by Seumois et al., 2016[63] comprised of allergic asthma patients (*n* = 37), allergic rhinitis patients (*n* = 25) and healthy controls (*n* = 15). Patients were classified depending on Global Initiative for Asthma (GINA) and skin test reactivity to a total of 32 seasonal and perennial allergens. The study was approved by the Institutional Review Board of La Jolla Institute for Allergy and Immunology, La Jolla, California, USA. Written informed consent was provided by all participants. Participants' Th2 cells, characterized as $CD3^{+}CD4^{+}CD45RA^{-}CD25^{-}CCR4^{+}$ cells were sorted by FACS from peripheral blood mononuclear cells (PBMCs) and their RNA was isolated, amplified and sequenced by HiSeq2500 (Illumina, San Diego, CA, USA). Additional information is available in Seumois et al., 2016.[63] RNA-seq data of Th2 cells from this cohort were accessed using GEO: GSE75011 and re-analyzed by us, as described below.

Cohort C

This cohort, previously published by our group, comprised of 16 patients suffering from allergic asthma (mild *n* = 9, severe *n* = 7, classified by GINA) and 8 non-allergic healthy controls with sensitization being defined by Skin Prick Test for aeroallergens. The study was approved by the Committees of Research and Ethics from the Hospital Universitario Puerta de Hierro Majadahonda. Written informed consent was signed by all participants. RNA was isolated from participants' $CD3^{+}$T cells and subjected to transcriptomics analysis using GeneChip Human Gene 2.1 ST strips (Affymetrix, Thermo Fisher Scientific, Waltham, MA, USA) and manufacturer instructions. Further details can be found in Pablo-Torres et al., 2023[64] (GEO: GSE224253). Microarray data from $CD3^{+}$T cells from this cohort were re-analyzed by us, as described below.

Cohort D

To analyze the systemic level of L-phenylalanine, an additional cohort of olive-pollen allergic patients with the same characteristics as Cohort A was recruited. 8 non-allergic subjects and 67 allergic patients (30 classified as mild and 37 as severe, as in Cohort A) were recruited to analyze L-phenylalanine in serum. The study was approved by the ethical committee of Hospital Clínico San Carlos (date of approval: 19th December 2019; code: 19/552-E_BC). Written informed consent was signed by all participants and the study followed all the directives from the Declaration of Helsinki. Clinical characteristics of this cohort are presented in Table S27. Serum samples from this cohort were analyzed by targeted metabolomics, as described below.

Cohort E

This cohort, previously published by our group,[66] consisted of patients allergic to grass and/or birch pollen (*n* = 13) that underwent allergen immunotherapy, and non-sensitized, non-allergic healthy controls (*n* = 12) which were followed at the same time points. The study was approved by the Ethics Committee of the Canton of Bern (Switzerland). Written informed consent was signed by all participants. The baseline samples, prior to allergen immunotherapy in patients and controls (same time point), were analyzed in the current study. Their allergen sensitization status was concluded using Skin Prick Test and detection of allergen specific IgE. Clinical characteristics of this cohort are presented in Table S28. Further information pertaining to the cohort has been described in Eljaszewicz et al., 2021.[66]

Buffy coats and human primary $CD4^{+}$T cell subsets

Buffy coats were obtained from healthy human donors from Chur Blutspendezentrum (Chur, Switzerland) and stored at room temperature prior to processing. Age, gender, and ethnicity of these donors is unknown as these buffy coats were ordered and this information was not requested. Next, PBMCs were isolated using the protocols provided below. Depending on requirements, specific number of PBMCs were further processed while the excess was frozen in freezing medium (90% Heat inactivated Fetal Calf Serum (FCS, Merck, Darmstadt, Germany) + 10% DMSO (Sigma-Aldrich, St. Louis, MO, USA) and stored in liquid nitrogen until future use. Total $CD4^{+}$T, memory $CD4^{+}$T and naive $CD4^{+}$T cells were always isolated from PBMCs obtained from buffy coats of healthy donors. Total $CD4^{+}$T cells were used in Seahorse Glycolytic Rate and Mito-stress assays and assessment of SLC7A5/LAT1 expression in patient samples. Similarly, human memory $CD4^{+}$T cells were utilized in metabolomics and lipidomics, Seahorse Glycolytic Rate and Mito-stress assays, proliferation and IL4I1 knockdown studies, and flow cytometric analyses. Naive $CD4^{+}$T cells were isolated from PBMCs and subjected to the Th2 differentiation protocol provided below. Following differentiation, they were used in the RT-qPCR analyses, proliferation, Seahorse ATP-Rate assays, phenotypic characterization, and *SLC7A5*/LAT1 studies.

In vitro short differentiation of helper T cell subsets

Previously published differentiation protocols by Yang et al.[99] were used. In short, naive $CD4^{+}$T cells were isolated from PBMCs by using the Naive human $CD4^{+}$T cell isolation kit (Miltenyi Biotec, Bergisch Gladbach, Germany) on the autoMACS Pro Separator (Miltenyi Biotec, Bergisch Gladbach, Germany). Subsequently, they were transferred to appropriate differentiation media containing ImmunoCult Human CD3/CD28 T cell Activator (STEMCELL Technologies, Vancouver, Canada) (25μL/mL) and below mentioned

cytokines prepared in RPMI+10%FCS. Th0 cells – no cytokine stimulation. Th1 cells – 10ng/ml IL-12 (Peprotech, Thermo Fisher Scientific, Waltham, MA, USA) and 10μg/ml anti-IL-4 antibody (Thermo Fisher Scientific). Th2 cells – 20ng/ml IL-4 (Peprotech, Thermo Fisher Scientific, Waltham, MA, USA) and 10μg/ml anti-IFN-γ antibody (Thermo Fisher Scientific). Th17 cells – 2.5ng/ml TGF-β (Peprotech, Thermo Fisher Scientific, Waltham, MA, USA), 15ng/ml IL-6 (Peprotech, Thermo Fisher Scientific, Waltham, MA, USA), 10μg/ml anti-IFN-γ and 10μg/ml anti-IL-4 antibodies (Thermo Fisher Scientific). Treg cells – 1.5ng/ml TGF-β (Peprotech, Thermo Fisher Scientific, Waltham, MA, USA), 10μg/ml anti-IFNγ and 10μg/ml anti-IL-4 antibodies (Thermo Fisher Scientific, Waltham, MA, USA). On day 5 of differentiation, cells were harvested and tested for their respective status. Following confirmation of expression of transcription factor (Th1 – T-bet; Th2 – GATA3; Th17 – RORγt; Treg – FOXP3), cells were used in experiments (Figure S5).

In vitro long differentiation of human Th2 cells

Fresh PBMCs were isolated from buffy coats of healthy donors and resuspended in PBS containing 0.5% BSA (Sigma-Aldrich, St. Louis, MO, USA) and 2mM EDTA (Sigma-Aldrich, St. Louis, MO, USA), as stated previously. Complete RPMI (cRPMI) consists of 10% heat inactivated FCS (Merck, Darmstadt, Germany), 1X Penicillin/Streptomycin (Sigma-Aldrich, St. Louis, MO, USA), 1X Vitamins (Sigma-Aldrich, St. Louis, MO, USA), 1X Sodium pyruvate solution (Sigma-Aldrich, St. Louis, MO, USA), and 1% MEM Non-essential Amino Acid (NEA) (Sigma-Aldrich, St. Louis, MO, USA) solution prepared in RPMI1640 (Lonza, Basel, Switzerland). Naive $CD4^+$T cells were isolated from PBMCs with the autoMACS Pro Separator (Miltenyi Biotec, Bergisch Gladbach, Germany) using the Naive human $CD4^+$T cell isolation kit (Miltenyi Biotec, Bergisch Gladbach, Germany) and resuspended in cRPMI containing recombinant human IL-2 (rhIL-2, 10ng/mL, PeproTech, Thermo Fisher Scientific, Waltham, MA, USA), recombinant human IL-4 (rhIL-4, 12.5ng/mL, PeproTech, Thermo Fisher Scientific, Waltham, MA, USA), anti-IFNγ antibody (5μg/mL, Thermo Fisher Scientific), anti-IL-10 antibody (5μg/mL, Thermo Fisher Scientific) and ImmunoCult Human CD3/CD28 T cell Activator (25μL/mL, STEMCELL Technologies, Vancouver, Canada) and incubated at 37°C and 5% CO_2. Cells were maintained at a frequency of 1 million/mL and stimulated every 7 days for a period of 21 days with the activation mix and mentioned antibodies at specified concentrations. cRPMI containing rhIL-2 (10ng/mL, PeproTech, Thermo Fisher Scientific, Waltham, MA, USA), and rhIL-4 (12.5ng/mL, PeproTech, Thermo Fisher Scientific, Waltham, MA, USA) was added between stimulations as per requirement. On day 21, cells were tested for expression of classical Th2 markers, namely, GATA3, CCR4 and CRTH2 by conventional Flow Cytometry to determine their status. Panel provided as in Table S7 and FACSAria III (BD Biosciences, Franklin Lakes, NJ, USA) were used for staining and detection, respectively. Upon confirmation, cells were used for further analyses. This differentiation protocol was adapted from Cousins, Lee, Staynov, 2002.[49] The complete differentiation program is shown in Figure S7.

METHOD DETAILS

Isolation of peripheral blood mononuclear cells (PBMCs)

Whole blood was drawn from the study participants into heparin and serum in silica tubes (Vacutainer, BD Biosciences, Franklin Lakes, NJ, USA). Next, PBMCs were isolated using standard Ficoll Paque (Bio & Sell GmbH, Feucht, Germany) by density gradient centrifugation according to the manufacturer instructions. The sera were obtained by centrifugation at 2000*g* for 10min and collecting the supernatant. Sera and isolated PMBCs were immediately frozen and stored at −80°C until further analysis.

To isolate PBMCs from buffy coats, obtained buffy coats were transferred to sterile flasks and diluted 1:1 in PBS. 30mL of diluted buffy coat was subjected to density gradient centrifugation using Ficoll Paque (Bio & Sell GmbH, Feucht, Germany) and washing steps with cold PBS containing 2mM EDTA. Subsequently, 1X BD Pharm Lyse lysing solution (BD Biosciences, Franklin Lakes, NJ, USA) was used for red blood cell lysis according to manufacturer instructions. PBMCs were washed and resuspended in PBS containing 2mM EDTA for determining counts using trypan blue (Thermo Fisher Scientific, Waltham, MA, USA) and LUNA-II Automated Cell Counter (Logos Biosystems, Gyeonggi-do, South Korea) prior to subsequent processing.

Flow cytometry and fluorescence-activated cell sorting (FACS)

To confirm the status of *in vitro* differentiated Th1, Th2, Th17 and Treg subsets, we harvested the cells on day 5 of differentiation and tested for the expression of subset specific transcription factors. In detail, harvested cells were washed with PBS and stained for viability with fixable viability stain 780 (FV780, 1:1000, BD Biosciences) for 15min in the dark at room temperature. Next, surface staining was carried out with BV510 conjugated anti-CD3 (1:100) and BUV737 conjugated anti-CD4 (1:100) antibodies. Antibody staining was done for 30min at 4°C in the dark. Following staining, cells were washed once and subjected to fixation, permeabilization and staining using the eBioscience Foxp3/Transcription Factor Staining Buffer Set (Thermo Fisher Scientific, Waltham, MA, USA) according to manufacturer instructions. Cells were washed and stained with fluorochrome conjugated antibodies specific for a given subset (Th1 – RY703-*anti*-T-bet; Th2 – BUV395-*anti*-GATA3; Th17 – PE-CF594-*anti*-RORgT; Treg – RB705-*anti*-FOXP3; 1:100). Intracellular antibody staining was done for 30-45min at 4°C. Post staining, cells were washed as per kit instructions and resuspended in PBS. After sample preparation, data was acquired on the BD LSRFortessa Cell Analyzer and analyzed with FlowJo v10.

To assess the expression of IL4, *in vitro* long differentiated Th2 cells were incubated in media additionally supplemented with 1mM Phe or Veh at a concentration of 1million cells/mL of media. Cells were simultaneously stimulated with anti-CD2, anti-CD3 and anti-CD28 antibody coated beads. Cells were incubated at 37°C, 5% CO_2 for 48h. Post incubation, cells were harvested and washed with PBS. Subsequently, cells were stained with Fixable Viability stain 780 (1:1000, BD Biosciences, Franklin Lakes, NJ, USA) for 15min in

the dark. Next, cells were stained with BV510 conjugated anti-CD3 and BUV737 conjugated anti-CD4 antibodies as part of surface staining. Cells were then fixed and permeabilized using the eBioscience Foxp3/Transcription Factor Staining Buffer Set (Thermo Fisher Scientific, Waltham, MA, USA) and stained with BUV395 conjugated anti-GATA3, and BV421 conjugated anti-IL4 antibodies as per manufacturer instructions. Cells were washed and data was acquired on the BD LSRFortessa Cell Analyzer and analyzed with FlowJo v10.

For sorting, frozen PBMCs were thawed, washed with warm RPMI1640 medium (Lonza, Basel, Switzerland) and incubated on a gentle roller for 2h at 37°C and 5% CO_2. Following incubation, cells were washed and stained with eBioscience fixable viability dye eFluor 780 (Thermo Fisher Scientific, Waltham, MA, USA) (1:1000) in the dark at 4°C for 20min. After washing, cells were stained with the antibody panel for T and ILC cells for 30min at room temperature (RT) in the dark. The complete panel of antibodies and isotype controls is presented in Table S1. Cells were then sorted using BD FACSAria III (BD Biosciences, Franklin Lakes, NJ, USA) as follows: Memory $CD4^+$Teff cells: $Lineage^-$, $CD3^+$, $CD4^+$, $CD8^-$, $CD45RA^-$, $CD127^+$, $CD25^-$; and Memory Treg cells: $Lineage^-$, $CD3^+$, $CD4^+$, $CD8^-$, $CD45RA^-$, $CD127^-$, $CD25^+$. Gating strategy used for sorting and subsequent analyses is shown in Figure S1. For metabolomics and lipidomics, cells were sorted to serum free RPMI medium at 4°C. Cells were centrifuged to discard media following which they were immediately fixed with HPLC grade Methanol (MeOH) (Thermo Fisher Scientific, Waltham, MA, USA), following centrifugation at 370*g* for 5min, and frozen at −80°C until metabolomics analysis was performed. Samples collected from healthy controls (n = 6) and allergic patients (n = 11) underwent T cell sorting and metabolomics analysis. Samples from additionally recruited participants were analyzed in the same settings, equipment and protocol as described above.

Flow cytometry data analyses

Flow cytometry data were analyzed with classical gating (Figures S1, S3, S5, S13, S14, S17) and two-dimensional reduction using t-distributed stochastic neighbor embedding (tSNE) algorithms using FlowJo X v.10.7.1. (BD Biosciences, Franklin Lakes, NJ, USA) (Figures S17). First, the data were cleaned by using the FlowClean algorithm.[96] Next, classical gating was applied for T cell populations and ILCs (for representative gating strategy, please see Figure S13). For two-dimensional reduction, memory $CD4^+$Teff cells ($CD3^+CD4^+CD45RA^-$ $CD127^{++}CD25^-$) were gated and downsampled using DownSample v3.3.1. to ensure that analyses are performed with the same number of events, namely, the highest event number in the available gate. Next, all files were concatenated into a new data file (separate for allergic individuals and controls). Finally, the FlowJo tSNE algorithm[100] was applied (Iterations: 1000 Perplexity: 30 Eta: 9100 KNN: Exact, Gradient algorithm: Barnes-Hut) and analyzed with FlowSOM v4.0.[101] and clusterProfiler[97] 1.7.4. based on CD161, CRTH2, cKit, CD127, CD25 and PD1 expression. Differential clustering was successfully generated mostly based on expression of CD161 and PD1 whereas cKit and CRTH2 did not generate any separate clusters due to low counts of positive cells post down sampling and relative heterogeneity (Figure S17). The populations obtained with FlowSOM were back-gated in the original files to obtain the total frequency of the population of interest in each sample.

Metabolomics

Sample preparation

As previously published,[102,103] frozen cells in MeOH were thawed at 4°C. Then, a first extraction targeted at lipid metabolites was performed by adding Methyl *tert*-butyl ether (MTBE) (Sigma-Aldrich, St. Louis, MO, USA) to a final concentration of MTBE:MeOH (1:4). Samples were then sonicated for a total of 15min at 15W (3 rounds of 5min sonication), thoroughly vortexed, and centrifuged for 10min at 16000g to remove any debris. The supernatant was collected. Then a second extraction took place to obtain the most polar metabolites in the sample. To that extent, 100μL of a mixture of H_2O:MeOH (1:4) was added and the sample was sonicated again for a total of 15min at 15W (3 rounds of 5min sonication), thoroughly vortexed and centrifuged for 10min at 16000g to remove any debris. The supernatant was collected. Polar samples were kept at 4°C until analysis. Extraction solvents were used as a blank and followed the same procedure as the samples. Both, memory $CD4^+$T effector and regulatory T cells were analyzed as one batch suggesting real biological differences.

Quality control (QC) samples were prepared by pooling equal volumes of samples from the same cell type separately. Then, the QC was aliquoted into different tubes to preserve them from freezing-thawing cycles. Every time a new QC was needed, a fresh QC aliquot was extracted. The QCs clean-up steps followed the same procedure applied to the experimental samples. QCs were analyzed throughout the run to provide a measurement of system stability, performance, and reproducibility of the system.

Additionally, to select features produced only by cells, a calibration curve was made of Teff or Treg cells, respectively. The curve consisted of 4 points that were prepared by seriated dilutions. The number of cells for each point of the Teff cell curve were: 100000, 250000, 500000, and 1500000 cells. For the Treg cells, they were: 10000, 25000, 50000 and 100000 cells. Each cell curve was measured at the beginning, in the middle, and at the end of the analysis of each experimental sample set.

Samples were divided into 2 groups depending on the type of subpopulation they belong to either Teff or Treg. Samples were randomized in 2 consecutive injection sets, analyzing first Treg followed by Teff samples. All samples were measured in duplicate.

Analysis by RP-UHPLC-ESI-QTOF-MS analysis

Cell samples were measured using an Agilent HPLC system (1200 series) coupled with quadrupole-time of flight analyzer system (Q-ToF MS 6545) (Agilent Technologies, Waldbronn, Germany).

Lipidomics. Data from cell samples were acquired using an Agilent 1290 Infinity II UHPLC system coupled to an Agilent 6550 quadrupole time-of-flight (QTOF) mass spectrometer. The Agilent 1290 Infinity II Multisampler system, equipped with a multi-wash option, was used to uptake 1 and 2μL of extracted samples in positive and negative ionization modes, respectively. The multisampler temperature was maintained at 15°C to preserve lipids in a stable environment and avoid precipitation. An Agilent InfinityLab Poroshell 120 EC C18 (3.0 × 100mm, 2.7μm) (Agilent Technologies, Waldbronn, Germany) column and a compatible guard column (Agilent InfinityLab Poroshell 120 EC C18, 3.0 × 5mm, 2.7μm) were used and maintained at 50°C. The chromatography gradient started at 70% of B at 0 – 1min, 86% at 3.5–10min, 100% B at 11–17min. The starting conditions were recovered by minute 17, followed by a 2min re-equilibration time; the total running time was 19min. The mobile phases used for both positive and negative ionization modes consisted of (A) 10mM ammonium acetate, 0.2mM ammonium fluoride in 9:1 water/methanol and (B) 10mM ammonium acetate, 0.2mM ammonium fluoride in 2:3:5 acetonitrile/methanol/isopropanol. The flow rate was held constant, set at 0.6mL/min. The multi-wash strategy consisted of a mixture of methanol:isopropanol (50:50, v/v) with the wash time set at 15s, and aqueous phase:organic phase (30:70, v/v) mixture to assist in the starting conditions.

The Agilent 6550 QTOF mass spectrometer equipped with a dual AJS ESI ion source was set with the following parameters: 150V fragmentor, 65V skimmer, 3500V capillary voltage, 750V octopole radio frequency voltage, 10L/min nebulizer gas flow, 200°C gas temperature, 50psi nebulizer gas pressure, 12L/min sheath gas flow, and 300°C sheath gas temperature. Data were collected in centroid in positive and negative ESI modes in separate runs, operated in full scan mode from 50 to 1700*m/z* with a scan rate of 3 spectra/s. A solution consisting of two reference mass compounds was used throughout the whole analysis: purine ($C_5H_4N_4$) at *m/z* 121.0509 for the positive and *m/z* 119.0363 for the negative ionization modes; and HP-0921 ($C_{18}H_{18}O_6N_3P_3F_{24}$) at *m/z* 922.0098 for the positive and *m/z* 980.0163 (HP-0921+acetate) for the negative ionization modes. These masses were continuously infused into the system through an Agilent 1260 Iso Pump at a 1mL/min (split ratio 1:100) to provide a constant mass correction. Ten Iterative-MS/MS runs were performed for both ion modes at the end of the analytical run. They were operated with an MS and MS/MS scan rates of 3spectra/s, 40–1700*m/z* mass window, a narrow (~1.3amu) MS/MS isolation width, 3 precursors per cycle, 5000 counts, and 0.001% of MS/MS threshold. Five iterative-MS/MS runs were set with a collision energy of 20eV, and the subsequent five runs were performed at 40eV. References masses and contaminants detected in blank samples were excluded from the analysis to avoid inclusion in the iterative-MS/MS.

Additionally, confirmation of some compounds was performed by LC-MS/MS experiments using 20V for fragmentation.

Polar extraction metabolomics. The HPLC system was equipped with a degasser, two binary pumps, and a thermostated autosampler. Briefly, 2μL of the sample were injected into an Agilent Zorbax Extend C18 (2.1 × 50mm, 1.8μm; Agilent, Waldbronn, Germany), with a guard column Zorbax Extend C18 (3 × 5mm, 1.8μm; Agilent, Waldbronn, Germany), both maintained at 60°C. The flow rate was set at 0.6mL/min. The elution gradient involved a mobile phase consisting of: (A) water containing 0.1% of formic acid and (B) acetonitrile containing 0.1% of formic acid. The initial conditions were set at 5% phase B for 1min, which increased linearly to 80% phase B in 7min. Then in 4.5min it increased until 100% of phase. Then the equipment returned to the initial condition in 0.5min, which was held for 3min for column reconditioning. Samples were analyzed in both ESI+ and ESI- modes in separate injections. The capillary voltage was set at 3000 for both polarities. The drying gas flow rate was 12L/min at 250°C and gas nebulizer at 52psi; fragmentor voltage was set at 175V in ESI+ and 250 in ESI-; skimmer and octupole radio frequency voltages were set to 65 and 750V, respectively. MS spectra were collected in the centroid mode at a scan rate of 3spectra/s. The MS detection window was performed in a full scan from 100 to 1200*m/z* for both modes. Automatic MS recalibration during batch analysis was carried out by introducing a reference standard into the source via a reference sprayer valve. Reference masses for ESI+ were purine (*m/z* = 121.0508) and HP-0921 (*m/z* = 922.0097), whereas for ESI− TFA NH_4 (*m/z* = 112.9855) and HP-0921 (*m/z* = 966.0007).

Data processing

Memory $CD4^+$Teff and Treg raw data were re-processed together so they could be compared, as each feature has an average mass and an average retention time (unique ID in both cell types). The data collected after the LC-MS analyses in both positive and negative ion modes were reprocessed with the Agilent MassHunter Profinder B.10.0.2 software. The datasets were extracted using the Batch Recursive Feature Extraction (RFE) workflow integrated into the software. This workflow comprises two steps: the Batch Molecular Feature Extraction (MFE) and the Batch Find by Ion Feature extraction (FbI). The MFE algorithm consists in removing unwanted information, including the background noise, and then creating a list of possible components that represent the full range of TOF mass spectral data features, which are the sum of coeluting ions that are related by charge-state envelope, isotope pattern, and/or the presence of different adducts and dimers. Additionally, in the case of lipidomics, the MFE is intended to detect coeluting adducts of the same feature, selecting the following adducts: $[M + H]^+$, $[M+Na]^+$, $[M + K]^+$, $[M + NH4]^+$ and $[M + C2H6N2+H]^+$ in LC-MS positive ionization; $[M-H]^-$, $[M + CH3COOH-H]^-$, and $[M + Cl]^-$ in LC-MS negative ion mode. Whereas, in polar analysis, the following adducts: $[M + H]^+$, $[M+Na]^+$, $[M + K]^+$, and $[M + NH4]^+$ in LC-MS positive ionization; $[M-H]^-$, $[M+HCOOH-H]^-$, and $[M + Cl]^-$ in LC-MS negative were selected. The neutral loss (NL) of water is also considered for both ion modes in the two methods (lipidomics and polar analysis). The algorithm then aligns the molecular features across the study samples using the mass and retention time (RT) to build a single spectrum for each compound group. The next step involves FbI, using the median values derived from the MFE process to perform a targeted extraction to improve the reliability of finding and reporting features from complex datasets used for differential analysis.

After data pre-processing, the quality assurance (QA) of the datasets were reprocessed independently according to their blank samples, experimental samples, QC samples, and their specific cell calibration curve. This consisted of raw data filtration by keeping all features that were present after blank subtraction and were detected in >75% of QCs and >75% in at least one sample group (A (controls), B (mild allergy), C (severe allergy)) and had Relative Standard Deviation (RSD) < 30% in the QCs. The rest of the signals were excluded from the analyses. Finally, features were kept if they had a significant correlation (ρ) with the number of cells in the curve ($|\rho| > 0.6$ and p value <0.05), leading to 109 and 190 chemical entities were obtained that passed LC-MS quality control for lipidomics and polar analysis, respectively. It is important to note that some of the metabolites that are present only in one of the cell subsets might also be present in the other, and the absence only indicates that it does not comply with the QA.

Missing values were replaced using the k-nearest neighbors (kNN) algorithm[104] using an in-house script developed in MATLAB (v.R2018b, MathWorks). Data were normalized using the number of cells obtained from the cell sorter. The quality of the analyses was tested using principal component analysis (PCA) models.[105]

Workflow for lipid annotation

The annotation process was carried out in three steps: the first one was an initial tentative identification of lipid features, based on the MS1 data, using our online tool CEU Mass Mediator (CEUMM) (http://ceumass.eps.uspceu.es/mediator/).[95,103,106] This tool for mass-based compound annotation comprises the information available in different databases (KEGG, HMDB, LIPID MAPS, Metlin, MINE, and an in-house library). This stage started with the tentative assignment based on (i) accurate mass (maximum mass error tolerance 20 ppm); (ii) RT; (iii) isotopic pattern distribution; (iv) the possibility of cation and anion formation; and (v) adduct formation pattern.

Secondly, to increase the level of annotation confidence, the raw LC-MS/MS data from lipidomics was imported to a Lipid Annotator software (Agilent Technologies, Waldbronn, Germany) to build a fragmentation-based (MS/MS) library comprising the *m/z* of all the precursors identified as lipids by the software, together with their corresponding RT. The Lipid Annotator method[107] was set as follows: ion species $[M + H]^+$, $[M+Na]^+$, and $[M + NH4]^+$ for positive; and $[M\text{-}H]^-$, and $[M + CH3COOH\text{-}H]^-$ for negative ionization mode. Then, for both ion modes, the Q-Score was set at $\geq$ 50; all the lipid classes were selected, the mass deviation was established as $\leq$20ppm, fragment score threshold was fixed as $\geq$30, and the total score was set at $\geq$60. Additionally, MS-DIAL software[108] was used for lipidomics and polar analysis iteratives. For this software, analytical parameters were set as described: ion species $[M + H]^+$, $[M\text{-}H2O + H]^+$, $[M+Na]^+$, $[M + K]^+$, $[M + NH4]^+$ and $[M + C2H6N2+H]^+$ in positive ionization mode; and $[M\text{-}H]^-$, $[M\text{-}H2O\text{-}H]^-$, $[M + Cl]^-$, $[M+HCOOH\text{-}H]^-$ and $[M + CH3COOH\text{-}H]^-$ in negative mode for lipidomics. Adduct formation with formic acid was observed experimentally in this study even though the mobile phases used for LC are lacking this compound. Formic acid presence was considered due to trace amount contamination levels of formate in acetate salts of LC-MS grade, or methanol oxidation during ESI (https://lipidomicstandards.org/isomeric-overlap/). In the case of polar analysis, ion species $[M + H]^+$, $[M\text{-}H2O + H]^+$, $[M+Na]^+$, $[M + K]^+$ and $[M + NH4]^+$ in positive mode; and $[M\text{-}H]^-$, $[M\text{-}H2O\text{-}H]^-$, $[M + Cl]^-$, and $[M+HCOOH\text{-}H]^-$ in negative were selected. Regardless of the analytical method or ionization mode, the search was fixed to be performed across a mass range from 50 to 1500Da (for both MS1 and MS/MS levels), the mass deviation accepted was $\leq$0.01Da and $\leq$0.025 for MS1 and MS/MS levels respectively, and the identification score cut off was also set at $\geq$60. Metabolites identification was performed against inside MS-DIAL lipidomic database[108] and MassBank of North America (MoNA) database for polar compounds (https://mona.fiehnlab.ucdavis.edu/downloads). Finally, for targeted LC-MS/MS experiments, manual spectral interpretation[109] was carried out using the software Agilent MassHunter (version 10.0), matching the retention time and MS/MS fragmentation to the available spectral data included in MetFrag and Lipid Maps.

Metabolomics data have been deposited in National Metabolomics Data Repository (NMDR)/Metabolomics workbench[94] repository with the accession number ST003135 and they are also available in Table S2.

Targeted analysis of phenylalanine

Phenylalanine concentration was analyzed in Cohort D using an adaptation of the previously published method.[65] In brief, serum samples were prepared in a randomized order and measured in batches using dynamic molecular reaction monitoring on a liquid chromatography system (1260 Infinity II, Agilent Technologies, Waldbronn, Germany) coupled to a triple quadrupole mass spectrometer with electrospray ionization Agilent Jet Stream source, 6470 Agilent Technologies.

To analyze Phenylalanine, a gradient elution on a Kinetex HILIC (150 mm $\times$ 2.1 mm $\times$ 100 Å) column maintained at 25°C was used. The mobile phases were (A) water, and (B) Acetonitrile, both with 5.5mM ammonium acetate and 0.1% acetic acid. The flow rate was 0.5mL/min, and the gradient started with 5% of A for 2min, increased up to 50% until 12min, and went back to initial conditions until 22min. The MS conditions were: 5500V of capillary voltage in positive ESI mode, a nebulizer gas flow rate of 11.0L/min, a source temperature of 250°C; and a source pressure of 60psi.

For sample preparation, 50μL of serum were mixed with 150μL of cold (−20°C) methanol: ethanol mix (MeOH:EtOH) (1:1). Then, serum samples were vortex-mixed for 20s, placed on ice for 20min and centrifuged at 16000g for 20min at 4°C. Then, 70μL of the supernatant was transferred into an LC vial and mixed with 50μL of ISTD mix][65] and 440μL of the initial conditions of the mobile phases (95% B + 5% A). QC and blank samples, prepared as described above, were also measured through the run.

Serum Protein Extension Assay (PEA) proteomics and targeted immunoassays

Protein expression in the sera of patients from Cohort A was measured using the human Protein Extension Assay technology (Olink, Uppsala, Sweden). Olink Target 96 Immuno-Oncology, Cardiovascular II and Cardiometabolic panels were used according to manufacturer's instructions. Olink data in normalized protein expression (NPX) format were imported, processed, and compared between groups with OlinkR package (https://github.com/ge11232002/OlinkR). A protein was considered expressed if it was above the detection limit in more than half of the samples in either of the two compared groups.

Hepatic growth factor (HGF) was measured following manufacturers' instruction in serum samples diluted 1:2 in PBS. The plate was analyzed on LUMINEX 200 (Luminex Corporation, Austin, TX, USA) and Bio-Plex Manager 6.1 (Bio-Rad Laboratories, Hercules, CA, USA). The data have been deposited in Mendeley data repository (doi: https://doi.org/10.17632/9b64psp627.1).

Seahorse flux analyses

For Seahorse flux analysis, fresh PMBCs were isolated from buffy coats as described previously. Next, total $CD3^+CD4^+$T cells or memory $CD3^+CD4^+$T cells were isolated using the human $CD4^+$T cell Isolation Kit or Memory $CD4^+$T cell Isolation Kit (Miltenyi Biotec, Bergisch Gladbach, Germany), for respective cell types, according to manufacturer instructions on the autoMACS Pro Separator (Miltenyi Biotec, Bergisch Gladbach, Germany). Isolated cells were then transferred to RPMI1640 medium (Lonza, Basel, Switzerland) with 10% heat inactivated FCS (Merck, Darmstadt, Germany), 1% NEA (Merck, Darmstadt, Germany) and 100U/mL rhIL-2 (PeproTech, Thermo Fisher Scientific, Waltham, MA, USA) (R10+IL2). R10+IL2 contains 1.149mM of Arg and 90.9μM of Phe in its base form. This medium was used for all assays unless stated otherwise. Cells were then incubated for 24, 48 or 72h in this culture medium additionally supplemented with either 0.1mM or 1mM Arg or Phe or Vehicle (Merck, Darmstadt, Germany). After incubation, cells where washed and their energy metabolism was assessed using Glycolytic rate and Mito stress Seahorse flux analysis assays (Agilent Technologies, Santa Clara, CA, USA) according to manufacturer instructions. Acute activation of T cells was performed directly during these assays, before the injection of any inhibitor, using anti-CD2, anti-CD3, and anti-CD28 antibody coated beads (Miltenyi Biotec, Bergisch Gladbach, Germany) at a ratio of 3:1 (beads:cells). A similar protocol was used for *in vitro* short differentiated Th1, Th2, Th17 and Treg cells and *in vitro* long differentiated Th2 cells wherein cells were incubated in appropriate culture medium for 24h, washed, and seeded in a Seahorse plate pre-coated with Poly-D-Lysine (50μg/mL) at a concentration of $1x10^5$ cells/well, after which Seahorse ATP rate assay was performed as per manufacturer instructions. Following completion of the assay, the supernatant in each well was discarded and the plate was frozen at −80°C for normalization purposes. Data normalization was done either by quantifying the total protein content using the Pierce BCA protein quantification assay (Thermo Fisher Scientific, Waltham, MA, USA) by manufacturer instructions or by cell count since they were seeded on the day of analysis. Preliminary data analysis was performed on the Seahorse Wave Desktop Software (Agilent Technologies, Santa Clara, CA, USA) following which data were exported and further analysis was done using Prism (GraphPad Software Inc., Boston, MA, USA).

Proliferation assay

Memory $CD3^+CD4^+$T cells were isolated from buffy coats as described before and cultured in R10+IL2. Cells were stained with 5μM CellTrace Violet Cell Proliferation Kit (Thermo Fisher Scientific, Waltham, MA, USA) following manufacturer instructions and seeded at a concentration of 10^6/mL in R10+IL2 supplemented additionally with 1 mM of Phe (Merck, Darmstadt, Germany) or vehicle and stimulated with anti-CD2, anti-CD3 and anti-CD28 antibody coated beads (Sigma Aldrich, St. Louis, MO, USA) and incubated at 37°C and 5% CO_2. Following 72h incubation, cells were harvested and stained with BD Horizon Fixable Viability Stain 780 (FV780; 1:1000; BD Biosciences, Franklin Lakes, NJ, USA) in the dark for 20min. Subsequently, cells were stained with FITC conjugated anti-CD45 antibody for 30min at 4°C in the dark. Cells were washed, resuspended in PBS and acquired on a BD Fortessa flow cytometer (BD Biosciences, Franklin Lakes, NJ, USA). Frequency of live cells was quantified based on negative selection in the FV780 gate post discrimination of lymphocytes and singlets (Figure S3).

Proliferation of Th2 cells was also assessed by flow cytometry. *In vitro* long differentiated Th2 cells were washed and stained with CFSE (5uM) according to manufacturer instructions (Thermo Fisher Scientific, Waltham, MA, USA). Stained Th2 cells were incubated in R10+IL2 additionally supplemented with 1mM Phe or vehicle and stimulated with/without anti-CD2, anti-CD3 and anti-CD28 antibody coated beads for 24h. Post incubation, cells were washed and stained with eBioscience viability dye eFlour 780 (1:1000; Thermo Fisher Scientific, Waltham, MA, USA) at 4°C in the dark for 20min. Cells were washed, resuspended in PBS and data was acquired on BD LSRFortessa Cell Analyzer (BD Biosciences, Franklin Lakes, NJ, USA) and analyzed using FlowJo v10.

Quantitative reverse transcription-polymerase chain reaction (RT-qPCR)

Memory $CD3^+CD4^+$T cells were isolated, cultured and treated with Phe or Vehicle as mentioned above. *In vitro* long differentiated Th2 cells were incubated in R10+IL2 supplemented with 0.05mM, 0.1mM, 0.5mM and 1mM Phe or Vehicle with or without bead-based T cell stimulation for 24h. The base medium contains approximately 1.14mM of L-arginine and 90.9μM of L-phenylalanine. The final concentration of phenylalanine in these media are 0.1454mM, 0.1909mM, 0.59mM and 1.09mM, respectively. Similarly, the final concentration following supplementation of 0.05mM, 0.1mM, 0.5mM and 1mM Arg are 1.18mM, 1.23mM, 1.63mM and 2.13mM, respectively. Cells were harvested and lysed on ice with RLT buffer (QIAGEN, Hilden, Germany) containing β-mercaptoethanol (10μL/mL of RLT buffer) (Sigma Aldrich, St. Louis, MO, USA), and stored at −80°C until analyses. RNA isolation was performed using RNeasy Micro kits (QIAGEN, Hilden, Germany) according to manufacturer's instructions. Quality and quantity of isolated RNA was assessed

by Nanodrop 2000 (Thermo Fisher Scientific, Waltham, USA). The flow-through from the RNeasy spin column was saved and stored at −80°C for protein precipitation. Reverse transcription was performed with RevertAid RT kit (Thermo Fisher Scientific, Waltham, MA, USA) and random hexamers according to the manufacturer's recommendations. Gene expression (5ng cDNA per well) was assessed by RT-qPCR using 5μM of appropriate primers and SYBR Green/ROX qPCR Master Mix (Thermo Fisher Scientific, Waltham, MA, USA), performed on QuantStudio 7 Flex Real-Time PCR System (Thermo Fisher Scientific, Waltham, MA, USA). Genes expression was normalized to either elongation factor 1α (EEFA1) or 18S rRNA and presented as relative quantification calculated with the $\Delta\Delta C_t$ formula using the Vehicle-treated cells as a calibrator. A complete list of the primers used is available in the key resources table.

IL4I1 knockdown in human memory CD4$^+$T cells and proliferation

IL4I1 knockdown was carried out using siRNA methodology and Human T cell Nucleofector Kit (Lonza, Basel, Switzerland) using manufacturer instructions. Memory CD4$^+$T cells from PBMCs of healthy donors were isolated using MACS technology as described previously and allowed to rest overnight in R10+IL2 at 37°C and 5% CO_2. On the next day, 5 million cells were washed and resuspended in 100μL of prepared nucleofection reagent, and SMARTPOOL ON-TARGETplus human IL4I1 siRNA or Negative Control siRNA (final concentration 300nM) (Horizon Discovery, Cambridge, UK) and added to the supplied cuvettes and subjected to electroporation using the U-14 program on Amaxa Nucleofector I device (Lonza, Basel, Switzerland). Following electroporation, cells were incubated in warm RPMI1640 for 4h at 37°C and 5% CO_2 and then transferred to R10+IL2 and incubated overnight at 37°C and 5% CO_2. Post incubation, 24h following siRNA treatment, cells were washed and stained with 5μM CFSE (Thermo Fisher Scientific, Waltham, MA, USA) according to manufacturer's instructions. Cells were then transferred to R10+IL2 (base medium contains 90.9μM of L-phenylalanine) supplemented with 1mM Phe or Vehicle with or without anti-CD2, anti-CD3 and anti-CD28 antibody coated bead (Merck, Darmstadt, Germany) (1:1 cells:beads). 24h and 48h after incubation in supplemented medium, cells were harvested and stained with BD Horizon Fixable Viability Stain 780 (1:1000) (BD Biosciences, Franklin Lakes, NJ, USA) for 15min at RT in the dark following which they were stained with BV510 conjugated anti-CD45 antibody (1:100, clone H130, BioLegend, San Diego, CA, USA) and incubated for 30min at 4°C in the dark. Cells were then washed, and data was acquired on BD FACS Aria III (BD Biosciences, Franklin Lakes, NJ, USA).

IL4I1 knockdown in human *in vitro* short-differentiated T helper cell subsets and proliferation

In vitro short-differentiated helper T cells (Th1, Th2, Th17 and Treg) from 3 donors were obtained using the protocol mentioned previously. Cells were electroporated using the 4D-Nucleofector system and P3 Primary Cell 4D-Nucleofector X Kit L (Lonza) as per manufacturer instructions and rested temporarily in serum-free RPMI at 37°C and 5% CO_2 for 4 h. Subsequently, they were transferred to R10+IL2 and rested overnight. On the next day, Th cell subsets from each donor were labeled using the CellTrace Violet Cell Proliferation Kit (5μM, Thermo Fisher Scientific, Waltham, MA, USA) following manufacturer instructions and incubated in media additionally supplemented with 1mM Phe along with anti-CD2, anti-CD3 and anti-CD28 antibody coated bead (Merck, Darmstadt, Germany) stimulation for 48h. Subsequently, they were harvested, washed and stained for viability using the Fixable Viability stain 780 (1:1000, BD Biosciences, Franklin Lakes, NJ, USA) for 15min in the dark at room temperature. Next, cells were washed and stained with FITC conjugated anti-CD45 antibody (1:100) for 30min in the dark at 4°C. After processing for flow cytometry, samples were washed and acquired on BD LSRFortessa Cell Analyzer and analyzed using FlowJo v10.

SCENITH: Single cell ENergetIc metabolism by profilIng translation inHibition

SCENITH is a flow cytometry-based technique used to assess cellular metabolism, developed by Arguello et al., 2020.[48] Frozen PBMCs were thawed, resuspended in RPMI1640 containing 10% FBS and 1X NEA and allowed to recover for a minimum of 2h. After incubation, PBMCs were washed and added to R10+IL2 supplemented with 1mM Phe or Vehicle, with or without simultaneous activation with anti-CD2, anti-CD3 and anti-CD28 antibody coated beads (1 cells:1 beads), and incubated for 12-14h. Post this incubation, cells were harvested and subjected to the SCENITH protocol.[48] In short, cells were treated with Control, 2-DG, Oligomycin, 2-DG and Oligomycin and Harringtonine inhibitors at recommended dilution for 15min at 37°C. Puromycin was added following incubation to block protein translation and incubated at 37°C for 30min. Following incubation, cells were first washed and stained with Fixable Viability stain 780 (1:1000, BD Biosciences, Franklin Lakes, NJ, USA) for 15min in the dark. After incubation, they were stained with a panel of antibodies specific for Th2 and Treg surface markers (Table S9) for 30min at 4°C. Cells were washed subjected to intranuclear staining using the FOXP3 intranuclear staining kit (Thermo Fisher Scientific, Waltham, MA, USA) to detect levels of puromycin incorporation along with intranuclear flow antibodies. Samples were then washed and read on the BD FACS Aria III and analyzed according to formulas mentioned in the original publication.[48] For *ex vivo* analysis of helper T cell subsets, total CD4$^+$T cells were isolated from 3 donors using the CD4$^+$T cell Isolation Kit, human (Miltenyi Biotec) on the autoMACS Pro Separator from freshly thawed and rested PBMCs. Cells were rested overnight. On the next day, they were transferred to Veh or 1mM Phe supplemented R10+IL2 and incubated for 12–16 h. Following incubation, cells were subjected to the SCENITH protocol as mentioned above and summarised in Figure 4A using the panel listed in Table S8.

RNA sequencing sample preparation and analysis

One million *in vitro* long differentiated Th2 cells from 5 different healthy donors were incubated for 24h in R10+IL2 additionally supplemented with 1mM of Phe or Veh with or without concurrent anti-CD2, anti-CD3 and anti-CD28 antibody coated bead (1:1) based

activation. Cells were subsequently harvested, lysed in RLT buffer containing 10μL/mL of beta-mercaptoethanol, and stored at −80°C until further processing. Total RNA was isolated using RNEasy micro kit and the RNA integrity (RIN) score was determined using the High Sensitivity RNA ScreenTape Analysis (Agilent Technologies, Santa Clara, United States) on the 4200 TapeStation System (Agilent Technologies, Santa Clara, United States) using manufacturer instructions. All samples had a RIN score of 9.8 or higher. Following the assessment of RNA quality, library preparation was performed at the Functional Genomics Center Zurich using the Illumina Stranded mRNA Prep Ligation library protocol (Illumina Inc, San Diego, Unite States) and high throughput sequencing was done on the Illumina NovaSeq X Plus. RNA sequencing analysis was performed using the SUSHI framework,[110] which encompassed the following steps: Read quality was inspected using FastQC,[111] and sequencing adaptors removed using fastp[112]; expression data were generated using kallisto[113] with the GENCODE human genome build GRCh38 (Release 42) as the reference.[114]; differential expression using the generalised linear model as implemented by the DESeq2 Bioconductor R package.[115] All R functions were executed on R version 4.4.2 and Bioconductor version 3.20. The threshold for a gene to be considered significantly expressed was set to Raw *p*-value <0.05. Gene Ontology Enrichment was done by selecting down and up regulated genes based on the raw *p*-value filtering ($p < 0.05$). The selected genes were then submitted to STRING, and GO enrichment was done using standard parameters set in STRING. Relevant pathways have been included in Figures 5G and 5H.

RNA-seq and microarray data analysis of previously published datasets

Previously published RNA-seq data of Th2 cells and microarray data of total $CD3^+$T cells were downloaded from the publicly available Gene Expression Omnibus platform under accession numbers GEO: GSE75011 (Cohort B) and GSE224253 (Cohort C), respectively. Data were analyzed with the GEO2R interactive web tool available here: www.ncbi.nlm.nih.gov/geo/geo2r/. Enrichment analysis of the most significant process networks in Th2 cells from patients with allergic asthma and allergic rhinitis, when compared to Th2 cells of non-allergic individuals, was performed with Metacore software version 23.4.71500 (Thomson Reuters, Toronto, Canada) using enrichment by GO Processess (all significantly changed genes) and by Metabolic Networks (significantly upregulated and significantly downregulated genes) based on *p* - value<0.05 (Tables S20, S21, S22, S23, S24 and S25). The phenylalanine pathway gene set was curated and adapted from GSEA and MSigDB Database (Broad Institute, MIT and Reagent of the University of California, USA) (Table S26) available under systematic names: M29207, M37685, M46134, M16650, M27835, M27850, M45537. Only genes present in the Th2 RNA-seq dataset analysis were shown in Figures 7D and S18D. The same genes were followed in our previously published $CD3^+$T cell microarray dataset and are shown in Figures S18E and S18F.

Western blotting

The flow through from the RNeasy spin column during RNA isolation, saved and stored at −80°C, was thawed and processed according to the manufacturer's instructions to obtain total protein. Briefly, 4 volumes of ice-cold acetone (Sigma-Aldrich, St. Louis, MO, USA) was added to 1 volume of flow through and incubated at −20°C for 30min. After incubation, the mix was centrifuged at maximum speed for 10min at 4°C in a benchtop ultracentrifuge and the supernatant was discarded. The pellet was washed with 100μL of ice-cold ethanol, centrifuged, and allowed to air dry after the supernatant was discarded. Then, the pellet was resuspended in 45μL 1X Laemlli buffer (270μL of 4X Laemmli buffer, 30μL of β-mercatopethanol, 900μL of water) and heated at 95°C for 5min. After heating, the samples were cooled and loaded onto a SurePAGE Bis-Tris 4-12% gel (GenScript, Piscataway, NJ, USA) with Protein Precision Plus dual color ladder (Bio-Rad laboratories, Hercules, CA, USA) and subjected to electrophoresis at 100V. Then, by means of the eBlot L1 Protein Transfer System (Genscript, Piscataway, NJ, USA), proteins were transferred from the gel onto a prepared WesternBright NC nitrocellulose membrane (Advansta Inc., San Jose, CA, USA) as per supplier instructions. The membrane was washed and blocked for 2h at room temperature with buffer containing 5% milk, and 0.05% Tween 20 prepared in PBS. Following blocking, we probed the membrane with either Rabbit anti-LAT1 (1:1000, Cell Signaling Technologies, Danvers, MA, USA) or Rabbit anti-IL4I1 antibody (1:1000, Thermo Fisher Scientific, Waltham, MA, USA) prepared in blocking buffer and incubated it overnight at 4°C. After incubation, the membrane was washed with PBS containing 0.05% Tween 20 (PBS-T) and probed with HRP conjugated goat anti-rabbit IgG (H + L) antibody (1:10000, Jackson Laboratories, Bar Harbor, ME, USA) for 1h at RT. The membrane was washed and developed with WesternBright Quantum reagent (Advansta Corporation, Menlo Park, CA, USA) per manufacturer instructions and read on Fusion FX 7 imaging system (Vilber Lourmat, Eberhardzell, Germany). The membrane was stripped with Restore PLUS Western Blot Stripping buffer (Thermo Fisher Scientific, Waltham, MA, USA), washed, blocked and probed with anti-human beta actin antibody (1:1000, Thermo Fisher Scientific, Waltham, MA, USA) and processed with the same steps. Relative protein expression of LAT1 and IL4I1 were calculated based on their intensity levels determined using Fiji[98] 2.14.0/1.54f (NIH, Bethesda, MD, USA) using beta-actin as a calibrator.

To assess the expression of phosphorylated and total mTOR and STAT6, *in vitro* differentiated Th2 cells were transferred to R10+IL2 supplemented with Veh or 1mM Phe with/without bead based CD2, CD3, and CD28 activation antibodies. At time points, 1min, 3min, 5min, 10min, 15min, 30min, 60min, 6h and 24h, cells were harvested and centrifuged at 370g. The supernatant was discarded, and cells were immediately lysed in RIPA Lysis and Extraction Buffer (Thermo Fisher Scientific, Waltham, MA, USA) containing Pierce Protease and Phosphatase Inhibitor (Thermo Fisher Scientific, Waltham, MA, USA). Lysed cells were immediately frozen and stored at −80°C until analysis. Western blotting was carried out as stated previously. The primary antibodies used are Rabbit anti-phospho-mTOR mAb (Ser2448, D9C2), Rabbit anti-mTOR mAb (7C10), and Rabbit anti-phospho-STAT6 mAb (Tyr641), and

Rabbit anti-STAT6 mAb (D3H4). Relative protein expression of phosphorylated and total transcription factors was calculated based on their intensity levels determined using Fiji 2.14.0/1.54f (NIH, Bethesda, MD, USA) and β-actin as a calibrator.

Phenylalanine quantification in *in vitro* experiments

In vitro differentiated Th2 cells were transferred to R10+IL2 supplemented with additional 1mM Phe or Vehicle. Importantly, 10μM of LAT1 inhibitor KYT0353 (Tocris Bioscience, Avonmouth, UK) was also added for selective inhibition and incubated for 6h. Upon completion of incubation, cells were harvested and immediately lysed in the Phenylalanine Assay buffer as per Phenylalanine Assay kit (Abcam, Cambridge, UK) instructions which was used to quantify Phe levels in cell lysates. Samples were processed according to manufacturer instructions and OD was measured at 450nm on the Mithras LB 940 plate reader (Berthold Technologies, Bad Wildbad, Germany) after incubation. Phe quantification in these samples and subsequent data analysis and calculations were carried out by following manufacturer instructions.

QUANTIFICATION AND STATISTICAL ANALYSIS

Statistical analysis

Multivariate analysis was performed using SIMCA v.16.0 (Sartorius Stedim Data Analytics). The PCA models were used to evaluate data quality and find patterns in the experimental samples. The cross-validated Orthogonal Partial Least Square Discriminant Analysis (OPLS-DA) models were performed to confirm the separation between the metabolism of memory $CD4^+$Teff and Tregs. The models were evaluated using R^2 and Q^2 parameters which are the classification and prediction capacity, respectively.

Complementary, univariate analysis was performed in MATLAB (v.R2018b, MathWorks) to obtain the significance of each feature in the study between studied groups. Thus, non-parametric Mann-Whitney U was used to determine statistical significance between pair groups, and the statistical significance was set at p - value<0.05. Additionally, for multiple comparison correction, False Discovery Rate (FDR) was performed by using Benjamini-Hochberg correction for these p - values, and statistical significance was set as well at FDR<0.05 for the adjusted p-value.[116] Bar representations were obtained in GraphPad Prism version 9.0.0.

Venny online tool (v. 2.0) was used to construct the Venn diagrams; and the MetaboAnalyst online tool (v. 5.0) was used to produce heatmaps graphs where data was logarithmic transformed and auto scaled. When hierarchical clustering was applied, Euclidean distance measure and Ward's clustering method were chosen as the clustering parameters. Moreover, enrichment pathway analysis was performed with IMPaLA pathway over-representation analysis tool. Two analyses, one for Teff cells and Treg cells each, were performed using those identified metabolites with an associated HMDB ID number found in both and in the specific cell subset (n = 48 metabolites for Teff cells and n = 51 for Treg cells). Only routes from KEGG database were considered.

For comparisons between two groups, t-tests either unpaired or paired t test, or Mann-Whitney U or Wilcoxon tests were used depending on data distribution and variable. Similarly, One-way ANOVA with Fisher's LSD, or Tukey, or Sidak's, or Dunnett correction were used for comparison of 3 or more groups depending on distribution and variable type. For all data analysis, sample sizes and appropriate analysis parameters including tests used are indicated in respective figure legends. GraphPad Prism v10.2.0 was used for analysis. Significance was concluded at $^*p < 0.05$, $^{**}p < 0.01$, $^{***}p < 0.001$, $^{****}p < 0.0001$.

Correlation analyses

For the correlation analyses, we used an in-house developed MATLAB script. Briefly, the number of cells of each memory $CD4^+$Teff population determined by unbiased flow cytometry analysis of severe patient samples was correlated with the abundance of the annotated compounds. Normality of cell counts and metabolite abundances was checked using the Kolmogórov-Smirnov-Lilliefors test. For normally-distributed data, Pearson correlation was used. Statistical significance was set at pcorr <0.05 and $|rho| \geq 0.7$.

Cell Reports Medicine, Volume *6*

Supplemental information

L-Phenylalanine is a metabolic checkpoint of human Th2 cells

Abhijeet J. Kulkarni, Juan Rodriguez-Coira, Nino Stocker, Urszula Radzikowska, Antonio J. García-Cívico, María Isabel Delgado Dolset, Nuria Contreras, Inés Jardón Parages, Vanesa Saiz Sanchez, Pilar Serrano, Elena Izquierdo, Cristina Gomez-Casado, Javier Sanchez-Solares, Carmela Pablo-Torres, David Obeso, Carmen Moreno-Aguilar, Maria Luisa Espinazo, Andrzej Eljaszewicz, Jana Koch, Katja Baerenfaller, Anja Heider, Ge Tan, Damir Zhakparov, Maria M. Escribese, Berta Ruiz-Leon, Cezmi A. Akdis, Rafael J. Argüello, Domingo Barber, Alma Villaseñor, and Milena Sokolowska

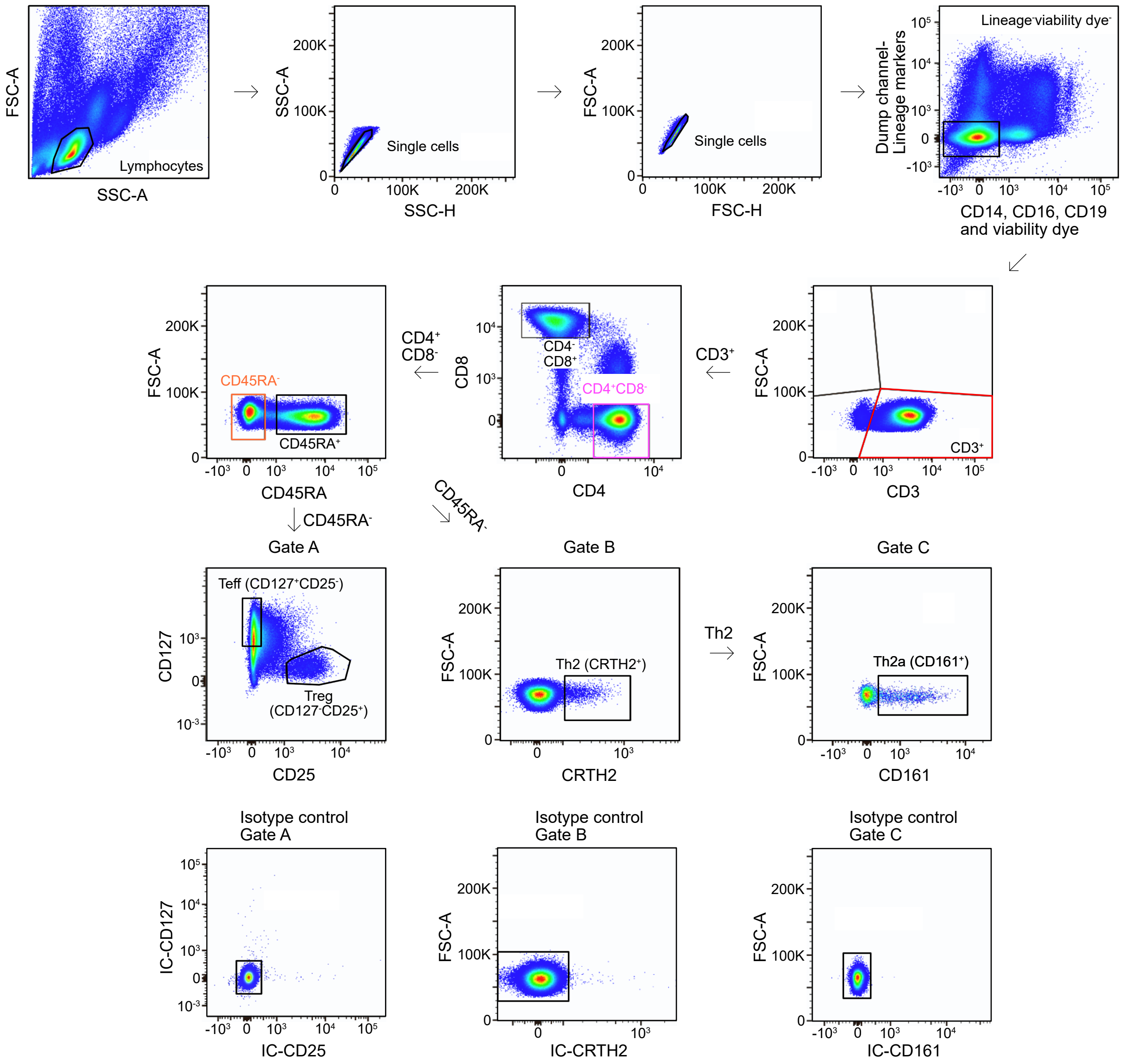

Figure S1. Gating strategy for T cells used for sorting circulating human memory CD4$^+$Teff and Treg cells and classical analysis. Related to figures 1 and 6. Briefly, T cells were gated by eliminating doublets and negatively selecting dead and other cells positive for lineage specific markers. CD3$^+$, CD4$^+$, CD8$^-$ cells were positively gated. Subsequently, memory Teff (CD45RA$^-$, CD127$^+$, CD25$^-$) and memory Treg (CD45RA$^-$, CD127$^-$, CD25$^+$) cells were sorted using gate A. Th2 and Th2a (“pathogenic”) cells were determined by the presence of CRTH2 (gate B) and double expression of CRTH2 and CD161 (gate C), respectively. Respective isotype controls are shown.

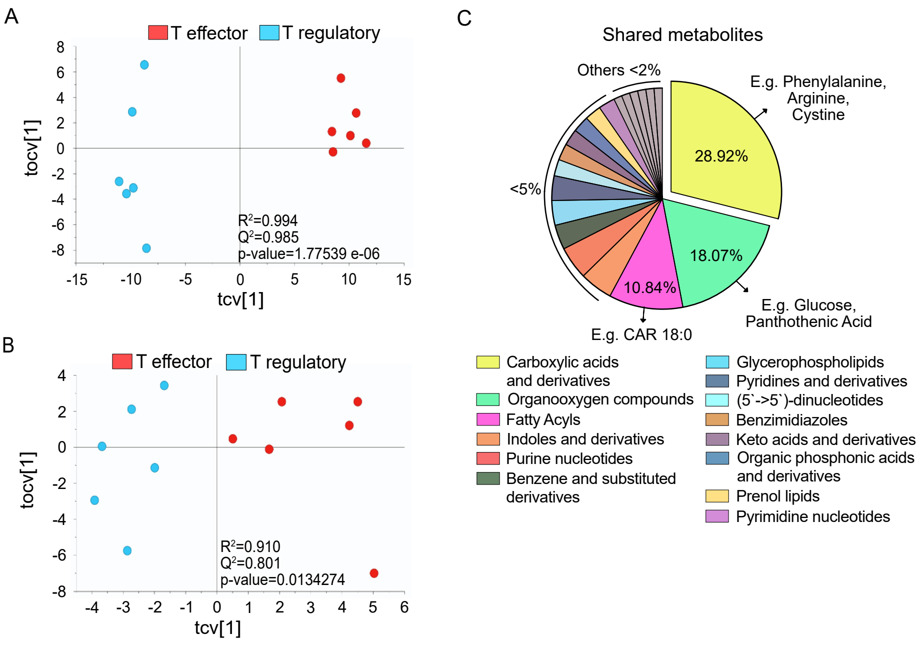

Figure S2. Unsupervised analysis of metabolomics of circulating memory CD4⁺Teff and Treg cells. Related to figure 1. (**A**) Orthogonal Partial-Least Discriminant Analysis (OPLS-DA) models revealing separation of memory CD4⁺Teff and Treg cells, based on all (shared and unique) metabolites. Data was logarithmic-transformed and pareto-scaled (log x Par). (**B**) OPLS-DA models of memory CD4⁺Teff and Treg cells revealing separation based on shared metabolites only (n=133). Data was log x Par. (**C**) Pie chart representing biochemical composition of shared metabolites in memory CD4⁺Teff and Treg cells, ordered by abundance.

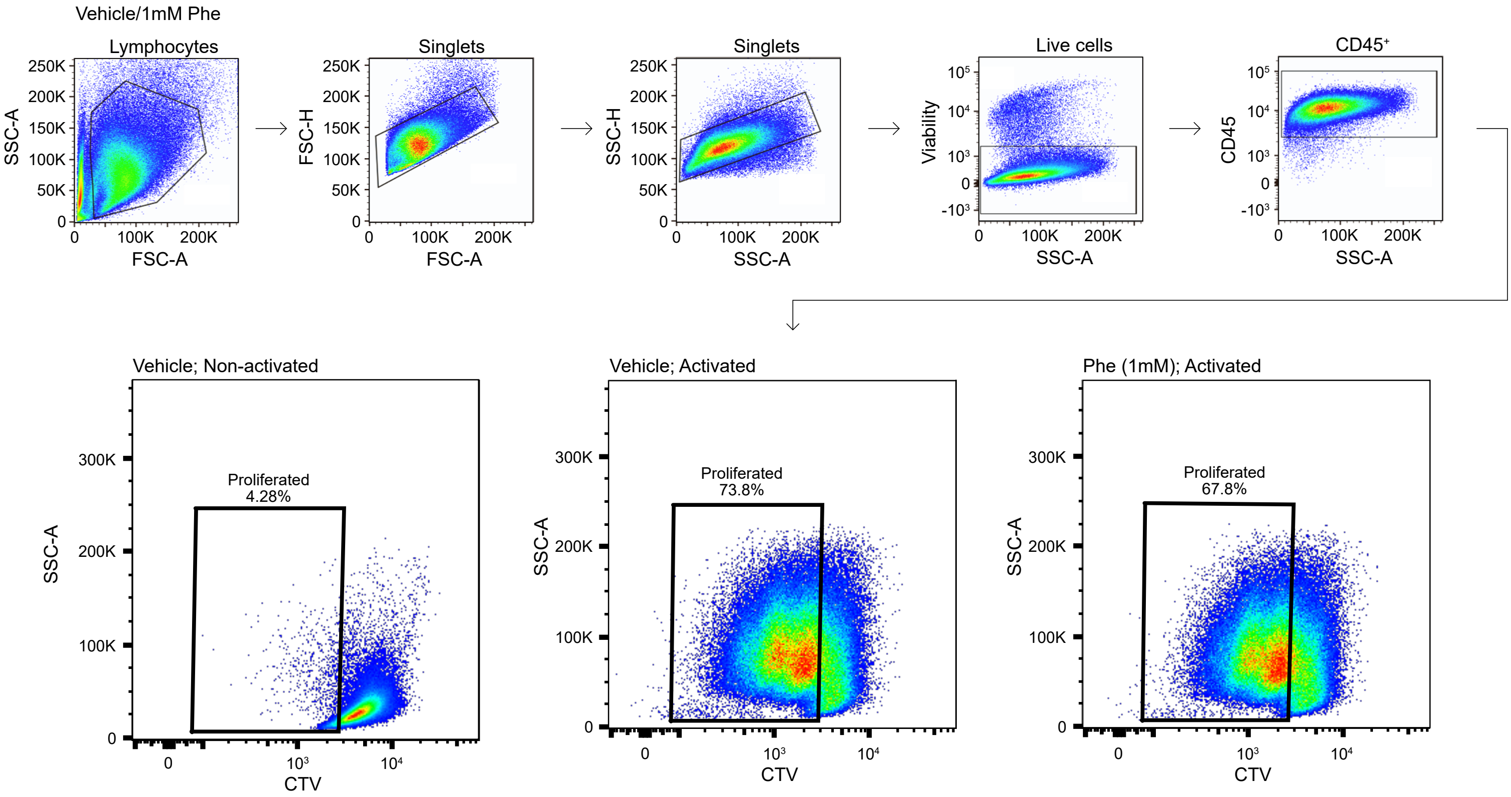

Figure S3. Gating strategy for assessment of proliferation of activated memory CD4⁺T cells. Related to figure 3. Memory CD4⁺T cells were stained with CTV and incubated in full medium (containing 90.9µM of Phe) with/without 1mM of additional Phe, with or without anti-CD2, anti-CD3 and anti-CD28 antibody coated beads for 72h. Subsequently, cells were stained for viability and CD45 expression with intermediate wash steps and data was acquired by flow cytometry. Briefly, memory CD4⁺T cells were gated by eliminating doublets and negatively selecting for live cells. Subsequently, CD45⁺ cells were positively gated. Finally, the percentage of proliferated cells was determined and CTV negative populations.

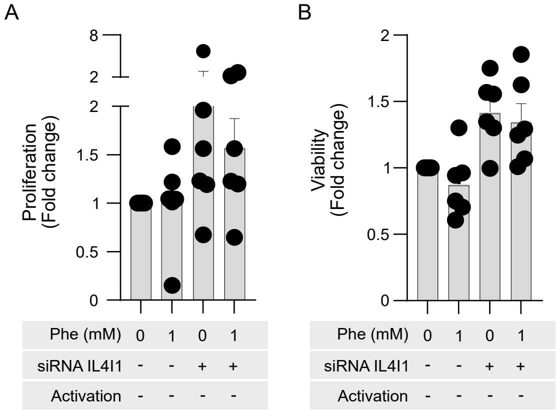

Figure S4. Proliferation of non-activated memory CD4$^+$T cells following IL4I1 knockdown. Related to figure 3.
(**A**) Quantification of proliferation of control siRNA (Crtl) and siRNA IL4I1-treated human memory CD4$^+$T cells from 2 independent experiments in 5 different donors, incubated in full medium (containing 90.9μM of Phe) with/without 1mM of additional Phe without CD2, CD3 and CD28 activation antibodies treatment for 24h prior to flow cytometry.
(**B**) Quantification of viability of siRNA Crtl and siRNA IL4I1-treated human memory CD4$^+$T cells by flow cytometry in the same experiments as in (A). Bar graphs show a fold change in proliferation as compared to the Vehicle-treated and activated cells. Wilcoxon test was used for analysis. All data are presented as mean±SEM. Ctrl, control

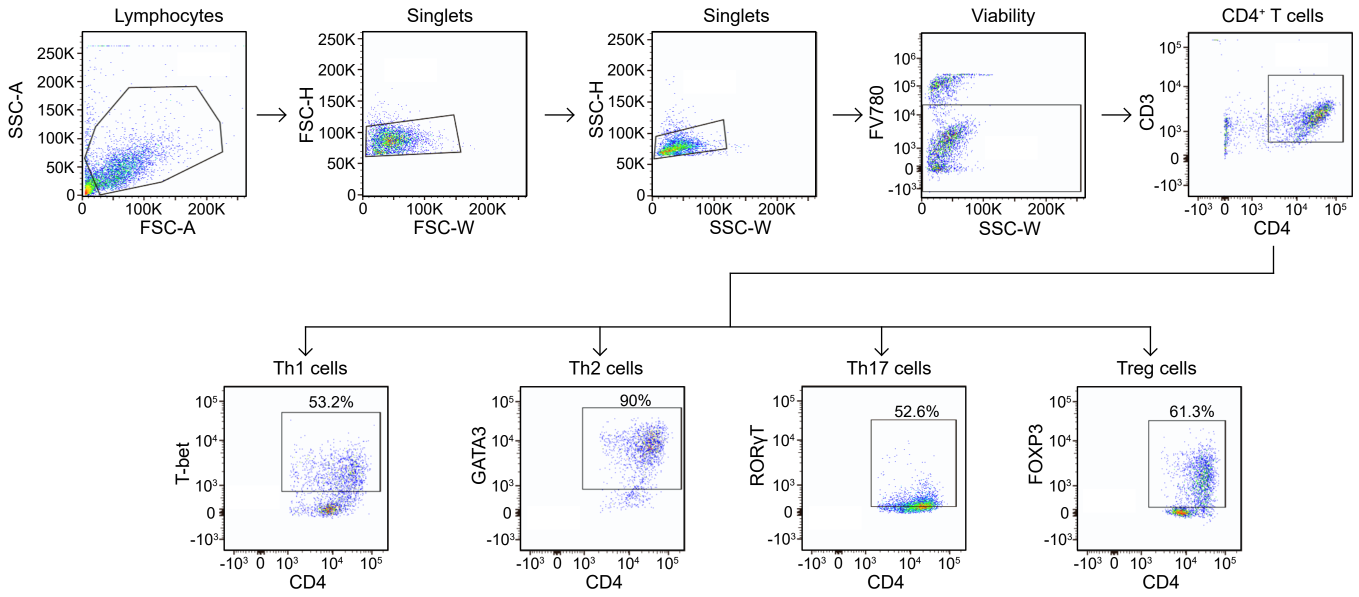

Figure S5. ***In vitro*** **short differentiated Helper T cell subsets. Related to figure 3.**
In vitro short differentiated Th1, Th2, Th17 and Treg cells were tested on day 5 of differentiation for their expression of status specific transcription factors (Th1-T-bet; Th2-GATA3; Th17- RORγT; Treg-FOXP3). Following positive selection of lymphocytes and singlets, live cells were negatively selected. $CD3^{+}CD4^{+}$ double positive cells were subsequently gated. Within this population, subset specific Transcription factor expression was used to assess differentiation.

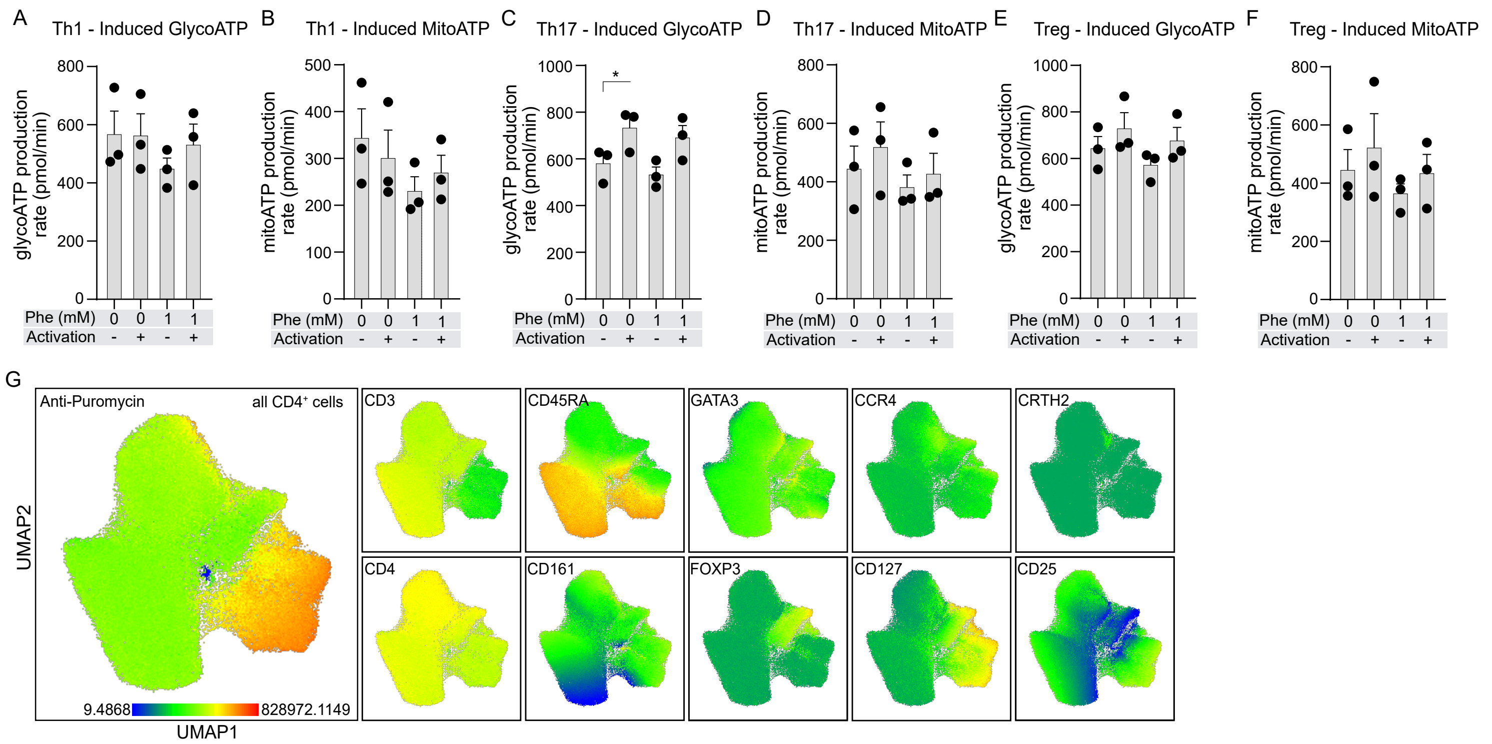

Figure S6. Energy metabolism profiling of Helper T cell subsets. Related to figure 4.
(**A-F**) GlycoATP **(A, C, E)** and MitoATP **(B, D, F)** production rates of *in vitro* short differentiated Th1, Th17, and Treg cells, respectively. Short differentiated Th cell subsets were incubated in R10+IL2 supplemented with 1mM Phe or Veh for 24h. They were subjected to Seahorse ATP Rate Assay with acute anti-CD2, anti-CD3 and anti-CD28 coated bead based activation (3:1 beads:cells). Each dot represents one subject (n=3 different subjects). Data are analysed by One-way ANOVA with Tukey correction. Bars represent mean±SEM. *p<0.05.
(**G**) Uniform Manifold Approximation and Projection (UMAP) plots of indicated protein expression in activated and non-activated $CD4^{+}$T cells. FlowJo plugin UMAP (Ver 4.0.4) was used for analysis. Frozen and thawed PBMCs were incubated in R10+IL2 supplementation of 1mM Phe or Veh with/without concurrent anti-CD2, anti-CD3, anti-CD28 antibody based activation. Subsequently, cells were harvested, washed and subjected to SCENITH depicted in Figure 4A using the panel provided in Table S9.

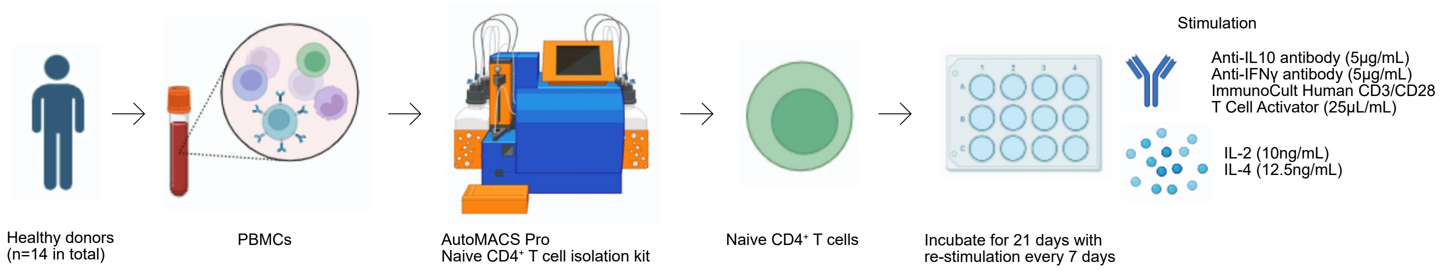

Figure S7. Methodology and timeline used for *in vitro* differentiation of naïve CD4$^+$T cells into Th2 cells. Related to figure 5 and 7. Briefly, naïve CD4$^+$T cells from PBMCs of healthy subjects are isolated and incubated in media containing IL-2 (10ng/mL), IL-4 (12.5ng/mL), anti-IFNγ antibody (5µg/mL), anti-IL-10 antibody (5µg/mL) and ImmunoCult Human CD3/CD28 T Cell Activator (25µL/mL) for 21 days with restimulation every 7 days. Further details are provided in STAR Methods. This differentiation protocol was adapted from Cousins, Lee, Staynov, 2002.[1] Prepared with Biorender.com.

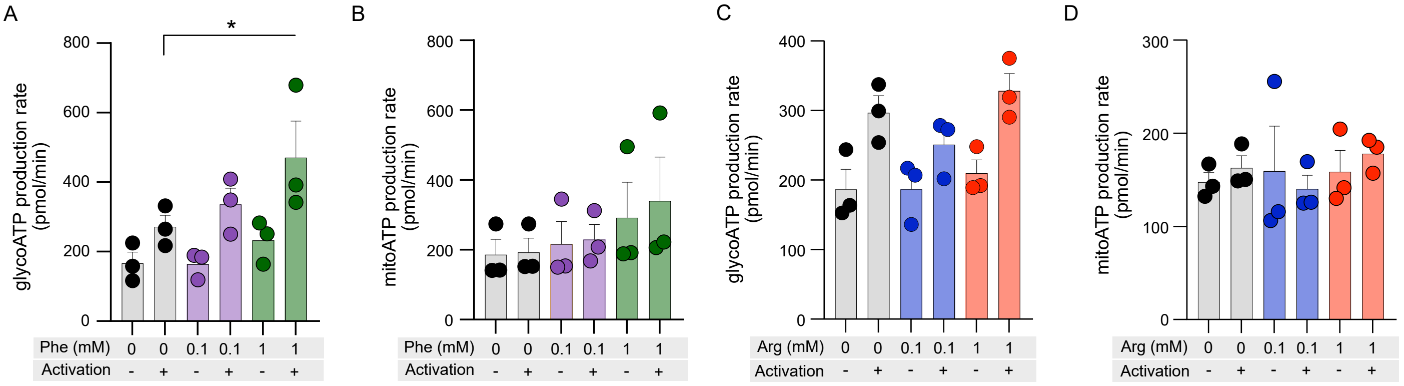

Figure S8. Quantification of induced glycoATP and mitoATP rates upon L-phenylalanine and L-arginine supplementation in *in vitro* differentiated Th2 cells. Related to figure 5.
(A-B) Quantification of induced glycoATP rate **(A)** and induced mitoATP rates **(B)** of *in vitro* differentiated Th2 cells incubated in full culture medium with/without additional Phe at concentrations of 0.1mM, 1mM, or Vehicle for 24h with/without acute CD2, CD3 and CD28 antibodies activation. **(C-D)** Quantification of induced glycoATP rate **(C)** and induced mitoATP rates **(D)** of *in vitro* differentiated Th2 cells incubated in full culture medium with/without additional Arg at concentrations of 0.1mM, 1mM, or Vehicle for 24h with/without acute CD2, CD3 and CD28 antibodies activation. Bars represent mean±SEM. Three independent experiments with 3 donors were conducted. Each dot represents the mean of more than 4 technical replicates per donor (n=3 different donors). One-way ANOVA with Sidaks´s multiple comparison correction was used to assess statistical significance. *p<0.05.

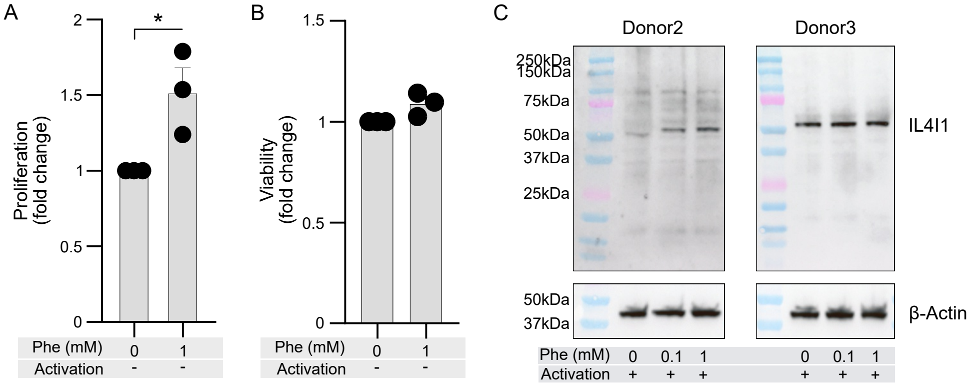

Figure S9. Effect of L-phenylalanine on the on the expression of IL4I1 and proliferation of *in vitro* differentiated Th2 cells. Related to figure 5. (A-B) Proliferation **(A)** and viability **(B)** of *in vitro* differentiated Th2 cells subjected to high doses of Phe. *In vitro* differentiated Th2 cells were incubated in full culture medium with or without additional supplementation of 1mM Phe without activation with CD2, CD3 and CD28 antibodies for 24h. Then, proliferation and viability were assessed using flow cytometry. Bar graphs show fold changes as compared to the Vehicle-treated, non-activated cells. Paired t-test was used for analysis. Each dot represents one donor (n=3 different donors). **(C)** Western Blot images depicting expression of IL4I1 in two additional different donors. *In vitro* long differentiated Th2 cells were incubated in media supplemented with 0.1mM and 1mM Phe or Vehicle with simultaneous activation using anti-CD2, anti-CD3 and anti-CD28 antibody coated beads for 24h. Subsequently, cells were harvested and lysed to isolate total RNA. Flow through from RNA isolation was stored and subjected to acetone precipitation to obtain total protein. WB was carried out with isolated protein using anti-IL4I1 primary antibody. Membrane was stripped and stained for β-Actin. β-Actin was used as loading control.

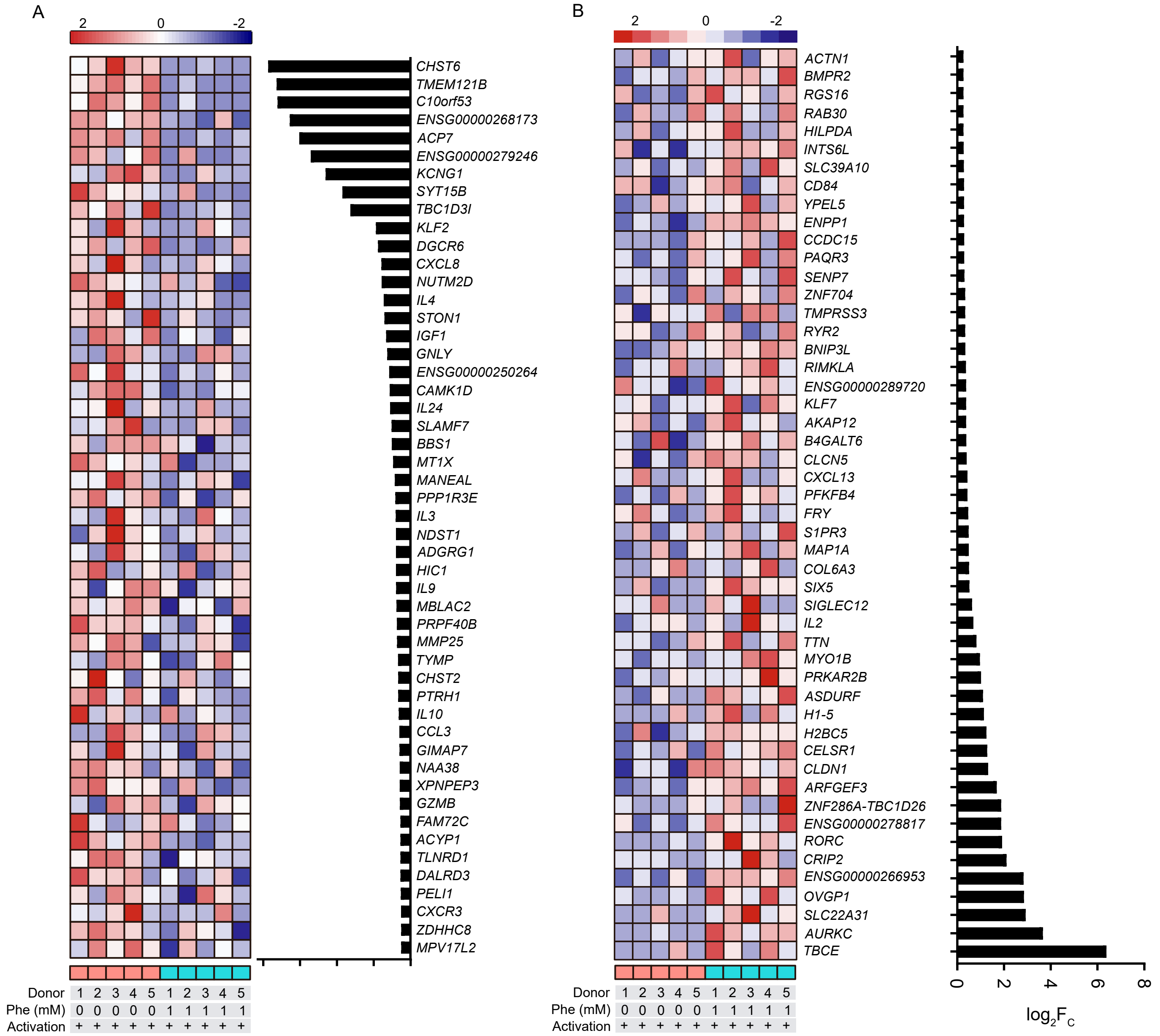

Figure S10. RNA sequencing of Phe-treated activated Th2 cells. Related to figure 5.
(A-B) Top 50 significantly downregulated **(A)** and upregulated **(B)** genes, by raw p-value < 0.05, depicted as a heatmap (left) and bar graph of fold changes (Log_2F_C) (right). Th2 cells from 5 different donors were incubated in media supplemented with 1mM Phe with or without simultaneous activation. These samples were harvested 24h later and subjected to RNA-sequencing.

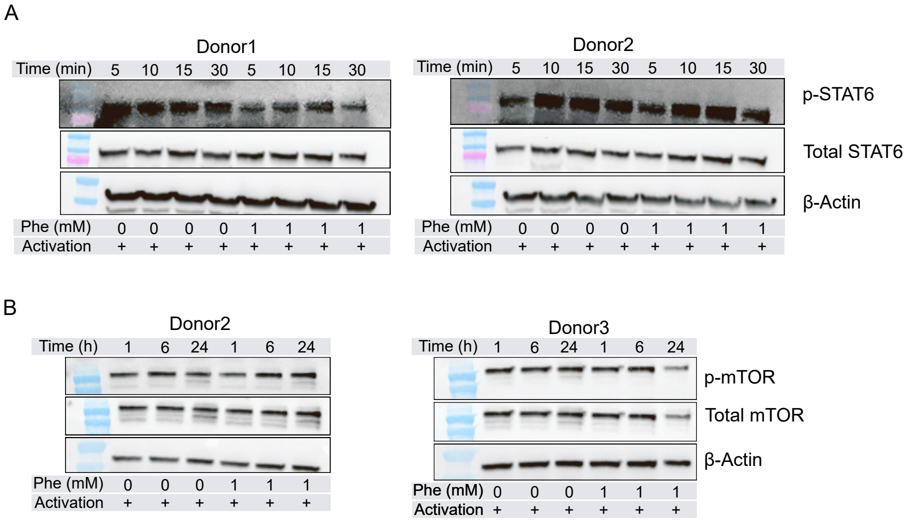

Figure S11. Effect of L-phenylalanine on phosphorylated and total STAT6 and mTOR. Related to figure 5.
(A-B) Western Blot images showing the expression of phosphorylated and total STAT6 **(A)**, and mTOR **(B)** in two additional different donors. *In vitro* long differentiated Th2 cells were incubated for mentioned time points in media supplemented with 1mM Phe or Vehicle with simultaneous activation using anti-CD2, anti-CD3 and anti-CD28 antibody coated beads. Subsequently, cells were harvested and lysed in RIPA containing protease and phosphatase inhibitors. WB was carried with cell lysate using different primary antibodies with intermediate washing and stripping steps. β-Actin serves as loading control and calibrator for quantification.

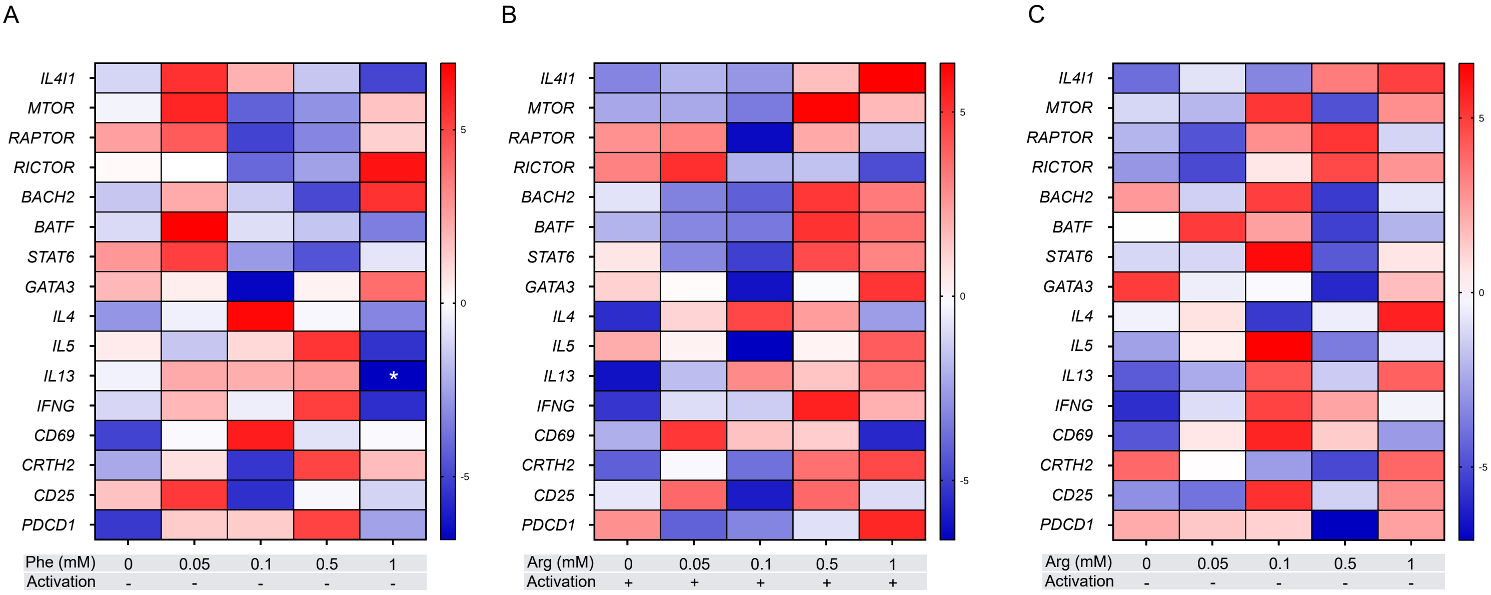

Figure S12. Effect of L-phenylalanine and L-arginine on the mRNA expression of type 2 transcription factors, cytokines, enzymes, and surface markers in Th2 cells. Related to figure 5.

(A-C) mRNA expression of transcription factors, cytokines, enzymes, and surface markers in in vitro differentiated Th2 cells treated with increasing doses of Phe **(A)**, and Arg **(B-C)** with **(B)** or without **(A, C)** activation with CD2, CD3 and CD28 antibodies for 24h.Th2 cells were incubated in full cell culture medium supplemented with Vehicle, 0.05mM, 0.1mM, 0.5mM and 1mM Phe. Following incubation, total RNA was isolated, and mRNA expression was determined using qRT-PCR. Data are analysed using One-way ANOVA with Dunnett's correction (n=6-8 different donors). Subsequently, z-scores were determined and plotted as heatmaps with individual genes represented in rows. Data are row normalized. *p<0.05.

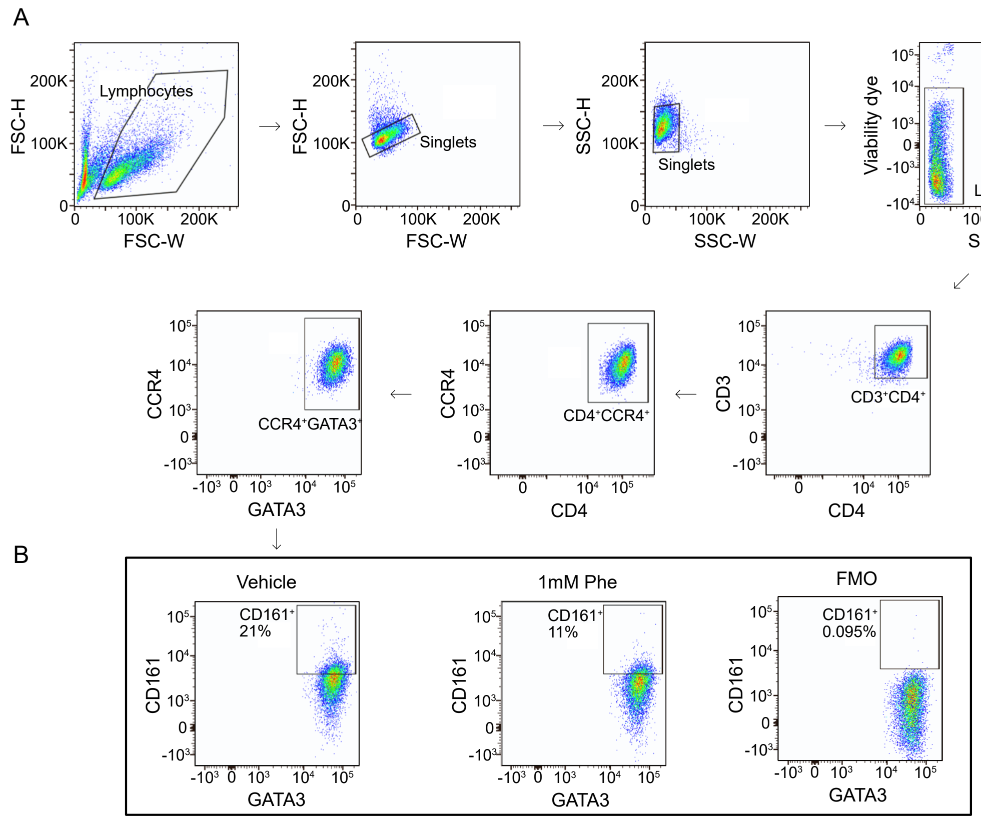

Figure S13. Effect of L-phenylalanine supplementation on Th2a cells. Related to figure 5.
(A) *In vitro* differentiated Th2 cells were incubated in media with or without supplementation of 1mM Phe for 24h with or without CD2, CD3 and CD28 activation antibodies. Following incubation, they were stained with panel provided in Table S8 and acquired on the flow cytometer. In short, live cells were negatively selected following elimination of doublets. $CD3^+CD4^+$ double positive cells were subsequently gated. Within this population, $CCR4^+$, $GATA3^+$ cells were positively selected and the frequency of $CD161^+$ Th2 (Th2a) cells was assessed in this population. **(B)** Representative flow cytometry plots showing the frequency of $CD161^+$ cells within $CD3^+CD4^+CCR4^+GATA3^+$ Th2 cells from one donor incubated in media additionally supplemented with Vehicle (left) or 1mM Phe (center). FMO control for CD161 signal has been included on the right. Pooled data from 3 different donors are shown in Figure 5P.

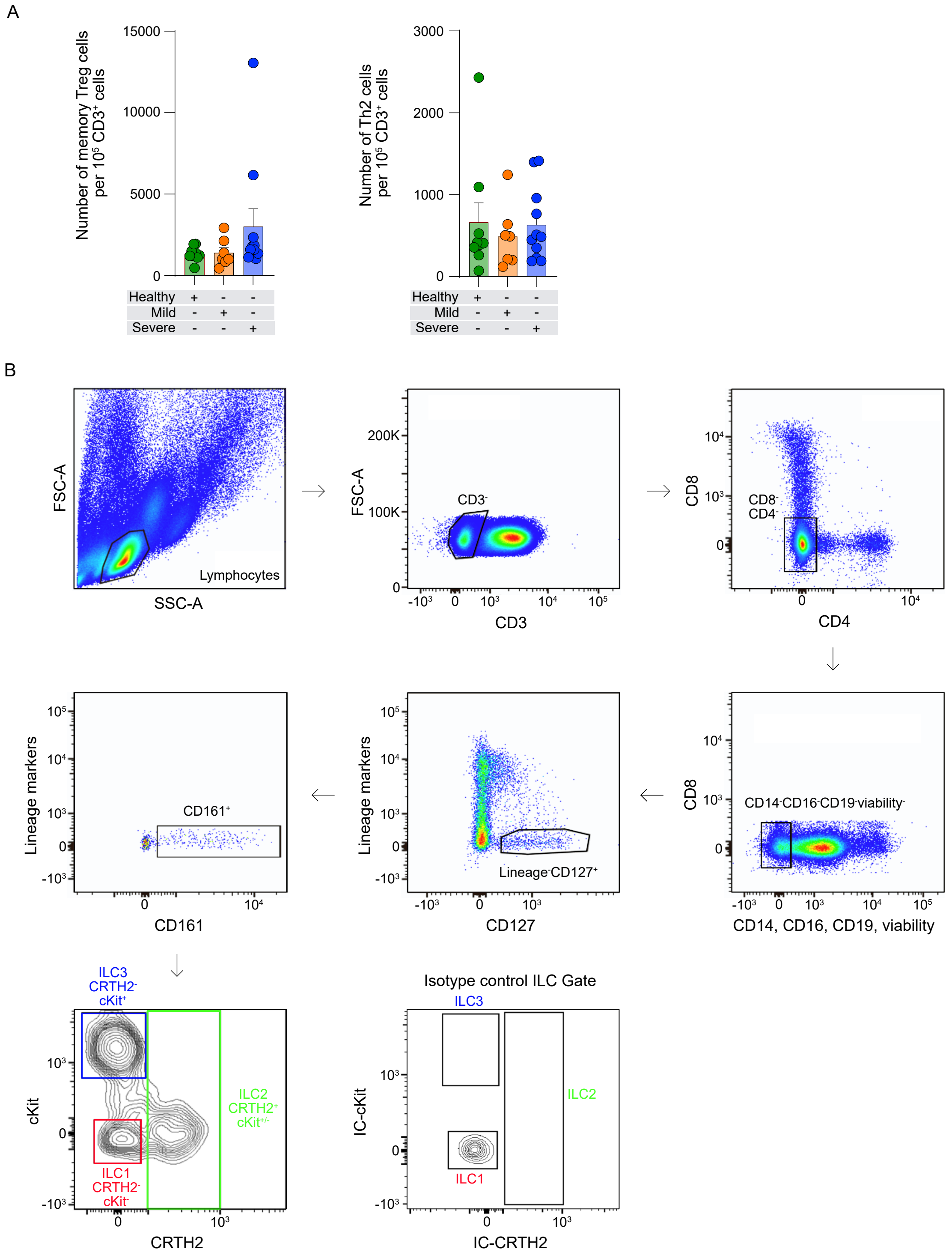

Figure S14. Flow cytometric assessment of memory cell populations and gating strategy for the study of ILCs in Cohort A samples. Related to figure 6. (A) Counts of memory Treg cells (left) and total Th2 cells (right) in healthy subjects (n=9), mild-allergic patients (n=7) and severe allergic patients (n=11) from participants in Cohort A. Counts were determined using flow cytometry and analysed with One-way ANOVA with Tukey correction. Bars represent mean±SEM and each dot represents one donor. **(B)** Gating strategy for assessment of innate lymphoid cells (ILCs) followed in classical analysis. ILCs were gated by eliminating doublets and all other immune cell lineages. Within $CD127^+$ and $CD161^+$ cells different classes of ILCs could be discerned by differential expression of CRTH2 and cKit: ILC1 ($CRTH2^-$, $cKit^-$), ILC2 ($CRTH2^+$, $cKit^{+/-}$), and ILC3 ($CRTH2^-$, $cKit^+$).

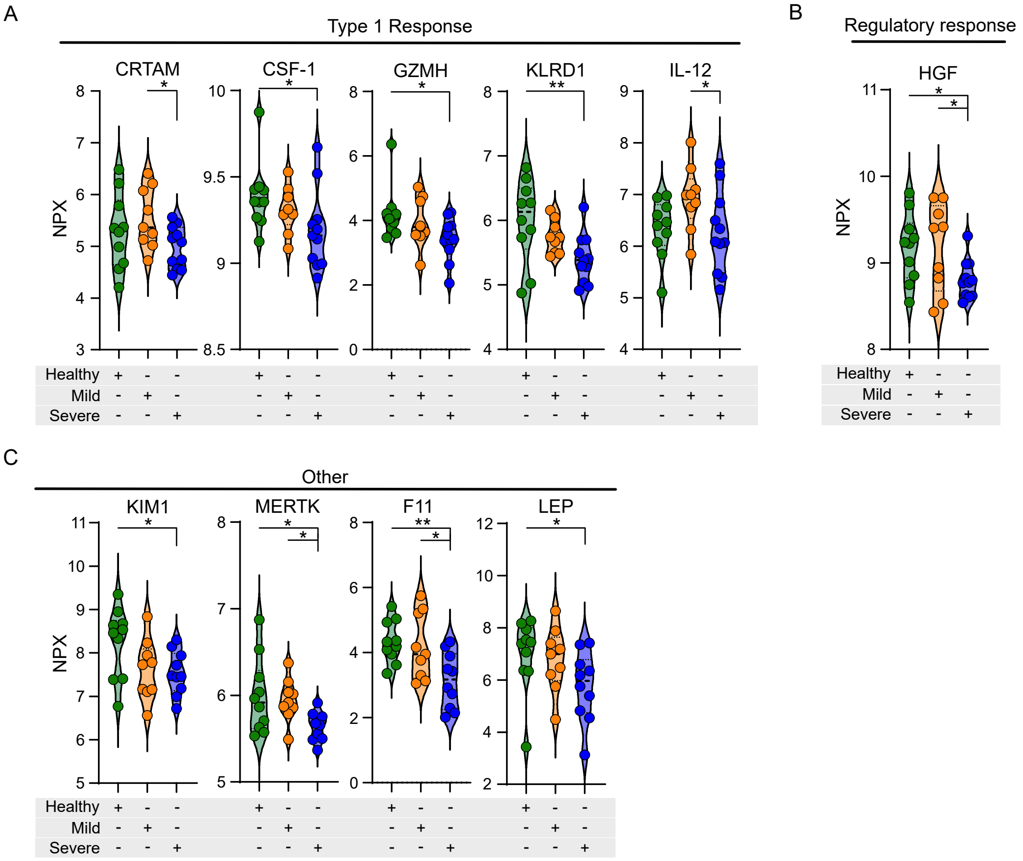

Figure S15. Systemic type 2 inflammatory profile of subjects from Cohort A by serum proteomics. Related to figure 6.
(A-C) Violin plots of differentially expressed type 1 response **(A)**, regulatory response **(B)** and key inflammation associated **(C)** proteins in the serum of controls (n=10), mild (n=9) and severe (n=10) allergic patients (Cohort A) analyzed by Proximity Extension Assay (PEA) and presented as NPX. One-way ANOVA with Fishers LSD test was used to compare differences between groups. All bars represent the mean±SEM and each dot represents one donor. *$p<0.05$, **$p<0.01$.

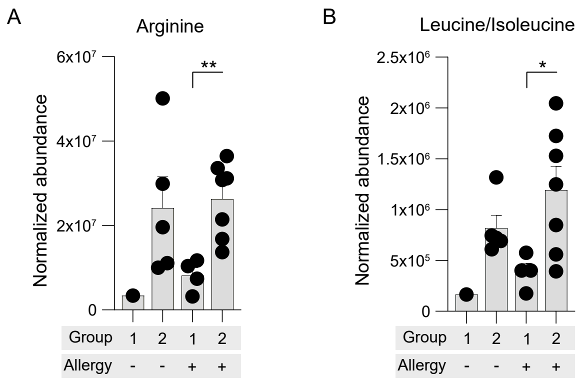

Figure S16. Quantification of Arg and leucine/isoleucine in Teff cells by metabolomics of Cohort A subjects. Related to figure 6. **(A-B)** Abundance of Arg **(A)** and Leu **(B)** in group 1 (control, n = 1; severe allergy, n = 4) and group 2 (control, n = 5; mild allergy = 1, severe allergy, n = 6) (subset of cohort A). Unpaired t-test was used to compare differences between groups. All graphs represent the mean±SEM. *p<0.05, **p<0.01.

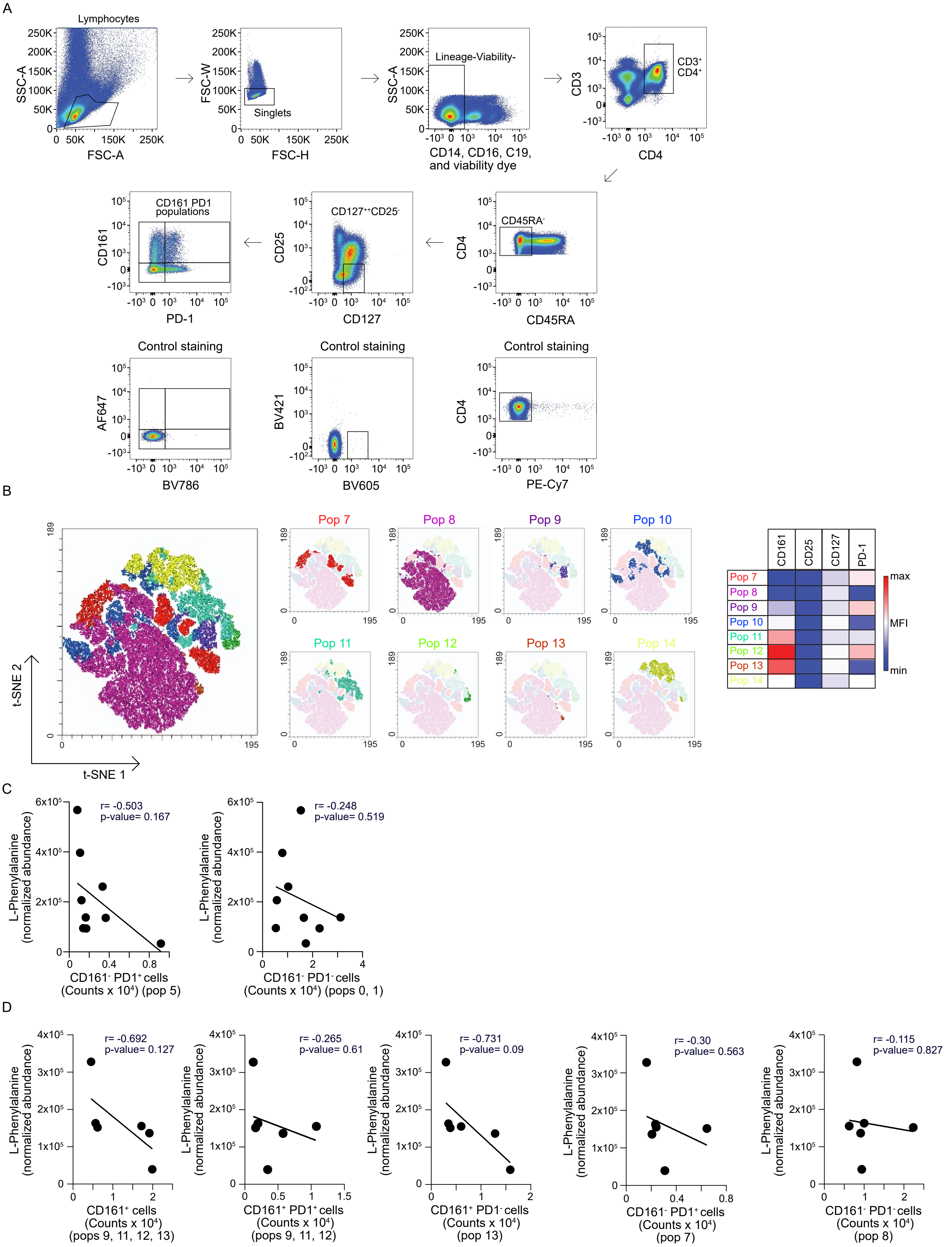
A
Lymphocytes
SSC-A
FSC-A
FSC-W
FSC-H
Singlets
Lineage-Viability-
CD14, CD16, C19, and viability dye
CD3
CD4
CD3+ CD4+
CD45RA-
CD45RA
CD127++CD25-
CD25
CD127
CD161 PD1 populations
CD161
PD-1
Control staining
AF647
BV786
BV421
BV605
PE-Cy7
B
Pop 7
Pop 8
Pop 9
Pop 10
Pop 11
Pop 12
Pop 13
Pop 14
t-SNE 1
t-SNE 2
CD161
CD25
CD127
PD-1
max
MFI
min
C
r= -0.503
p-value= 0.167
L-Phenylalanine (normalized abundance)
CD161- PD1+ cells (Counts x 10^4) (pop 5)
r= -0.248
p-value= 0.519
CD161- PD1- cells (Counts x 10^4) (pops 0, 1)
D
r= -0.692
p-value= 0.127
CD161+ cells (Counts x 10^4) (pops 9, 11, 12, 13)
r= -0.265
p-value= 0.61
CD161+ PD1+ cells (Counts x 10^4) (pops 9, 11, 12)
r= -0.731
p-value= 0.09
CD161+ PD1- cells (Counts x 10^4) (pop 13)
r= -0.30
p-value= 0.563
CD161- PD1+ cells (Counts x 10^4) (pop 7)
r= -0.115
p-value= 0.827
CD161- PD1- cells (Counts x 10^4) (pop 8)

Figure S17. Unbiased 2-dimensional flow cytometric analysis of memory CD4$^+$Teff cells from severe allergic patients and healthy controls (subset of Cohort A). Related to figure 6. (A) Gating strategy used for t-SNE and subsequent classical analysis to determine frequencies of populations based on expression levels of CD161, and PD-1 by flow cytometric assessment. **(B)** tSNE plot of unbiased 2-dimensional flow cytometric analysis of memory CD4$^+$Teff cells (CD3$^+$CD4$^+$CD45RA$^-$CD127$^+$CD25$^-$) from healthy controls (subset of Cohort A) identifying seven subpopulations based on the expression levels of CD161, and PD-1. **(C)** Pearson correlation of normalized abundance of intracellular Phe, measured in memory CD4$^+$Teff cells from patients with severe allergy, with total counts of CD161$^-$ populations within memory CD4$^+$Teff cells (subset of Cohort A). **(D)** Pearson correlation of normalized abundance of intracellular Phe, measured in memory CD4$^+$Teff cells from healthy controls, with total counts of CD161$^+$ and CD161$^-$ populations within memory CD4$^+$Teff cells (subset of Cohort A).

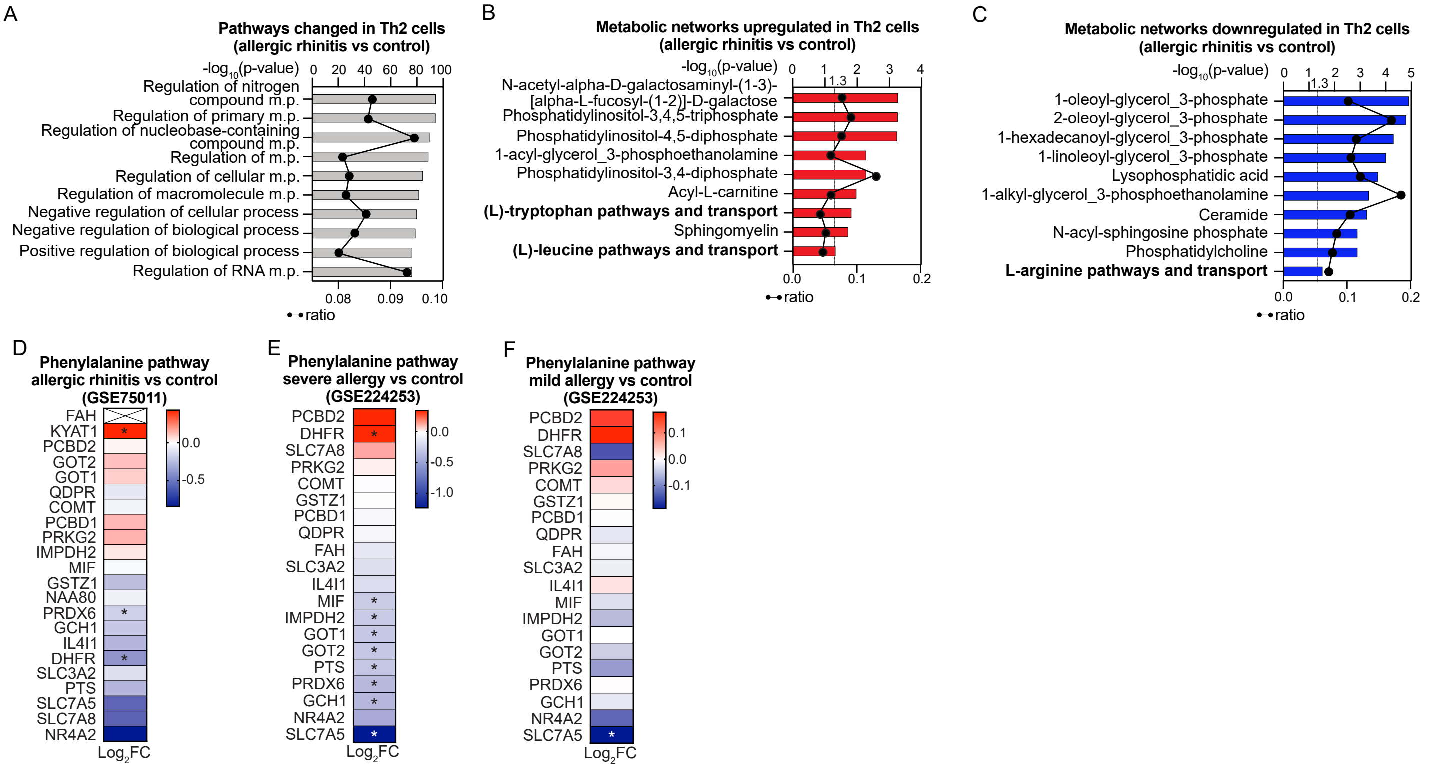

Figure S18. Expression of L-Phenylalanine metabolism related genes in previously published datasets (Cohorts B and C). Related to figure 7. (**A-C**) Top significantly enriched pathways (**A**), significantly upregulated (**B**) and significantly downregulated (**C**) metabolic networks within differentially expressed genes (DEG, $p<0.05$) in allergic rhinitis patients compared to controls (control n=15, allergic rhinitis n=25) from GSE75011[2] (Cohort B). Black line represents ratio of genes in experiment over complete pathway set. All data available in tables S21, S23 and S25, respectively. (**D**) Phe metabolism and transport pathway heatmap showing fold change (log2FC) of differentially expressed genes in allergic rhinitis patients (n=25) compared to controls (n=15) from GSE75011[2] (Cohort B). (**E**) Significant differentially expressed genes related to Phe metabolism in severe allergy patients (n=7) in comparison to healthy controls (n=8) from GSE224253[3] (Cohort C). (**F**) Significant differentially expressed genes related to Phe metabolism in mild allergy patients (n=9) in comparison to healthy controls (n=8) from GSE224253[3] (Cohort C). (B-F) Upregulated and downregulated genes are shown in red and blue, respectively. (D-F) Pathway curated and adapted from GSEA and MSigDB Database.

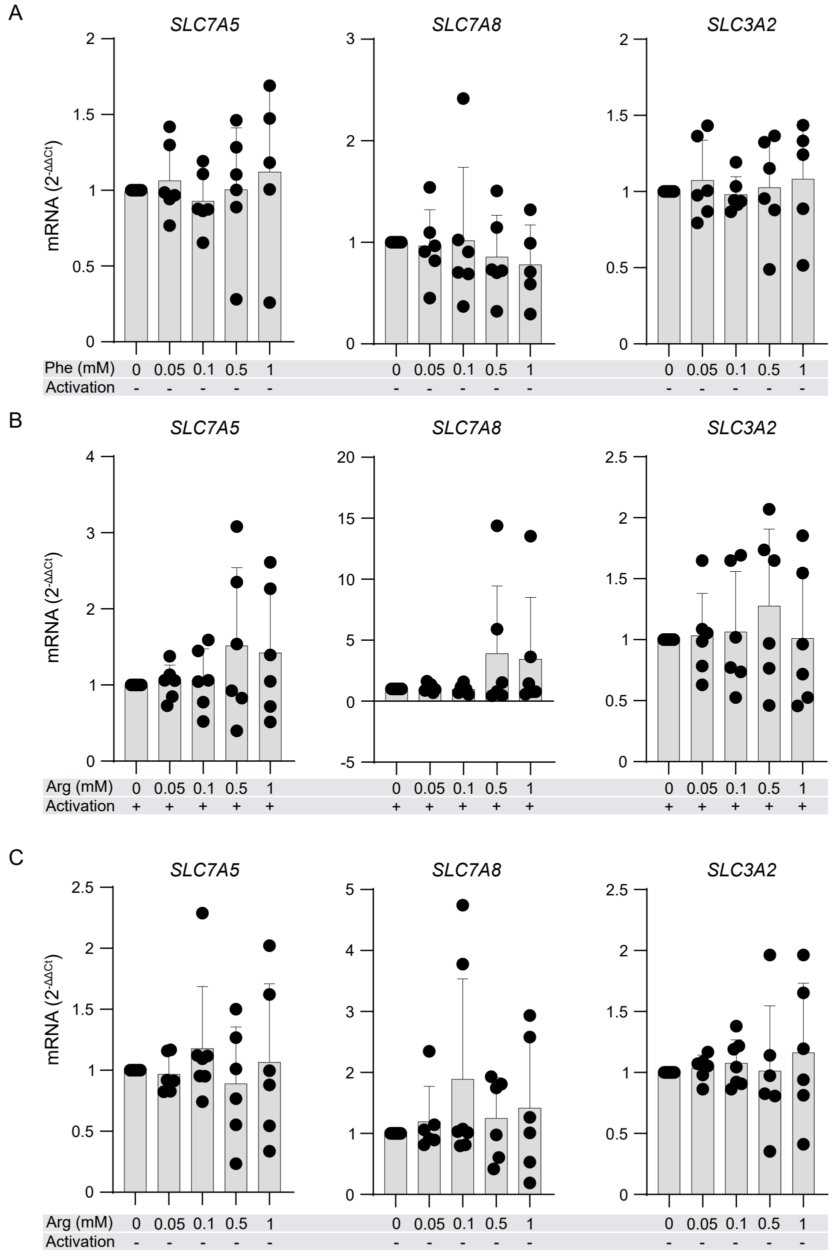

Figure S19. Effect of L-phenylalanine and L-arginine on the mRNA expression of *SLC7A5* (LAT1), *SLC7A8* (LAT2) and *SLC3A2* (CD98) in Th2 cells. Related to figure 7. (A-C) *In vitro* differentiated Th2 cells were treated with additional Phe without CD2, CD3 and CD28 activation **(A)** and additional Arg with **(B)** or without **(C)** CD2, CD3 and CD28 activation for 24h. Following incubation, mRNA expression was determined using qRT-PCR (n=6-8 different donors). Data are analysed using One-way ANOVA with Dunnett's correction (n=6-8 different donors). Bars represent mean±SEM.

Supplemental references list:

1. Cousins, D.J., Lee, T.H., and Staynov, D.Z. (2002). Cytokine coexpression during human Th1/Th2 cell differentiation: direct evidence for coordinated expression of Th2 cytokines. J Immunol *169*, 2498-2506. 10.4049/jimmunol.169.5.2498.

2. Seumois, G., Zapardiel-Gonzalo, J., White, B., Singh, D., Schulten, V., Dillon, M., Hinz, D., Broide, D.H., Sette, A., Peters, B., and Vijayanand, P. (2016). Transcriptional Profiling of Th2 Cells Identifies Pathogenic Features Associated with Asthma. J Immunol 197, 655-664. 10.4049/jimmunol.1600397.

3. Pablo-Torres, C., Garcia-Escribano, C., Romeo, M., Gomez-Casado, C., Arroyo Solera, R., Bueno-Cabrera, J.L., Del Mar Reano Martos, M., Iglesias-Cadarso, A., Tarin, C., Agache, I., et al. (2023). Transcriptomics reveals a distinct metabolic profile in T cells from severe allergic asthmatic patients. Front Allergy 4, 1129248. 10.3389/falgy.2023.1129248.
